# Whole genome sequencing data of 1110 *Mycobacterium tuberculosis* isolates identifies insertions and deletions associated with drug resistance

**DOI:** 10.1186/s12864-018-4734-6

**Published:** 2018-05-16

**Authors:** Xi Zeng, Jamie Sui-Lam Kwok, Kevin Yi Yang, Kenneth Siu-Sing Leung, Mai Shi, Zhiyuan Yang, Wing-Cheong Yam, Stephen Kwok-Wing Tsui

**Affiliations:** 1 School of Biomedical Sciences, The Chinese University of Hong Kong, Hong Kong SAR, China; 2 Hong Kong Bioinformatics Centre, The Chinese University of Hong Kong, Hong Kong SAR, China; 3 Department of Microbiology, The University of Hong Kong, Hong Kong SAR, China; 4 Centre for Microbial Genomics and Proteomics, The Chinese University of Hong Kong, Hong Kong SAR, China

**Keywords:** Mycobacterium tuberculosis, Antibiotics, Drug resistance, Insertion and deletion

## Abstract

**Background:**

Drug resistance in *Mycobacterium tuberculosis* (MTB) is one of the major challenges in tuberculosis (TB) treatment. However, known mutations cannot explain all of the cases of resistance and little research has focused on the relationship between insertions / deletions (indels) and drug resistance.

**Results:**

Here, we retrieved whole genome sequencing data of 743 drug-resistant MTB strains and 367 pan-susceptible strains from TB patients from the public domain to identify novel genomic markers of drug resistance. A total of 20 region markers containing genes and intergenic regions (IGRs) with significant statistical correlation with antibiotic resistance were revealed, four of which have been previously reported to be associated with drug resistance. In addition, 83 point markers containing frameshift (FS) mutations and IGR indels were also identified independently based on differences in their incidence rates between drug-sensitive and -resistant strains. Among the 83 point markers, eight indels were detected in known drug-associated genes or IGRs. Furthermore, the overlap between 20 region markers and 83 point markers further indicated their associations with drug resistance. The markers identified were involved in essential bacterial metabolic functions, including cell wall and transmembrane transporter functions. A strong correlation between FS mutations and mutations in DNA repair genes including *I21V* in *alkA*, *R48G* in *mutT4* and *P2R* in *nth* was also found.

**Conclusions:**

This study identified a set of novel genetic markers with FS mutations and IGR indels associated with MTB drug resistance, which greatly broadens the pool of mutations related to MTB drug resistance. This insight may be important in identifying novel mechanisms of drug resistance in MTB.

**Electronic supplementary material:**

The online version of this article (10.1186/s12864-018-4734-6) contains supplementary material, which is available to authorized users.

## Background

Tuberculosis (TB) is the top killer among infectious diseases in the world. In 2016, the estimated number of new TB cases was 10.4 million, and 1.7 million TB-related death were reported [1]. At present, TB drug resistance is one of the main difficulties facing TB treatment. Drug-resistant TB includes mono-resistant TB, multidrug-resistant TB (MDR-TB) and extensively drug-resistant TB (XDR-TB). According to a survey in China, about 34.2% of newly diagnosed TB cases and 54.5% of the previously treated cases were resistant to at least one drug [2].

Genetic mutations in the *Mycobacterium tuberculosis* (MTB) genome play key roles in the mechanism of MTB drug resistance. Great effort and progress has been made in investigating the role of non-synonymous single nucleotide polymorphisms (SNPs) in the mechanism of MTB drug resistance [3]. The involvement of non-synonymous SNPs in MTB drug resistance is better established than that of genomic insertions/deletions (indels). However, a few studies have already reported associations between genomic indels and MTB drug resistance. A previous study reported that a frameshift (FS) mutation occurred in *pncA* corresponding to amino acid 152 [4]. In another study, Zhang et al. examined 87 isoniazid (INH)-resistant strains and identified FS mutations in *katG*, *iniB* and *iniC* in three, two and two of these strains, respectively, but not in any of the INH-sensitive strains. They also found a non-synonymous mutation *S315 T* in the same strain harboring a *katG* FS mutation and this strain was highly resistant to INH (MIC = 64 mg/L) [5]. In recent years, FS mutations were also discovered in pyrazinamide (PZA) [6], streptomycin (STR) [7], INH, ethambutol (ETH) [8] and capreomycin (CAP) [9] resistant strains. The discovery of indels in drug-resistant strains inferred their role in MTB drug resistance. However, the involvement of indels in MTB drug resistance remains largely unknown, and a considerable proportion of TB drug resistant cases still cannot be explained [3, 10].

Considering that the mechanisms of MTB drug resistance are not completely understood and that the role of indels in drug resistance have not been fully elucidated, we hypothesized that there may be more indels to be discovered that are related to MTB drug resistance. To detect novel genomic markers of indels associated with MTB drug resistance, we retrieved the whole genome sequencing (WGS) data of 1110 clinical strains of MTB from the public domain to explore the relationship between indels and drug resistance on a large scale. To the best of our understanding, this is the first comprehensive and systematic bioinformatics study focusing on the association between MTB drug resistance and indels, including FS mutations and intergenic region (IGR) indels.

## Results

### Overview of the drug profiles

Raw MTB WGS data of 1110 MTB strains were obtained, of which 743/1110 (66.9%) belonged to drug-resistant MTB strains, and 367/1110 (33.1%) belonged to drug-sensitive MTB strains. In this study, we classified the isolates as MDR-TB, XDR-TB or DR-TB (drug resistant strains other than MDR-TB and XDR-TB). Among the drug resistant strains, there were 494/743 (66.5%) MDR-TB strains, 67/743 (9%) XDR-TB strains and 182/743 (24.5%) DR-TB strains (Additional file 1: Figure S1).

### Comparison of the indel counts between resistant and sensitive strains

**Fig. 1:**
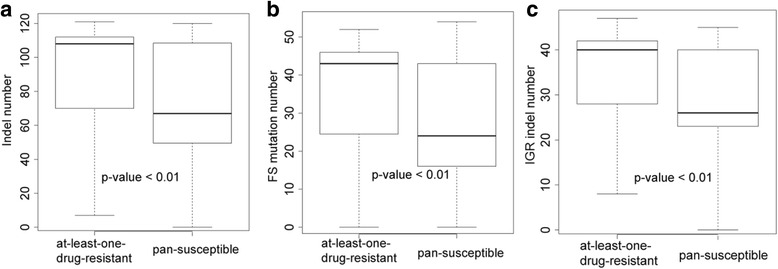
Comparisons of the number of indels for at-least-one-drug-resistant and pan-susceptible strains. The distribution of numbers of frameshift indels was shown. Wilcoxon rank sum test was used to test the differences for at-least-one-drug-resistant MTB strains and pan-susceptible MTB strains. The *p*-values refer to corrected p-values. (**a**) The comparisons for the number of all types of indels for at-least-one-drug-resistant and pan- susceptible strains. (**b**) The comparisons for the number of FS mutations for at-least-one-drug-resistant and pan- susceptible strains. (**c**) The comparisons for the number of IGR indels for at-least-one-drug-resistant and pan- susceptible strains

In this study, at-least-one-drug-resistant strains refers to the strains with resistance to at least one of the 12 TB drugs listed in our drug profile and pan-susceptible strains refers to the strains without any resistance to the 12 TB drugs. To investigate the difference in the indel numbers between the at-least-one-drug-resistant and pan-susceptible strains, the number of indels for the at-least-one-drug-resistant and pan-susceptible strains were calculated and compared with the Wilcoxon rank sum test. Type I errors arising from multiple testing were controlled by using the false discovery rate (FDR). The mean indel numbers for at-least-one-drug-resistant and pan-susceptible strains were 94 and 72 respectively (94 vs. 72, adjusted *p*-value < 0.01, Wilcoxon rank sum test) (Fig. 1a). An average of 37 and 27 FS mutations were detected for individual at-least-one-drug-resistant strains and pan-susceptible strains, respectively (37 vs. 27, adjusted p-value < 0.01, Wilcoxon rank sum test) (Fig. 1b). Furthermore, an average of 36 and 29 IGR indels were identified for at-least-one-drug-resistant and pan-susceptible strains, respectively (36 vs 29, adjusted p-value < 0.01, Wilcoxon rank sum test) (Fig. 1c). The IGR was defined as the genomic region starting from the last nucleotide of the upstream gene to the first nucleotide, which is the transcription start site of the downstream gene. The results of statistical tests showed that the number of all types of indels, FS mutations and IGR indels of the at-least-one-drug-resistant strains were all significantly higher than those of the pan-susceptible strains (Fig. 1).

To assess the associations between indels and resistance to each antibiotic, the indel numbers of drug-resistant and -sensitive strains were compared for each antibiotic. The *p*-value was adjusted by the FDR method. For FS mutations, indel numbers were significantly higher in resistant isolates than in sensitive isolates (Adjusted p-value < 0.01, Wilcoxon rank sum test) for all drugs except amikacin (AMI), kanamycin (KAN) and ETH. For IGR indels, significantly higher numbers of FS mutations were detected in resistant strains than in sensitive strains for all drugs except AMI, KAN and ETH (Adjusted *p*-value < 0.01, Wilcoxon rank sum test) (Additional file 1: Figure S2 and S3).

### Analysis of the differences in the indel incidence rates between drug- resistant and drug-sensitive strains

In this study, the incidence rate refers to the ratio of MTB strains having certain mutations. To check whether the incidence rates of FS mutations differed between resistant and sensitive strains for each drug, the incidence rate of FS mutations were calculated and the differences between the incidence rates of resistant and sensitive strains for each drug were tested. FDR method was used for multiple testing p-value adjustment. The results showed that the incidence rate of FS mutations in resistant strains was significantly higher for the drugs EMB, INH, moxifloxacin (MOX), ofloxacin (OFX), prothionamide (PRO), rifampin (RIF) and STR compared with the sensitive isolates (Table 1). To further explore the relationship between IGR indels and drug resistance, the incidence rates of IGR indels were also compared between resistant strains and sensitive strains for each drug. Strains resistant to ETH, KAN, MOX and PRO showed significantly higher incidence rates of IGR indels than strains that were sensitive to these drugs (Additional file 2: Table S1). In addition, significant differences were also found for the FS mutation incidence rate as well as the FS mutation numbers between each of the DR-TB, MDR-TB and XDR-TB groups (Additional file 2: Supplementary Results and Additional file 1: Figure S4).

**Table 1:** Tests for difference of FS mutation incidence rate between resistant and sensitive strains for each drug within 1110 MTB strains

Drug	Ratio of strains with FS mutation within resistant strains^a^	Ratio of strains with FS mutation within sensitive strains^b^	Adjusted Chi-square p-value	Adjusted Fisher’s exact p-value
AMI	0.83	0.77	0.323	0.477
CAP	0.82	0.77	0.323	0.471
**EMB**	**0.89**	**0.72**	**2.64E-09**	**8.46E-10**
ETH	0.93	**0.77**	0.017	0.020
**INH**	**0.87**	**0.63**	**3.08E-15**	**3.74E-15**
KAN	0.86	0.77	0.323	0.477
**MOX**	**0.90**	**0.76**	**0.002**	**0.001**
**OFX**	**0.89**	**0.76**	**4.63E-04**	**3.41E-04**
**PRO**	**0.86**	**0.76**	**0.006**	**0.006**
PZA	0.83	0.77	0.094	0.148
**RIF**	**0.87**	**0.67**	**2.43E-14**	**2.78E-14**
**STR**	**0.86**	**0.68**	**1.35E-11**	**1.62E-11**

Both chi-square test and fisher exact test were used to test the difference of the incidence rate between resistant and sensitive strains for each drug. The drugs with both chi-square p-value and fisher p-value lower than 0.01 were highlighted. FDR method was used to do multiple testing correction

^a^Incidence rate of FS mutation within resistant strains, which equals to the number of resistant strains with FS mutations divided by the total number of resistant strains of a certain drug

^b^Incidence rate of FS mutation within sensitive strains, which equals to the number of sensitive strains with FS mutations divided by the total number of sensitive strains of a certain drug

### Function enrichment analysis for genes with FS mutations specifically in at-least-one-drug-resistant strains

To identify the enriched pathways of genes with a high frequency of FS mutations in at-least-one-drug-resistant strains, the number of at-least-one-drug-resistant samples with FS mutations in each gene was calculated. Indels occurring in pan-susceptible strains were excluded. As a result, there were 222 genes containing FS mutations in more than two at-least-one-drug-resistant strains. The top 10 most frequently identified genes with FS mutations were *amiC*, *kdpD*, *Rv0278c*, *Rv0272c*, *ethA*, *Rv1509*, *pncA*, *gid*, *espK* and *Rv0738*. To investigate the relationship and of genes harboring drug resistance associated indels, the Database for Annotation, Visualization and Integrated Discovery (DAVID) analysis was performed using the 222 genes to identify functionally enriched pathways [11]. The significant pathways contained “IPR016035:Acyl transferase/acyl hydrolase/lysophospholipase” and “IPR011032:GroES-like” (Additional file 2: Table S2). Acyl transferase was an important catalyst in the synthesis of the cell wall and the GroES-like superfamily was also involved in cell wall biogenesis [12–14].

### Identification of region markers (genes and IGRs) carrying indels associated with drug resistance

**Fig. 2:**
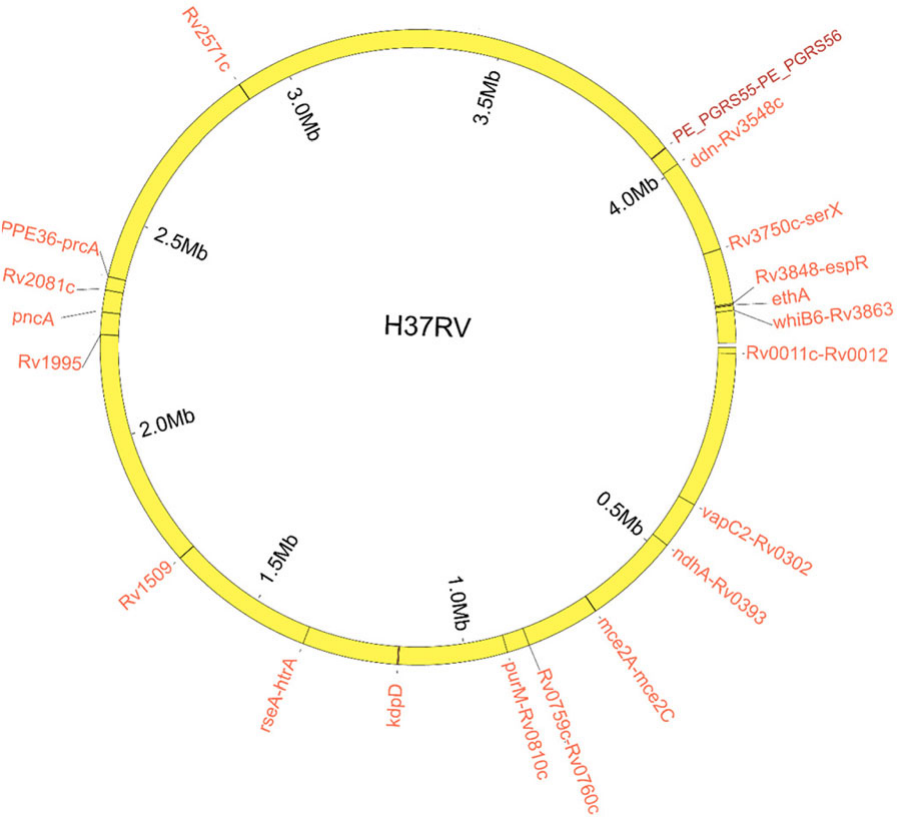
Genomic locations of the 20 region markers of drug resistance

In previous paragraphs, a general inference of association between MTB drug resistance and indels from a whole-genome perspective has been established. Function enrichment analysis also found that some resistance related functions were enriched in drug-resistant strain specific indels (Additional file 2: Supplementary Results, Tables S2 and S3, and Additional file 1: Figure S5-S6). To further identify the exact indel markers for MTB drug resistance, we hypothesized that (i) genes or IGRs associated with drug resistance should be diversifying, i.e., they should have a higher density of FS or IGR indels in genes or IGRs, respectively, than non-associated genes or IGRs, and (ii) that such genes or IGRs should be mutated with FS mutations or IGR indels more frequently in drug-resistant isolates than in drug-sensitive isolates. Indels occurring in repetitive elements, including genomic repeat regions, transposases, PE/PPE and PGRS genes, and phiRV1 members, were excluded [15]. We repeated our analysis drug by drug, grouping all isolates resistant to a given drug and using all isolates sensitive to that drug as controls. After further filtering of phylogenetically related regions, which were defined as those that contain FS/IGR indels only within one clade of the phylogenetic tree, 20 region markers were finally obtained, including seven genes and 13 IGRs, among which there were four known drug resistant associated markers, *pncA*, *ethA*, *whiB6-Rv3863* and *PPE13-Rv0879c* (Fig. 2, Additional file 2: Tables S4 and S5). The influence of population structure on the markers was assessed, and the results showed that the identified markers could be applied to different populations in this study (Additional file 2: Supplementary Results). There was a relatively big difference in the incidence rate of FS mutations in *ethA* between the ETH-resistant and ETH-sensitive strains (6/45 vs 36/1065, *p* < 0.01 for chi-square test, *p* = 0.051 for Fisher’s exact test). However, our criteria for reporting region markers included n Fisher’s exact test *p*-value of < 0.05 and a chi-square test p-value of < 0.05. Therefore, *ethA* was not reported as a marker for ETH resistance. Three (purM-Rv0810c, Rv3848-espR, PPE36-prcA) of the 14 drug IGRs express small RNAs (sRNAs). The indels in genes containing repetitive elements like PE/PPE genes have been removed. However, indels located in the intergenic regions (upstream or downstream) flanking by these genes were retained. That is why we could find *PPE36-prcA* in the result. Regarding the function of the identified region markers, 13 out of 20 identified resistance-associated genes/IGRs were related to the cell membrane and six genes/IGRs had functions related to cell wall biogenesis, cell organization or remodeling (Additional file 2: Tables S6 and S7). In addition, four out of 20 genes/IGRs were related to cell growth. Based on the analysis of these three categories in a whole-genome perspective, the cell membrane category was found to be significantly enriched (Additional file 2: Table S8).

### Identification of point markers of FS mutations and IGR indels associated with drug resistance

We then extended the identification of indel markers from region markers encompassing whole genes and intergenic regions to individual nucleotide positions of FS mutations and IGR indels, which we called point markers. For point markers, we identified FS mutations and IGR indels occurring more frequently in isolates resistant to a given drug [16]. After further filtering of phylogenetically related indels, which were defined as those that were present only within one clade of the phylogenetic tree, 83 point markers were finally obtained, consisting of 42 FS mutations and 41 IGR indels (See Methods and Additional file 2: Tables S9 and S10). The 83 point markers could be assigned onto 40 genes and 40 IGRs. A total of eight point markers were located in five known drug resistance associated genes (*mce1R*, *pks15*, *pncA*, *ppsA* and *Rv0658c*) and three IGRs (*ctpE-Rv0909*, *Rv1042c-Rv1043c* and *whiB6-Rv3863*) previously reported to be associated with MTB drug resistance [15, 16]. Among them, there were 10 (Additional file 2: Table S11) out of 40 IGRs expressing sRNAs, while one (*sigM*) out of the 40 genes was essential for cell growth [17, 18]. The functional distribution of genes harboring the point markers of FS mutations and genes adjacent to the point markers of IGR indels was similar to the functional distribution of the region markers (genes and IGRs). An exception was that one point marker of IGR indels was located in *Rv0194-Rv0195,* which was adjacent to genes coding for drug efflux pumps. (Additional file 2: Tables S12 and S13).

Among the 83 point markers (Additional file 2: Tables S9 and S10), six exclusively occurred in resistant strains (Additional file 2: Table S14) and 77 occurred both in drug resistant and sensitive strains. For example, among these six indels, the known drug resistant gene *pncA* was identified to have resistant-isolate-specific FS mutation *p.Val131fs*. The *p*.*Val131fs* mutation of *pncA* was identified in four strains out of 180 PZA-resistant strains but in none of the PZA-sensitive population (Additional file 2: Table S14). Furthermore, no known PZA resistant SNPs were found in the four PZA-resistant strains, suggesting that this FS mutation may play a role in conferring PZA resistance. Although the *p*.*Val131fs* mutation of *pncA* has not been reported before, other nonsynonymous and FS mutations have been reported in *pncA*, playing a critical role PZA resistance [19–21].

### Overlapping of the two types of drug resistant markers

To identify the most robust genomic markers of drug resistance, the overlaps between the two types of markers (20 region markers of genes / IGRs and 83 point markers of indels) were analyzed. Among the 83 point markers of FS mutations and IGR indels, two FS mutations and seven IGR indels were located in two out of seven region markers of genes and seven out of 13 region makers of IGRs (Table 2). For example, 517 out of 683 INH-resistant strains vs. 161 out of 427 INH-sensitive strains possessed indels in the IGR *mce2A-mce2C*, and 517 out of 683 INH-resistant strains vs 160 out of 427 INH-sensitive strains possessed the mutation 688,792:T:TG locating in *mce2A-mce2C*. Therefore, IGR mce2A-mce2C was identified as a region marker and the mutation 688,792:T:TG in mce2A-mce2C was identified as a point marker. *mce2A* and *mce2C* are integral components of the membrane and are involved in the ABC transporter pathway, which was reported to be related to anti-TB drug-resistance [22]. The overlaps between the two types of drug resistant markers generated by the two independent analyses further indicated their associations with drug resistance.

**Table 2:** Overlapping of region markers (genes/IGRs) and point markers (FS mutations and IGR indels)

Region markers	Mutation type	Point markers	Functions
ndhA-Rv0393	Intergenic region	472,711:T:TTTGTGGGCC^a^	NADH dehydrogenase NdhA, hypothetical protein
rseA-htrA	Intergenic region	1,365,837:C:CGG	anti-sigma E factor RseA, serine protease HtrA
Rv2081c	Frameshift mutation	p.Val105fs^b^	transmembrane protein
whiB6-Rv3863	Intergenic region	4,338,595:GC:G	transcriptional regulator WhiB6, hypothetical protein
vapC2-Rv0302	Intergenic region	364,498:TG:T	ribonuclease VapC2, transcriptional regulator
pncA	Frameshift mutation	p.Val131fs	pyrazinamidase/nicotinamidase PncA
Rv0759c-Rv0760c	Intergenic region	854,252:GC:G	hypothetical protein, hypothetical protein
mce2A-mce2C	Intergenic region	688,792:T:TG	Mce family protein Mce2A, Mce family protein Mce2C
Rv3750c-serX	Intergenic region	4,198,611:CG:C	excisionase, tRNA

The content in the 3rd column included “Location_in_genome:reference_allele:alternative_allele” representing IGR indels and “Change of amino acid” representing for FS mutations

^a^Location_in_genome:reference_allele:alternative_allele

^b^Change of amino acid

### Resolving the confounding effects of multi-drug resistance on the identified markers

In some cases, markers should be associated with the resistance to only one drug, but resistance to more than one drug was observed. This phenomenon was due to the existence of MDR-TB and XDR-TB strains. To resolve this confounding factor, a logistic regression model was used to re-analyze the identified markers. As a result, many associations of drug resistance and markers that were likely to be caused by confounding factors were resolved. All but one region marker and 10 point markers still showed significant associations (*p* < 0.05) with drug resistance (Additional file 2: Tables S15 and S16).

### Correlation of indels and DNA repair mutations

**Fig. 3:**
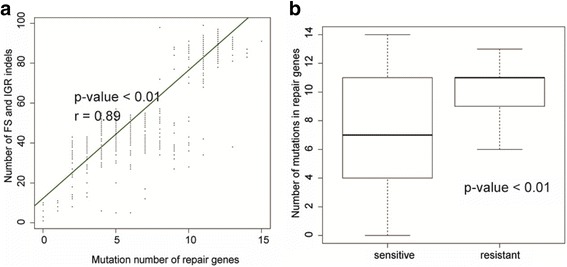
The relationships between DNA repair genes and indels. (**a**) Correlation between mutation number of repair genes and number of FS + IGR indel. (**b**) Contrast of DNA repair gene mutations between sensitive strains and resistant strains

To explore the mechanism responsible for the generation of indels, correlation analysis of indel numbers and mutations in DNA repair genes was conducted. The results showed that indel numbers were positively correlated with the number of mutations in DNA repair genes (Fig. 3a). In addition, we also found that the number of mutations in DNA repair genes was higher in resistant samples than that in sensitive samples (Fig. 3b). There were six DNA repair mutations with high frequencies among samples and large differences in frequency between resistant and sensitive samples. We found that, when one of the six DNA repair mutations was present, there were more FS mutations. Three of the six mutations were predicted to be deleterious (Additional file 2: Table S17), suggesting that the three mutations *nth:p.Pro2Arg, recD:p.Glu120Asp* and *uvrC:p.Val434Ala*, were likely to play a role in inducing indels and drug resistance.

### Validation using another data set

Finally, to investigate the validity of our approach, we analyzed another WGS data set originating from 765 at-least-one-drug-resistant strains and 2729 pan-susceptible MTB strains, distinct from the 1110 strains mentioned above. The results showed that the number of FS mutations in resistant strains was significantly higher than that of sensitive strains, as well as IGR indels. Strong correlations between DNA repair mutations and FS/IGR indels were consistently identified (Additional file 1: Figure S7). We also searched for the resistant markers in the validation data set and found that 13 out of the 20 region markers and 60 out of the 83 point markers were still significant in the validation set (Additional file 2: Tables S18 and S19).

## Discussion

The association between genomic indels and drug resistance has been reported in sporadic studies and few large scale studies have been reported that estimated the involvement of indels in the process of MTB drug resistance. Here, we made use of these large sets of WGS data to identify the association between MTB drug resistance and FS mutations and IGR indels.

Many of our identified markers have functions related to cell membrane, cell wall, and metabolism and transporter functions, which are closely related to MTB drug resistance. This is in line with the fact that stable drug resistance phenotypes may evolve through a complex stepwise process involving cell membrane remodeling [23]. On the one hand, among the identified markers, one region marker *ethA* and 6 point markers carried FS or IGR indels exclusively in resistant strains for a particular drug (Additional file 2: Tables S5 and S14). These markers possibly conferred drug resistance to MTB. For example, the region marker *ethA* out of 20 region markers was found to contain FS mutations exclusively in 42 out of 683 INH-resistant isolates but not in any INH-sensitive isolates. However, we cannot exclude the possible relationship between *ethA* and ETH resistance. The reason why *ethA* was not identified as a marker of ETH resistance was that its adjusted *p*-values were a bit higher than those specified by our criteria. On the other hand, there was a larger proportion of markers (19 region markers and 77 point markers) that occurred in both resistant and sensitive strains for a certain drug (Additional file 2: Tables S5 and Table S10). These markers may not cause drug resistance directly but rather provide an incremental fitness advantage to resistant strains and play compensatory roles in drug resistance isolates, including lowering the growth rate and helping the pathogen to survival in the environment following exposure to antibiotics [24, 25]. For example, the FS mutation *p.Asn28fs* in *pks6* occurred in 516 out of 683 INH-resistant strains and 158 out of 427 INH-sensitive strains (516/683 vs. 158/427, adjusted p-value < 0.01 for chi-square test, adjusted p-value < 0.01 for Fisher’s exact test). It was reported that mutations in the *pks* gene family, which encodes membrane-bound polyketide synthase possibly functioning in the transport of lipids and complex cell wall components, could affect drug susceptibility [16].

There have been few studies investigating the role of IGRs in MTB drug resistance. In our study, the IGRs were not ignored, potentially providing an alternative perspective to studies focusing only on coding regions. Eleven novel drug resistance–associated region markers of IGRs and 38 novel point markers of IGR indels were identified. Consistent with this, *Zhang* et al. previously reported drug resistance associated IGRs *thyA–Rv2765* and *thyX–hsdS*, whose SNPs increased the expression levels of downstream genes [9]. In addition, sRNAs encoded by IGRs can regulate gene expression to adapt to changes in the environment [18]. The results of our research along with previous findings suggest that IGRs have a role in MTB drug resistance and thus deserve further attention.

We also analyzed the presence of known resistant SNPs in the isolates included in the study. We found that within 730 resistant samples containing identified point markers in this study, 647 samples also possessed known resistant SNPs while 83 samples only had identified point markers. For example, for sample ERR144567, which was resistant to OFX, FS mutation *p.Arg491fs* in *pks15* and *P.Asn28fs* in *pks6* concurred with the well-known resistance conferring SNP A90V in *gyrA*, suggesting that these two FS mutations contributed to this drug resistance. The results inferred that point markers in the 647 resistant samples were more indicative to be compensatory to established mechanisms of resistance, rather than be the primary cause. We next performed an analysis to check whether any novel resistance-associated SNPs existed in the 83 samples possessing only indel markers. As a results, we found that 21 out of the 83 samples contained novel SNPs (*PPE38-PPE39*: 2634282:T:G and *eis-Rv2417c*: 2715344:G:A) associated with drug resistance. Therefore, 62 resistant isolates did not contain any resistant SNPs, suggesting that the point markers of FS mutation and IGR indels may be the primary cause of resistance (Table S20).

Finally, a correlation between number of indels and mutations in DNA repair genes was observed, suggesting that DNA repair gene mutations may be associated with MTB drug resistance. Furthermore, previous studies demonstrated that mutations in DNA repair genes may help MTB acquire mutations against anti-TB drugs. Many anti-TB drugs can upregulate the expression of DNA repair genes [26]. In support of our analysis, *alkA* and *mutT4* mutations have been detected in many resistant strains of Haarlem lineage and W-Beijing lineage respectively [27, 28].

This is the first study exploring the association between MTB drug resistance and indels based on large scale sequencing data. Many novel genes, IGRs and indels associated with drug resistance were identified. Our study demonstrates a new approach for future investigations into drug resistance mechanisms in both MTB and other bacterial pathogens treated with the similar antibiotic regimens.

## Conclusions

We utilized a large public dataset to correlate phenotype with genotype in MTB and detected 20 region markers and 83 point markers that were statistically associated with drug resistance. These findings provide novel insight into previously unknown mechanisms of drug resistance in MTB and will potentially assist in the battle against TB drug resistance, by improving the diagnosis of TB drug resistance and drug design in the future.

## Methods

### Retrieval of the sequencing data and drug resistance profiles of MTB clinical strains from four previous studies

First, 1110 clinical MTB strains with raw WGS data from three studies were downloaded from the NCBI Sequence Read Archive (SRA) database and the European Nucleotide Archive (ENA) database with the corresponding accession codes (SRA065095, SRA020129, SRA009637, SRA009341, SRA009458, and ERP000192) [15, 16, 29]. The drug-sensitive and -resistant isolates included in this study represent a wide range of geographic sources, MTB lineages and resistance profiles. Another WGS data set of 3494 clinical MTB strains was retrieved as a validation set [30]. Profiles are available for the following 12 drugs: INH, RIF, EMB, STR, CAP, KAN, PZA, ETH, AMI, OFX, PRO and MOX.

### Sequencing platforms and drug susceptibility testing methods for the used data

Samples from Farhat et al. [15] were collected worldwide and sequenced by an Illumina Genome Analyzer IIx instrument, whereas the samples from Casali et al. [29] and Zhang et al. [16] were collected from Russia and China, respectively. A total of 1040/1110 (93.7%) samples were sequenced using the HiSeq 2000 platform, while the rest were sequenced on an Illumina Genome Analyzer IIx instrument. As a validation data set, another WGS data set of 3494 clinical MTB strains from the United Kingdom, Sierra Leone, South Africa, Germany, and Uzbekistan was also obtained [30].

Among the 1110 strains, the drug susceptibility of 231/1110 strains (20.8%) was tested on solid media using the standard proportion method recommended by the World Health Organization. The drug susceptibility of 879/1110 strains (79.2%) was determined using the absolute concentration method on Lowenstein-Jensen slopes or using the automated Mycobacterial Growth Indicator Tube ((MGIT)) 960 system (Becton Dickinson). In addition, for the validation data set containing 3494 stains, the WHO-endorsed proportion method in an automated MGIT 960 system (Becton Dickinson) on solid Lowenstein-Jensen media, or the resistance ratio method was used to perform drug susceptibility testing.

### Reference mapping of the raw sequencing reads of 1110 genomes and the detection of indels

All of the downloaded sequencing reads were mapped onto the MTB H37Rv reference genome (Accession number: NC_000962.3) using Burrows-Wheeler Aligner (0.7.15) [31]. The PCR duplications were removed based on the alignment results using Samtools [32]. All the indels from 1110 strains were called using Platypus [33]. Indels or SNPs occurring in repetitive elements were filtered out, including genomic repeat regions, transposases, PE/PPE and PGRS genes, and phiRV1 members. SnpEff was then applied to annotate the genes [34].

### The detailed criteria for filtering indels

1. The fraction of reads mapping to the forward and reverse strands was significantly different.
2. The median of the minimum base quality score close to 7 bps on either side of a read was less than 15, or more than 70% of reads supporting a mutation were filtered out in the candidate generation stage of Platypus.
3. The root of the mean-square mapping quality of all reads covering a mutation site was less than 40.
4. The quality-over-depth score which was equal to QUAL divided by the number of reads supporting the mutation was less than 10.
5. Posterior quality of a mutation, as defined in Platypus, was below 20.
6. A mutation was detected in a low-complexity region (21 bp flanking region around the mutation site) where the contribution of the two most frequent nucleotides was more than 95%.
7. The indels defined as heterozygous (genotype 0/1) were filtered out and only homozygous indels (genotype 1/1) were reserved. For each indel sites, the most abundant base should be different from that in the H37Rv genome.

### Identification of drug resistance-associated region markers and point markers.

Two independent methods were incorporated in this task:

1. Genomic mutations including indels would be enriched in some genomic regions due to drug selection pressures. According to this hypothesis, we identified genomic regions including genes and IGRs that have a greater density of FS mutations or IGR indels than would be expected by a random distribution, in 743 resistant strains.
2. Genomic regions (genes and IGRs) and indels (FS and IGR indels) were identified if mutating more frequently in isolates resistant to a given drug than in all other isolates.

Methods (1) and (2) were used to identify region markers including genes and IGRs. Both the chi-square test and Fisher’s exact test were performed. FDR method was used to perform *p*-value adjustment. Genes or IGRs with both adjusted chi-square test *p*-values and adjusted Fisher’s exact test p-values < 0.05 in at least one of the 12 drug profile groups were reserved. Genes and IGRs identified by both methods (1) and (2) were selected.

Method (2) was used to identify the point markers including FS mutations and IGR indels closely associated with drug resistance. FS mutations and IGR indels presented in drug-resistant isolates with significantly higher frequencies than in drug-sensitive isolates (FDR adjusted chi-square test *p* < 0.01 and FDR adjusted Fisher’s exact test p < 0.01) in at least one of the 12 drug profile groups were reported.

### Logistic regression

To exclude the influences on lineage effect and the background drug resistance confounding effects of MDR or XDR and estimate the strength of the associations between markers and drug resistance, logistic regression was performed. The sequence of the drugs was estimated to be INH, RIF, PZA, STR, EMB, OFX, MOX, ETH, KAN, AMI, CAP and PRO, based on their order of use. Lineage effect was also considered. We then fitted a regression model of the form ‘resistance of interest~resistances earlier in sequence + lineage +drug resistant markers)’. For example, to estimate the strength of relationships between resistant marker Rv1509 and OFX resistance, we used the following model: OFX ~ INH + RIF + PZA + STR + EMB + lineage+Rv1509. The chi-square test was used to test the difference of residual deviation from the real data between the full model containing all testing factors and the reduced model without the drug resistance associated marker. If the chi-square p-value is significant, then we can conclude that the association between resistant marker Rv1509 and OFX resistance is existing.

### Phylogenetic tree construction

A superset of SNPs related to the reference strain H37Rv was created across all clinical isolates from the Platypus SNP reports. A maximum likelihood phylogeny was reconstructed with RAxML [35] using a general time reversible model with gamma correction for among-site rate variation. Calculation of 100 bootstrap replicates provided support for the nodes on the tree. The phylogenetic tree was visualized with FigTree (http:// tree.bio.ed.ac.uk/software/figtree).

## Additional files

Additional file 1: **Figure S1.** The profile of 1110 isolates. (a) The distribution of isolates in three studies. (b) The drug profiles for the 1110 isolates. **Figure S2.** Boxplot of the FS mutation numbers in the drug-resistant and -sensitive strains for each drug. The *p*-values refer to corrected *p*-values. **Figure S3.** Boxplot of the IGR indel numbers in the drug-resistant and -sensitive strains for each drug. The *p*-values refer to corrected *p*-values. **Figure S4.** Boxplot of the FS mutation numbers in the DR-TB, MDR-TB and XDR-TB groups of strains. The distribution of the number of FS mutations is shown. A Wilcoxon rank sum test was used to test the differences among the DR-TB, MDR-TB and XDR-TB groups. **Figure S5.** Protein-protein interaction for genes with frameshift indels in DR-TB strains. **Figure S6.** Protein-protein interaction for genes with frameshift indels in pan-susceptible strains comparing to DR-TB group. First, the FS mutations also existing in DR-TB strains were excluded. Then, the remained FS mutation in pan-susceptible strains were annotated onto the genes. The obtained gene list combined with known resistance-associated genes was inputted into STRING. The thicker the lines between two proteins, the stronger the relationship between them. The protein-protein interaction analysis took into consideration the following factors: the presence of fusion evidence, neighborhood evidence, co-occurrence evidence, experimental evidence, text mining evidence, database evidence and co-expression evidence. **Figure S7.** Validations using another data set. (a) Comparison of the FS indel numbers between drug-resistant and -sensitive strains. (b). Comparison of the IGR indel numbers between drug resistant and sensitive strains. (c). The Correlation between DNA repair mutation numbers and FS + IGR indel numbers. Each dot represents an *MTB* strain. (DOCX 10447 kb)

Additional file 2: **Table S1.** Test for the difference in IGR indel incidence rate between resistant and sensitive strains for each drug among 1110 MTB strains. **Table S2.** Function enrichment for genes with at-least-one-drug-resistant strain-specific FS mutations in more than two resistant strains. **Table S3.** Function enrichment for genes with frameshift mutations in the DR-TB, MDR-TB and XDR-TB groups of strains. **Table S4.** The 20 region markers identified according to adjusted chi-square and Fisher’s exact *p*-values. **Table S5.** The 20 identified region markers and corresponding strain numbers. **Table S6.** Overview of functions for 20 region markers. **Table S7.** Description of the 20 region markers. **Table S8.** The distribution of region markers in function categories. **Table S9.** The 83 identified point markers of FS mutations and IGR indels, showing adjusted *p*-values. **Table S10.** The 83 identified point markers of FS mutations and IGR indels, showing strain numbers. **Table S11.** The identified point markers located in the IGRs expressing sRNA. **Table S12.** Overview of the functions of the 83 point markers. **Table S13.** Descriptions of the functions of the 83 point markers. **Table S14.** The 6 point markers out of the 83 point markers exclusively occurring in resistant strains. **Table S15.** *P*-values after logistic regression for the associations between the 20 region markers and drug resistance. **Table S16.** *P*-values after logistic regressions for the associations between the 83 point markers and drug resistance. **Table S17.** Effect of mutations on DNA repair genes. **Table S18.** Region markers in the validation set overlapping with the 20 region markers. **Table S19.** Point markers in the validation set overlapping with the 83 point markers. **Table S20.** Point markers in the 62 samples in which no known drug resistance associated SNPs were found. **Table S21.** The incidence rate for region markers. **Table S22.** The incidence rate for point markers. (DOCX 193 kb)

Additional file 3: The accession numbers of the validation data set. (XLSX 79 kb)

## Abbreviations

AMI: Amikacin
CAP: Capreomycin
DR-TB: At-least-one-drug-resistant strains other than MDR-TB and XDR-TB
ENA: European Nucleotide Archive
ETH: Ethambutol
FS: frameshift
IGR: Intergenic region
Indels: insertions/deletions
INH: Isoniazid
KAN: Kanamycin
MDR-TB: Multidrug-resistant TB
MOX: Moxifloxacin
MTB: Mycobacterium tuberculosis
OFX: Ofloxacin
PRO: Prothionamide
RIF: Rifampin
SNPs: single nucleotide polymorphism
SRA: Sequence Read Archive
STR: Streptomycin
TB: Tuberculosis
XDR-TB: extensively drug-resistant TB
