# Supplementary material for: Whole genome sequencing data of 1110 Mycobacterium tuberculosis isolates identifies insertions and deletions associated with drug resistance
Source: BMC Genomics. 2018 May 16;19:365. doi: 10.1186/s12864-018-4734-6 (PMC5956929; doi:10.1186/s12864-018-4734-6)
Supplement: Supplementary file 1 — Figure S1. The profile of 1110 isolates. (a) The distribution of isolates in three studies. (b) The drug profiles for the 1110 isolates. Figure S2. Boxplot of the FS mutation numbers in the drug-resistant and -sensitive strains for each drug. The p-values refer to corrected p-values. Figure S3. Boxplot of the IGR indel numbers in the drug-resistant and -sensitive strains for each drug. The p-values refer to corrected p-values. Figure S4. Boxplot of the FS mutation numbers in the DR-TB, MDR-TB and XDR-TB groups of strains. The distribution of the number of FS mutations is shown. A Wilcoxon rank sum test was used to test the differences among the DR-TB, MDR-TB and XDR-TB groups. Figure S5. Protein-protein interaction for genes with frameshift indels in DR-TB strains. Figure S6. Protein-protein interaction for genes with frameshift indels in pan-susceptible strains comparing to DR-TB group. First, the FS mutations also existing in DR-TB strains were excluded. Then, the remained FS mutation in pan-susceptible strains were annotated onto the genes. The obtained gene list combined with known resistance-associated genes was inputted into STRING. The thicker the lines between two proteins, the stronger the relationship between them. The protein-protein interaction analysis took into consideration the following factors: the presence of fusion evidence, neighborhood evidence, co-occurrence evidence, experimental evidence, text mining evidence, database evidence and co-expression evidence. Figure S7. Validations using another data set. (a) Comparison of the FS indel numbers between drug-resistant and -sensitive strains. (b). Comparison of the IGR indel numbers between drug resistant and sensitive strains. (c). The Correlation between DNA repair mutation numbers and FS + IGR indel numbers. Each dot represents an MTB strain. (DOCX 10447 kb) [file 12864_2018_4734_MOESM1_ESM.docx]

**
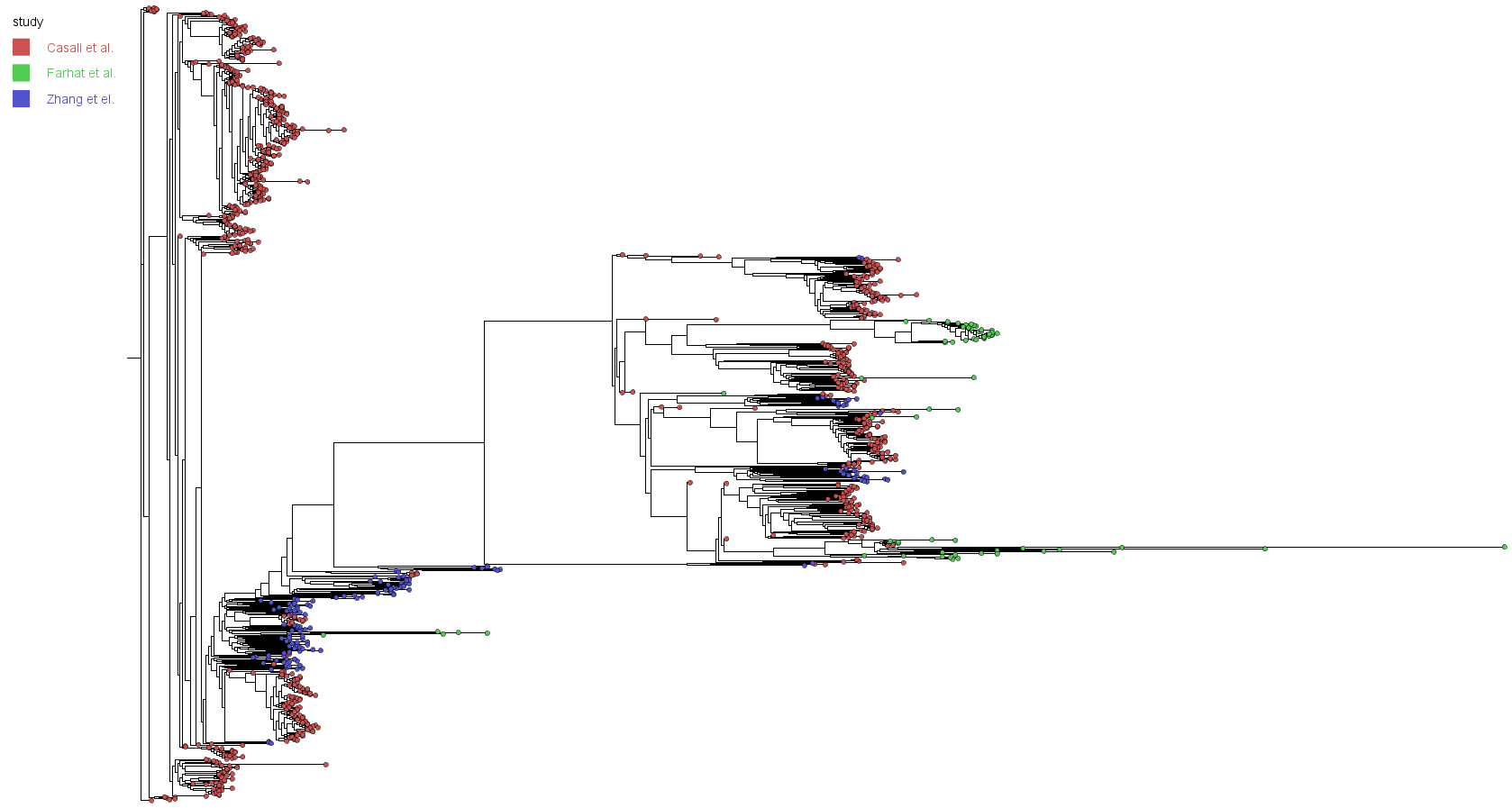
**

**Figure S1. The profile of 1110 isolates.**

**(a)** The distribution of isolates in three studies.


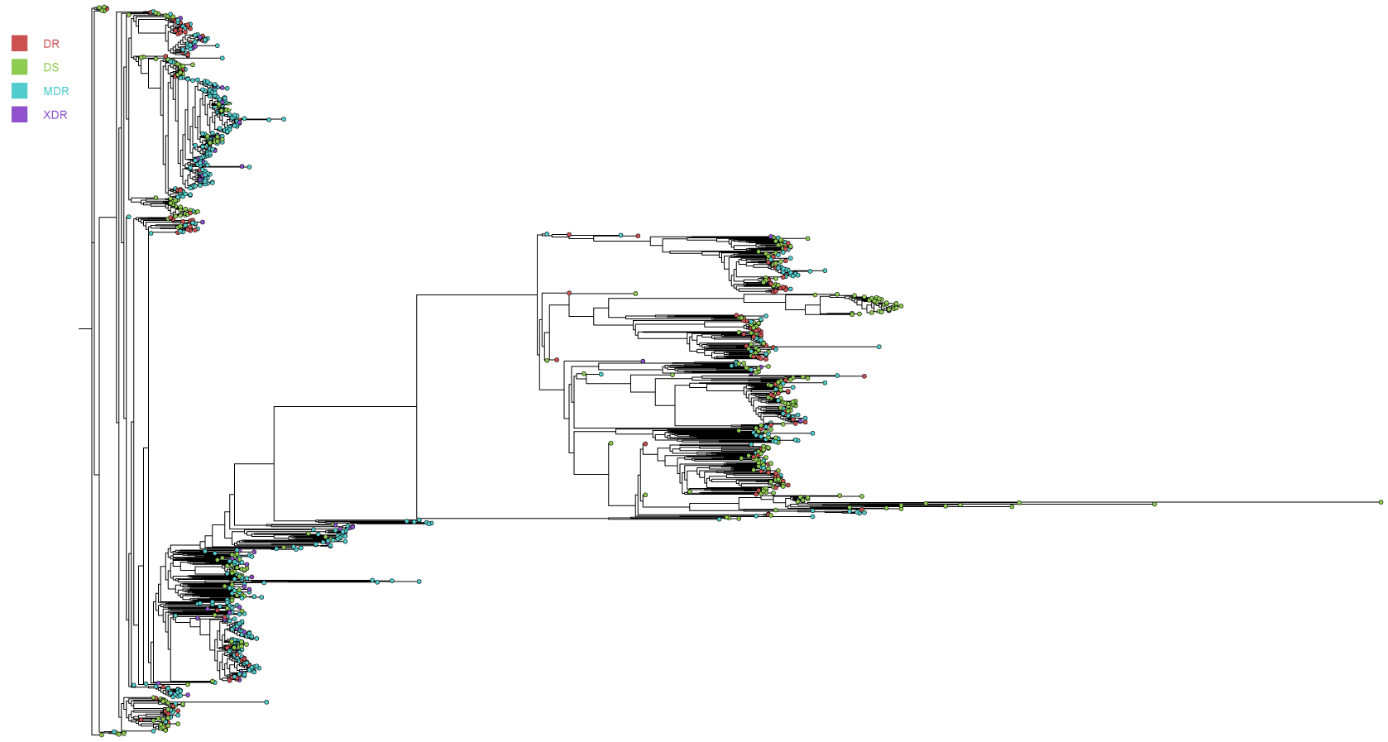


**Figure S1. The profile of 1110 isolates.**

**(b)** The drug profiles for the 1110 isolates.


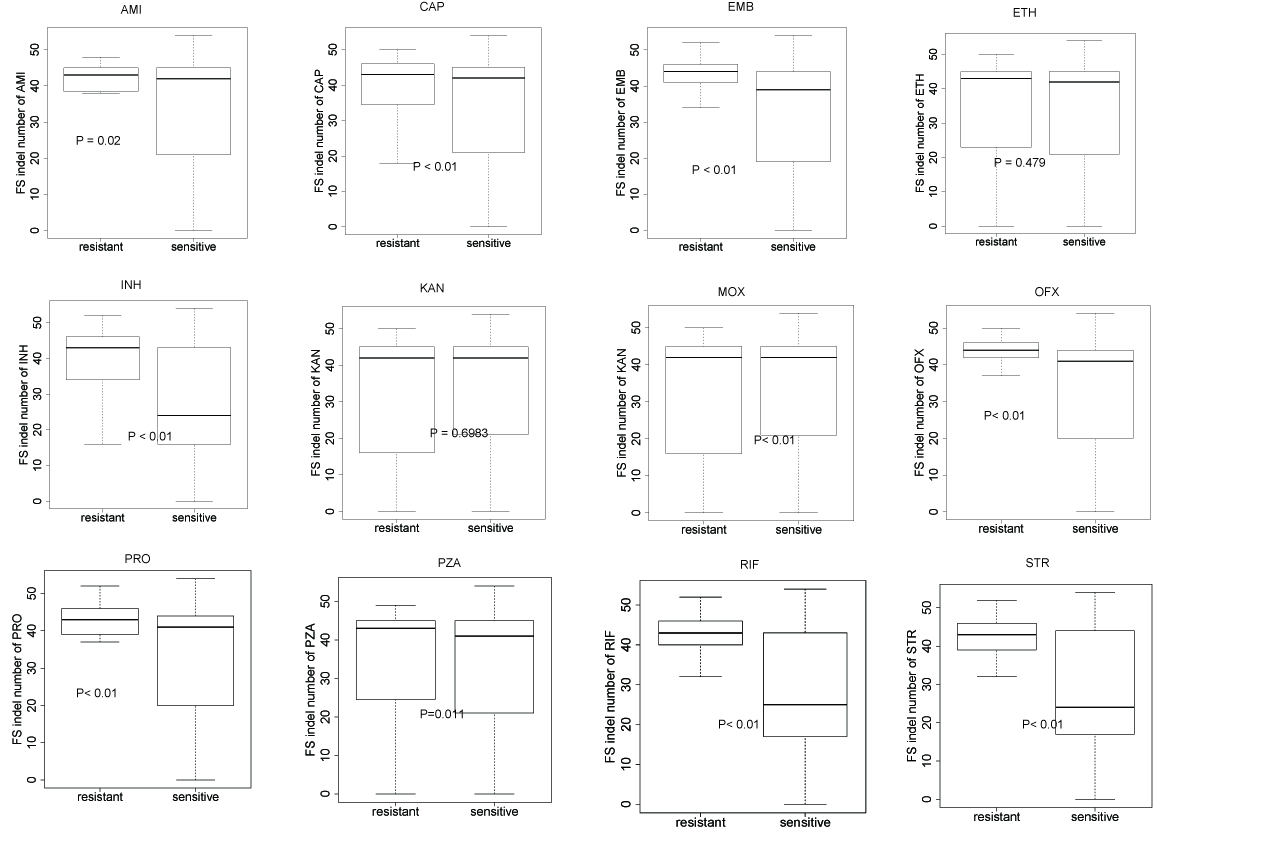

**Figure S2. Boxplot of the FS mutation numbers in the drug-resistant and -sensitive strains for each drug.** The p-values refer to corrected p-values.


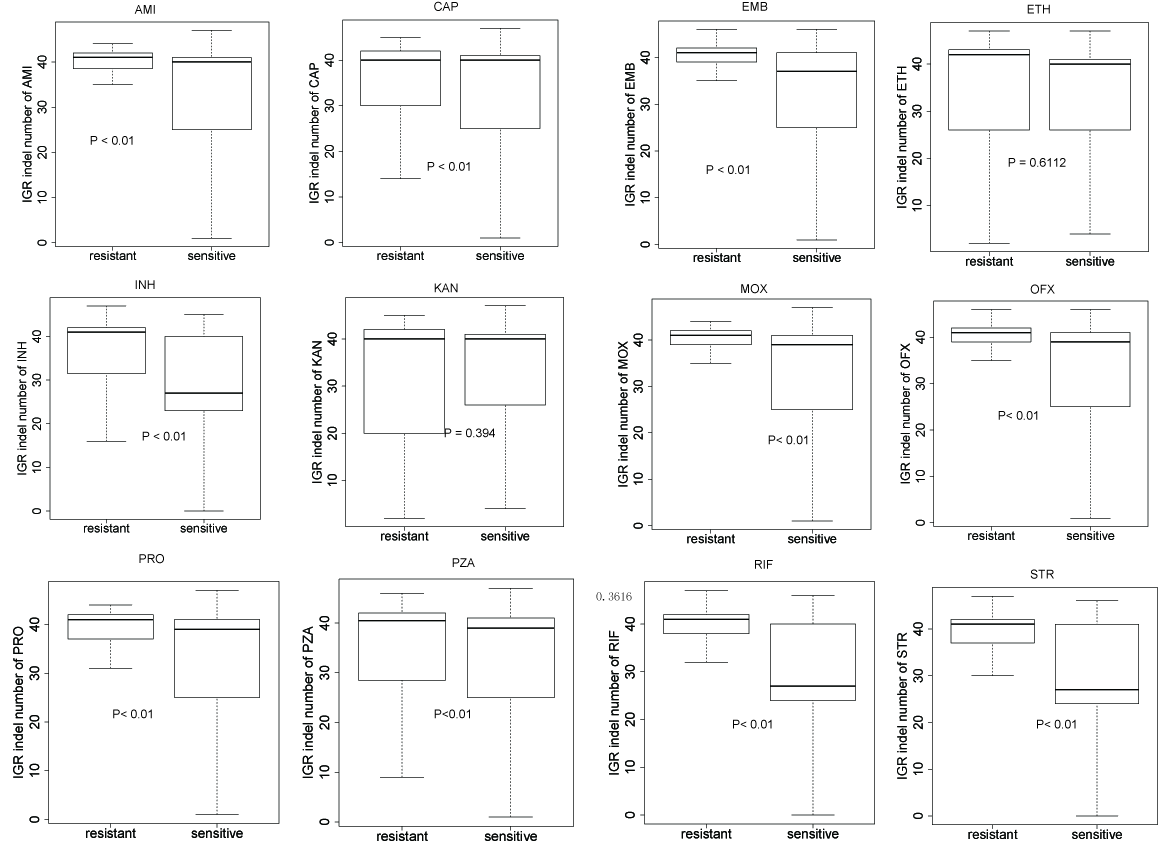


**Figure S3. Boxplot of the IGR indel numbers in the drug-resistant and -sensitive strains for each drug .** The p-values refer to corrected p-values.


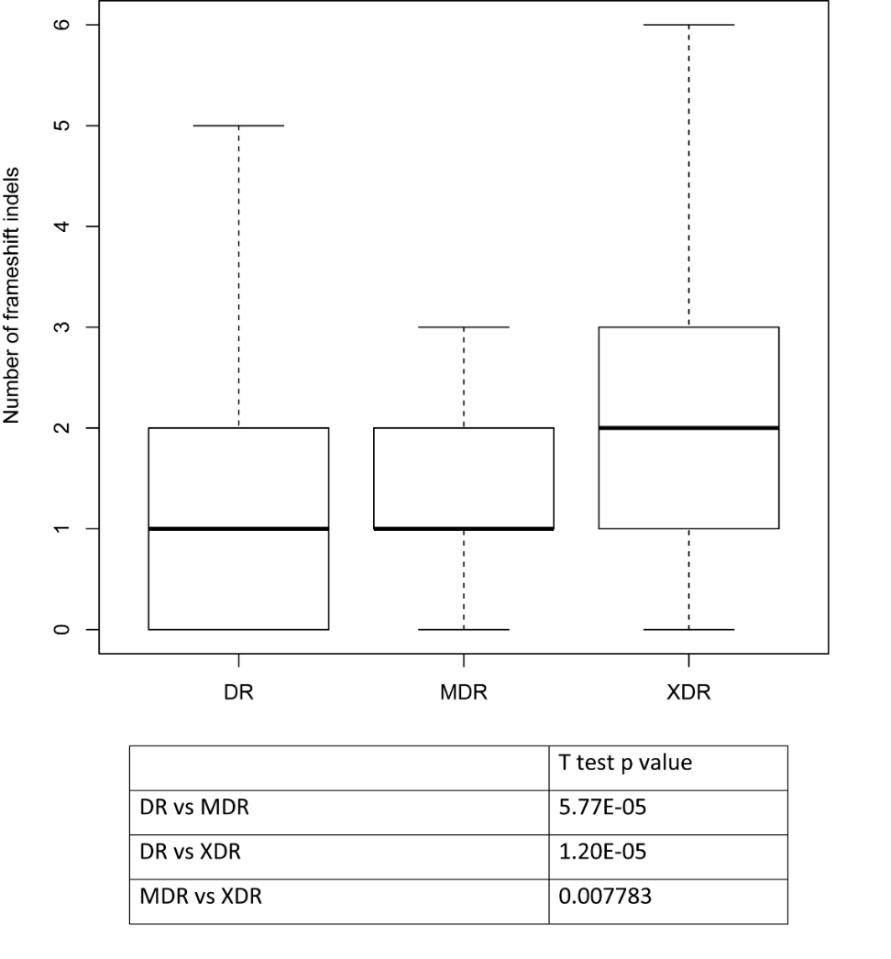


**Figure S4.** **Boxplot of the FS mutation numbers in the DR-TB, MDR-TB and XDR-TB groups of strains.** The distribution of the number of FS mutations is shown. A Wilcoxon rank sum test was used to test the differences among the DR-TB, MDR-TB and XDR-TB groups.


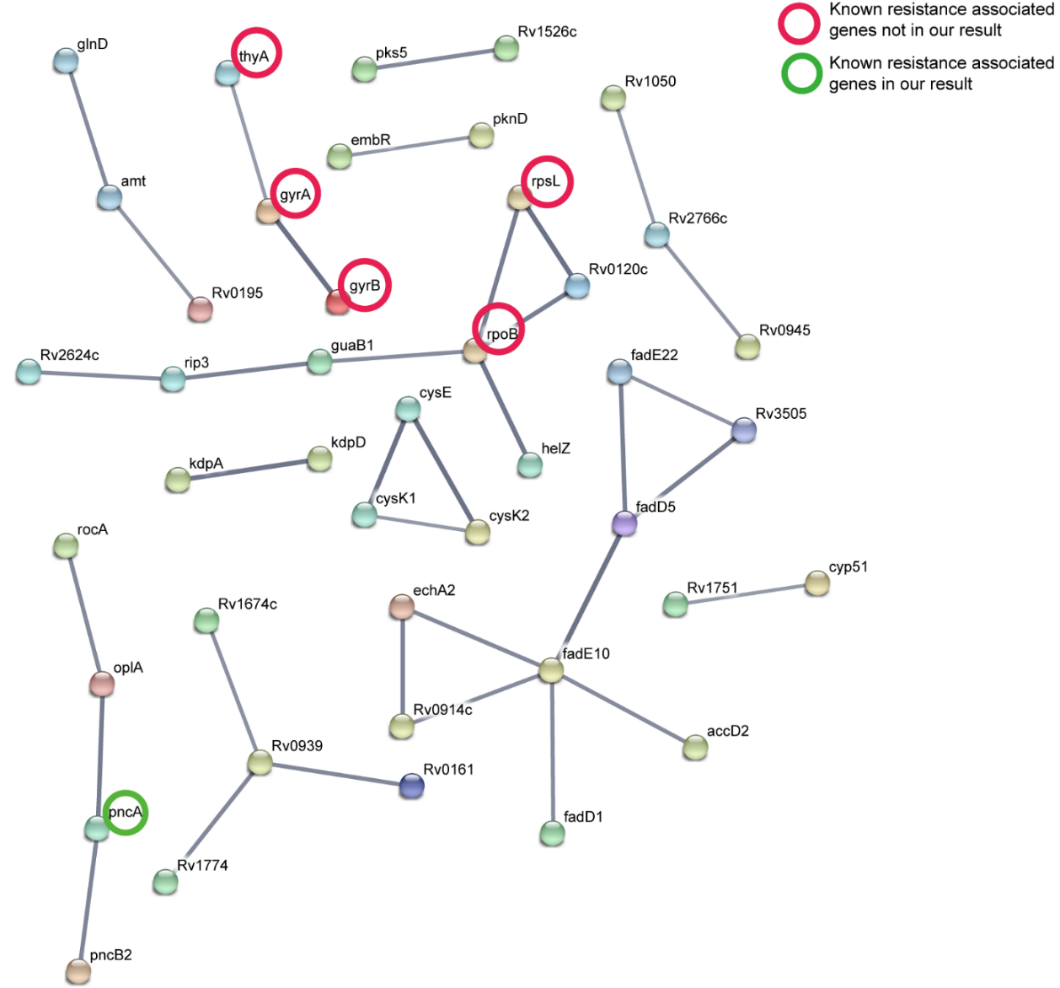


**Figure S5. Protein–protein interaction for genes with FS mutations in DR-TB strains.** First, the FS mutations also existing in pan-susceptible strains were excluded. Then, the remaining FS mutations in DR-TB strains were annotated onto the genes. The obtained gene list combined with known resistance-associated genes was inputted into STRING. The thicker the lines between two proteins, the stronger the relationship between them. The protein–protein interaction analysis took into consideration the following factors: the presence of fusion evidence, neighborhood evidence, co-occurrence evidence, experimental evidence, text-mining evidence, database evidence and co-expression evidence.


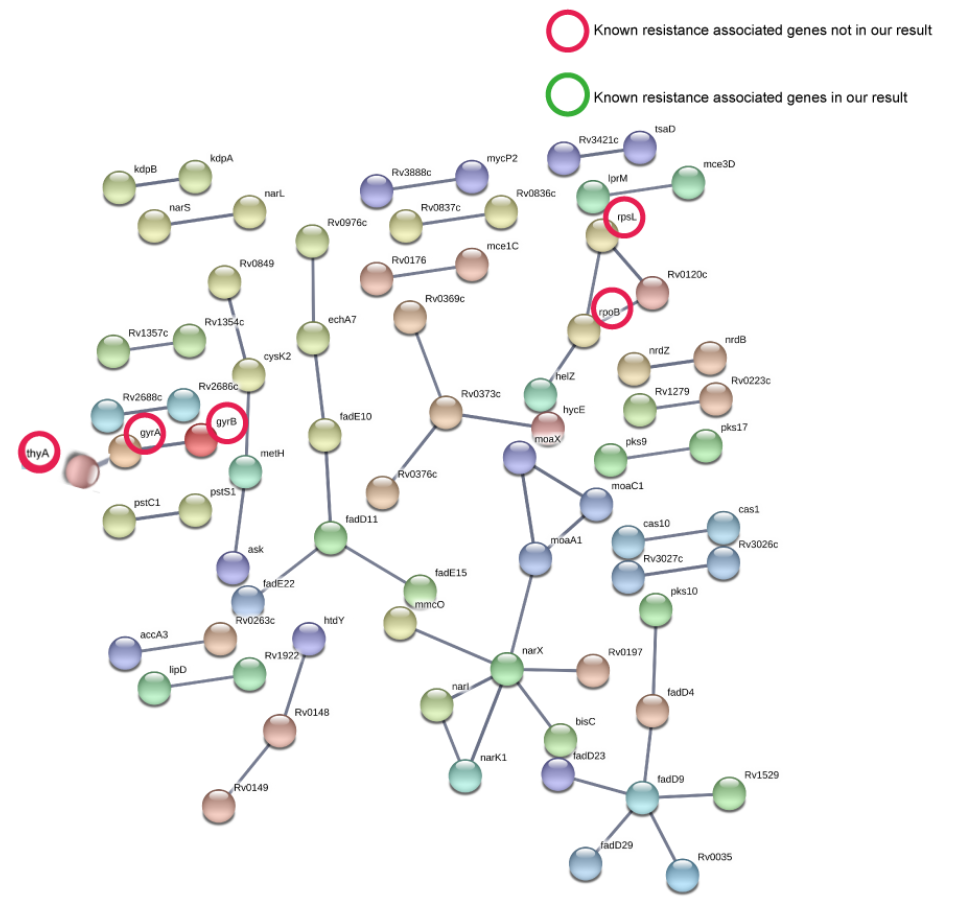


**Figure S6. Protein-protein interaction for genes with frameshift indels in pan-susceptible strains comparing to DR-TB group.** First, the FS mutations also existing in DR-TB strains were excluded. Then, the remained FS mutation in pan-susceptible strains were annotated onto the genes. The obtained gene list combined with known resistance-associated genes was inputted into STRING. The thicker the lines between two proteins, the stronger the relationship between them. The protein-protein interaction analysis took into consideration the following factors: the presence of fusion evidence, neighborhood evidence, co-occurrence evidence, experimental evidence, text mining evidence, database evidence and co-expression evidence.


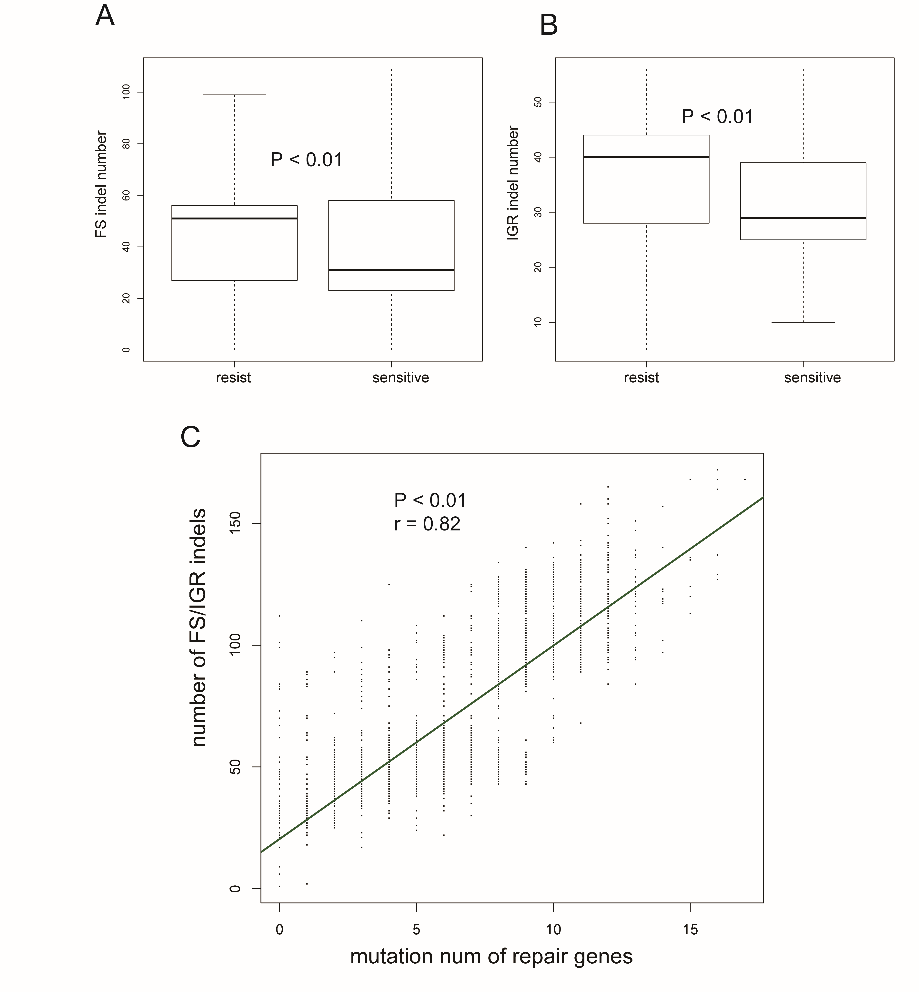


**Figure S7. Validations using another data set.** (a) Comparison of the FS indel numbers between drug-resistant and -sensitive strains. (b). Comparison of the IGR indel numbers between drug resistant and sensitive strains. (c). The Correlation between DNA repair mutation numbers and FS+IGR indel numbers. Each dot represents an MTB strain.
