# Supplementary material for: Whole genome sequencing data of 1110 Mycobacterium tuberculosis isolates identifies insertions and deletions associated with drug resistance
Source: BMC Genomics. 2018 May 16;19:365. doi: 10.1186/s12864-018-4734-6 (PMC5956929; doi:10.1186/s12864-018-4734-6)
Supplement: Supplementary file 2 — Table S1. Test for the difference in IGR indel incidence rate between resistant and sensitive strains for each drug among 1110 MTB strains. Table S2. Function enrichment for genes with at-least-one-drug-resistant strain-specific FS mutations in more than two resistant strains. Table S3. Function enrichment for genes with frameshift mutations in the DR-TB, MDR-TB and XDR-TB groups of strains. Table S4. The 20 region markers identified according to adjusted chi-square and Fisher’s exact p-values. Table S5. The 20 identified region markers and corresponding strain numbers. Table S6. Overview of functions for 20 region markers. Table S7. Description of the 20 region markers. Table S8. The distribution of region markers in function categories. Table S9. The 83 identified point markers of FS mutations and IGR indels, showing adjusted p-values. Table S10. The 83 identified point markers of FS mutations and IGR indels, showing strain numbers. Table S11. The identified point markers located in the IGRs expressing sRNA. Table S12. Overview of the functions of the 83 point markers. Table S13. Descriptions of the functions of the 83 point markers. Table S14. The 6 point markers out of the 83 point markers exclusively occurring in resistant strains. Table S15. P-values after logistic regression for the associations between the 20 region markers and drug resistance. Table S16. P-values after logistic regressions for the associations between the 83 point markers and drug resistance. Table S17. Effect of mutations on DNA repair genes. Table S18. Region markers in the validation set overlapping with the 20 region markers. Table S19. Point markers in the validation set overlapping with the 83 point markers. Table S20. Point markers in the 62 samples in which no known drug resistance associated SNPs were found. Table S21. The incidence rate for region markers. Table S22. The incidence rate for point markers. (DOCX 193 kb) [file 12864_2018_4734_MOESM2_ESM.docx]

# **SUPPLEMENTARY RESULTS**

## **The differences of distribution of FS mutations among groups of DR-TB, MDR-TB and XDR-TB**

To elaborate the difference of the incidence rate of FS mutations among three DR-TB, MDR-TB and XDR-TB groups, the incidence rate was calculated using the FS mutations and then the differences of the incidence rates among three groups were tested. Indels occurring in pan- susceptible strains were excluded in this analysis. The results showed significant differences were existing between MDR-TB and DR-TB (437/494 vs 134/182, p=4.15E-06, chi-square test), and between XDR-TB and DR-TB (58/67 vs 134/182, p=4.71E-02, chi-square test). To illustrate if the same situation was also existing in essential genes, only the genes that are essential for MTB growth[[1](#_ENREF_1)] was used to redo the above-mentioned calculations. As a result, significant results were still obtained. Significant difference was also found between DR-TB and XDR-TB (13/182 vs 15/67, p=0.006, Chi-square test). The same conclusion was found for the comparison of MDR-TB and XDR-TB (33/494 vs 15/67, p=4.49E-5, Chi-square test).  In addition, significant differences of FS mutation numbers were also found between each other for the three groups (Figure. S3).

## **Function enrichment for genes with FS mutations in DR-TB, MDR-TB and XDR-TB**

To check if the genes containing FS mutations exclusive in resistant strains can be enriched into pathways related to drug resistance or not, for DR-TB, MDR-TB and XDR-TB, we performed function enrichment analysis using DAVID [[2](#_ENREF_2)] and STRING.[[3](#_ENREF_3)] Using H37Rv as reference, indels occurring in pan-susceptible strains of each drug were excluded in this analysis. For genes with FS mutations in DR-TB, MDR-TB and XDR-TB strains. For the DR-TB group, the enriched pathway was “Cell membrane” and many genes including *guaB1* and *pncB2* were specifically interacted with known drug resistant genes (Additional file 2: Figure S5). The interacting network of genes containing indels specifically identified in pan-susceptible group comparing to DR-TB group was also presented as a reference (Additional file 2: Figure S6). For the MDR-TB group, the significant enriched function terms were “IPR016035:Acyl transferase/acyl hydrolase/lysophospholipase” and “IPR013154:Alcohol dehydrogenase GroES-like” (Additional file 1: Table S3), which were related to cell wall modules peptidoglycan-polymerizing PBP1b and lignin composition.[[4](#_ENREF_4), [5](#_ENREF_5)] In protein-protein network, *guaB1*, *mfd* and *nrdZ* were connected with known resistant genes. In addition, well known drug resistance associated genes were also detected in our results, including *pncA* and *thyA*. These results suggested that genes with FS mutations are potentially involved in the emergence of MTB drug resistance.

## **Assessment of the influence of population structure in identifying resistant markers**

The isolates were from three studies, Casali studies (879 strains from Samara of Russia), Zhang studies (161 strains from China), Farhat study (70 strains widely spreaded). To assess the influence of population structure to the identified markers, we also calculated the distribution of markers in different populations and found that all of the region markers were distributed in all three populations (Additional file1: Table Sx). For example, mce2A-mce2C showed incidence rate 0.758 of FS indels in resistant strains and 0.401 incidence rate in sensitive strains in Casali population. The ratios in Zhang studies and Farhat studies were 0.821 vs 0.614 in resistant and sensitive strains. The ratios in Farhat studies were 0.25 vs 0.037. The same analysis was also performed for point markers and the results was similar to region markers. These results suggested that the markers detected in this study can be applied to different populations (additional file1: Table Sx).

# **SUPPLEMENTARY TABLES**

## **Table S1. Test for the difference in IGR indel incidence rate between resistant and sensitive strains for each drug among 1110 MTB strains**

| Drug | Ratio of strains with FS mutation within resistant strains^1^ | Ratio of strains with FS mutation within sensitive strains^2^ | Adjusted Chi-square test p-value | | Adjusted Fisher’s exact test p-value |
| --- | --- | --- | --- | --- | --- |
| AMI | 0.23 | 0.36 | 0.071 | 0.092 | |
| CAP | 0.35 | 0.35 | 0.902 | 1.000 | |
| EMB | 0.32 | 0.37 | 0.103 | 0.133 | |
| **ETH** | **0.73** | **0.34** | **7.15E-07** | **3.02E-06** | |
| INH | 0.32 | 0.40 | 0.021 | 0.028 | |
| **KAN** | **0.64** | **0.35** | **0.006** | **0.009** | |
| **MOX** | **0.21** | **0.37** | **0.006** | **0.009** | |
| OFX | 0.37 | 0.35 | 0.710 | 0.944 | |
| **PRO** | **0.25** | **0.37** | **0.007** | **0.009** | |
| PZA | 0.29 | 0.37 | 0.076 | 0.095 | |
| RIF | 0.32 | 0.39 | 0.032 | 0.041 | |
| STR | 0.31 | 0.40 | 0.008 | 0.011 | |

Both the chi-square test and Fisher exact test were used to test the difference in the incidence rate between resistant and sensitive strains for each drug. The drugs with both chi-square and Fisher’s exact p-values lower than 0.01 were highlighted. FDR method was used to do multiple testing correction.

1 Incidence rate of FS mutations within resistant strains, which equals the number of resistant strains with FS mutations divided by the total number of resistant strains of a certain drug.

2 Incidence rate of FS mutations within sensitive strains, which equals the number of sensitive strains with FS mutations divided by the total number of sensitive strains of a certain drug.

## **Table S2. Function enrichment for genes with at-least-one-drug-resistant strain-specific FS mutations in more than two resistant samples**

| Term | Count | PValue | Genes | Benjamini |
| --- | --- | --- | --- | --- |
| IPR016035:Acyl transferase/acyl hydrolase/lysophospholipase | 10 | 1.59E-05 | PKS8, PKS7, RV3479, PKS4, PKS12, PKS5, RV2565, PKS1, RV3239C, RV3091 | 0.005877249 |
| IPR011032:GroES-like | 8 | 3.82E-04 | PKS8, ADHE1, PKS7, PKS4, PKS12, PKS5, PKS1, ADH | 0.034823568 |

The FS mutations existing in pan-susceptible strains were excluded. Then, the remaining FS mutations in at-least-one-drug-resistant strains were annotated onto the genes. The frequencies of a gene were defined as the number of MTB strains that contained the remaining FS mutations in this gene. The genes with a frequency higher than 2 were inputted into DAVID to check if any functions could be enriched.

## **Table S3. Function enrichment for genes with frameshift indels in the DR-TB, MDR-TB and XDR-TB groups of strains**

| DR | | | | |
| --- | --- | --- | --- | --- |
| Term | Count | P-Value | Genes | Benjamini |
| Cell membrane | 35 | 1.77E-04 | RV2688C, RV2625C, PKND, LPRA, RV3728, AMT, CTPV, RV2723, ARSB1, KDPD, RV3178, LPRI, RV3239C, PKNK, RV0805, KDPA, RV3479, PKS4, PLCB, PKS5, RV1490, CTPG, ETHA, CTPI, RV0194, MYCP4, RV1979C, LPQH, MMPL5, RV0090, ECCB4, CYP141, ECCB1, STP, MMPL1 | 0.019447235 |
| MDR | | | | |
| Term | Count | P-Value | Genes | Benjamini |
| IPR016035:Acyl transferase/acyl hydrolase/lysophospholipase | 12 | 4.84E-05 | PKS6, PKS8, PKS9, PKS7, RV3479, PKS4, PKS12, PKS5, RV2565, PKS3, PKS1, RV3239C | 0.00849293 |
| IPR013154:Alcohol dehydrogenase GroES-like | 11 | 5.94E-05 | PKS8, ADHE1, PKS7, PKS4, PKS12, PKS5, PKS3, PKS1, ADH, FADB4, RV0149 | 0.007820187 |

First, the FS mutations also existing in pan-susceptible strains were excluded. Then, the remaining FS mutation in drug resistant strains were annotated onto the genes. The obtained gene list was inputted into DAVID to check if any functions could be enriched.

**Table S4. The 20 region markers identified according to their adjusted chi-square and Fisher’s exact p-values**

| **Makers** | **INH** | **RIF** | **PZA** | **STR** | **EMB** | **OFX** | **MOX** | **ETH** | **KAN** | **AMI** | **CAP** | **PRO** |
| --- | --- | --- | --- | --- | --- | --- | --- | --- | --- | --- | --- | --- |
| Rv1995 | 0.00\|0.00 | 0.00\|0.00 | 0.00\|0.00 | 0.00\|0.00 | 0.00\|0.00 | 0.00\|0.00 | 0.00\|0.00 | -- | -- | 0.00\|0.00 | 0.04\|0.11 | 0.00\|0.00 |
| ethA | 0.00\|0.00 | 0.00\|0.00 | 0.02\|0.06 | 0.00\|0.00 | 0.00\|0.00 | 0.00\|0.01 | 0.01\|0.03 | 0.00\|0.051 | 0.00\|0.05 | -- | 0.00\|0.03 | 0.00\|0.00 |
| purM-Rv0810c | 0.47\|1 | 0.38\|1 | -- | -- | 0.64\|0.62 | 0.24\|0.38 | -- | 0.00\|0.02 | -- | -- | -- | -- |
| Rv2571c | 0.29\|0.49 | 0.10\|0.18 | 0.97\|1 | 0.41\|0.79 | 0.93\|1 | 0.00\|0.03 | 0.58\|0.49 | 0.14\|0.25 | 0.04\|0.21 | 0.32\|0.38 | 0.54\|0.48 | -- |
| PPE36-prcA | 0.01\|0.02 | 0.34\|0.56 | 0.78\|1 | 0.02\|0.02 | -- | 0.14\|0.26 | 0.25\|0.44 | -- | -- | -- | -- | 0.12\|0.19 |
| Rv3750c-serX | 0.00\|0.00 | 0.00\|0.00 | 0.04\|0.06 | 0.00\|0.00 | 0.00\|0.00 | 0.00\|0.00 | 0.00\|0.00 | -- | -- | 0.27\|0.38 | 0.09\|0.16 | 0.00\|0.00 |
| Rv0759c-Rv0760c | 0.00\|0.00 | 0.00\|0.01 | 0.20\|0.28 | 0.00\|0.00 | 0.00\|0.00 | 0.32\|0.42 | 0.05\|0.07 | -- | -- | 0.32\|0.41 | -- | 0.40\|0.44 |
| rseA-htrA | 0.00\|0.00 | 0.03\|0.04 | -- | 0.00\|0.00 | 0.43\|0.57 | 0.02\|0.00 | 0.16\|0.27 | -- | -- | -- | -- | 0.16\|0.25 |
| Rv0011c-Rv0012 | 0.47\|0.98 | 0.24\|0.55 | -- | -- | 0.22\|0.42 | 0.00\|0.00 | -- | 0.04\|0.19 | 0.00\|0.05 | -- | 0.00\|0.12 | -- |
| pncA | 0.00\|0.00 | 0.00\|0.00 | 0.00\|0.00 | 0.00\|0.00 | 0.00\|0.00 | 0.15\|0.26 | 0.50\|0.54 | 0.49\|0.37 | 0.00\|0.06 | 0.39\|0.33 | 0.16\|0.25 | 0.02\|0.05 |
| mce2A-mce2C | 0.00\|0.00 | 0.00\|0.00 | 0.00\|0.00 | 0.00\|0.00 | 0.00\|0.00 | 0.00\|0.00 | 0.00\|0.00 | 0.03\|0.09 | 0.80\|0.90 | 0.00\|0.00 | 0.00\|0.02 | 0.00\|0.00 |
| ndhA-Rv0393 | 0.00\|0.00 | 0.00\|0.00 | 0.00\|0.00 | 0.00\|0.00 | 0.00\|0.00 | 0.00\|0.00 | 0.00\|0.00 | -- | -- | 0.00\|0.00 | 0.50\|0.51 | 0.00\|0.00 |
| Rv1509 | 0.11\|0.11 | 0.02\|0.03 | 0.00\|0.00 | 0.01\|0.01 | 0.00\|0.00 | 0.00\|0.00 | 0.00\|0.00 | -- | -- | 0.28\|0.34 | 0.87\|0.84 | 0.00\|0.01 |
| kdpD | 0.00\|0.00 | 0.00\|0.00 | 0.00\|0.00 | 0.00\|0.00 | 0.00\|0.00 | 0.00\|0.00 | 0.00\|0.00 | -- | -- | 0.00\|0.00 | 0.01\|0.05 | 0.00\|0.00 |
| whiB6-Rv3863 | 0.00\|0.00 | 0.00\|0.01 | 0.97\|1 | 0.00\|0.00 | 0.22\|0.45 | 0.12\|0.26 | 0.20\|0.33 | -- | -- | 0.89\|1 | 0.24\|0.32 | 0.03\|0.06 |
| vapC2-Rv0302 | 0.16\|0.20 | 0.00\|0.00 | 0.15\|0.24 | 0.01\|0.01 | 0.00\|0.00 | 0.00\|0.00 | 0.01\|0.05 | 0.31\|0.28 | 0.71\|0.58 | 0.00\|0.02 | 0.20\|0.24 | 0.00\|0.00 |
| PE_PGRS55-PE_PGRS56 | 0.00\|0.00 | 0.00\|0.00 | 0.00\|0.00 | 0.00\|0.00 | 0.00\|0.00 | 0.00\|0.00 | 0.00\|0.00 | 0.07\|0.12 | -- | 0.01\|0.02 | 0.04\|0.08 | 0.00\|0.00 |
| Rv2081c | 0.00\|0.00 | 0.00\|0.00 | 0.42\|0.47 | 0.00\|0.00 | 0.00\|0.00 | 0.14\|0.26 | 0.02\|0.05 | -- | -- | 0.23\|0.26 | 0.69\|0.69 | -- |
| Rv3848-espR | 0.04\|0.03 | 0.00\|0.00 | 0.21\|0.28 | 0.01\|0.01 | 0.22\|0.45 | 0.17\|0.26 | 0.49\|0.48 | 0.87\|0.59 | 0.61\|0.54 | 0.13\|0.18 | 0.89\|0.74 | 0.45\|0.48 |
| ddn-Rv3548c | 0.00\|0.00 | 0.00\|0.00 | 0.00\|0.00 | 0.00\|0.00 | 0.00\|0.00 | 0.00\|0.00 | 0.00\|0.00 | 0.02\|0.09 | -- | 0.00\|0.00 | 0.00\|0.03 | 0.00\|0.00 |

First, the genomic regions were selected if having a significantly higher (p<0.05) density of FS or IGR indels than random distribution, whose p-value is not displayed here. Then, the selected regions were subjected to both a chi-square and Fisher’s exact test to analyze the differences in the incidence rate of FS mutations and IGR indels in the genomic regions (genes and IGRs) between resistant strains and sensitive strains for each drug. FDR method was used for multiple testing correction. The number before the “|” represents the adjusted p-value obtained in the chi-square test. The number after the “|” represents the adjusted p-value obtained in the Fisher’s exact test. If both of the adjusted p-values were less than 0.05 when a region marker was tested with a certain drug, then the region was reserved. Each p-value was considered to two decimal places. The symbol “--” meant that the incidence rate of FS or IGR indels in resistant strains to a particular drug was lower than the incidence rate in sensitive strains to the corresponding drug.

**Table S5. The 20 identified region markers and their corresponding strain numbers**

| **Markers^1^** | **INH** | **RIF** | **EMB** | **STR** | **CAP** | **KAN** | **PZA** | **ETH** | **AMI** | **OFX** | | | **PRO** | **MOX** |
| --- | --- | --- | --- | --- | --- | --- | --- | --- | --- | --- | --- | --- | --- | --- |
| ddn-Rv3548c | **516/683\|158/427** | **455/578\|219/532** | **282/346\|392/764** | **465/599\|209/511** | **71/95\|603/1015** | -- | **130/180\|544/930** | 35/45\|639/1065 | **51/64\|623/1046** | **153/174\|521/936** | | | **139/179\|535/931** | **93/105\|581/1005** |
| ethA | **42/683\|0/427** | **39/578\|3/532** | **27/346\|15/764** | **37/599\|5/511** | **9/95\|33/1015** | 5/28\|37/1082 | 13/180\|29/930 | 6/45\|36/1065 | **--** | **15/174\|27/936** | | | **20/179\|22/931** | **9/105\|33/1005** |
| kdpD | **98/683\|2/427** | **85/578\|15/532** | **59/346\|41/764** | **96/599\|4/511** | 16/95\|84/1015 | -- | **43/180\|57/930** | -- | **17/64\|83/1046** | **37/174\|63/936** | | | **39/179\|61/931** | **27/105\|73/1005** |
| mce2A-mce2C | **517/683\|161/427** | **457/578\|221/532** | **284/346\|394/764** | **466/599\|212/511** | **72/95\|606/1015** | 18/28\|660/1082 | **131/180\|547/930** | 35/45\|643/1065 | **51/64\|627/1046** | **153/174\|525/936** | | | **138/179\|540/931** | **93/105\|585/1005** |
| ndhA-Rv0393 | **342/683\|106/427** | **292/578\|156/532** | **189/346\|259/764** | **316/599\|132/511** | 42/95\|406/1015 | -- | **101/180\|347/930** | -- | **40/64\|408/1046** | **92/174\|356/936** | | | **108/179\|340/931** | **72/105\|376/1005** |
| PE_PGRS55-PE_PGRS56 | **482/683\|155/427** | **428/578\|209/532** | **265/346\|372/764** | **432/599\|205/511** | 65/95\|572/1015 | -- | **124/180\|513/930** | 32/45\|605/1065 | **47/64\|590/1046** | **142/174\|495/936** | | | **131/179\|506/931** | **88/105\|549/1005** |
| pncA | **28/683\|2/427** | **26/578\|4/532** | **19/346\|11/764** | **25/599\|5/511** | 5/95\|25/1015 | 4/28\|26/1082 | **19/180\|11/930** | 2/45\|28/1065 | 3/64\|27/1046 | 8/174\|22/936 | | | 10/179\|20/931 | 4/105\|26/1005 |
| PPE36-prcA | **679/683\|415/427** | 573/578\|521/532 | -- | **596/599\|498/511** | -- | -- | 178/180\|916/930 | -- | -- | 174/174\|920/936 | | | 179/179\|915/931 | 105/105\|989/1005 |
| purM-Rv0810c | 2/683\|0/427 | 2/578\|0/532 | 1/346\|1/764 | -- | -- | -- | -- | **2/45\|0/1065** | -- | 1/174\|1/936 | | | -- | -- |
| rseA-htrA | **669/683\|399/427** | **565/578\|503/532** | 336/346\|732/764 | **587/599\|481/511** | -- | -- | -- | -- | -- | **174/174\|894/936** | | | 176/179\|892/931 | 104/105\|964/1005 |
| Rv0011c-Rv0012 | 4/683\|0/427 | 4/578\|0/532 | 3/346\|1/764 | -- | 2/95\|2/1015 | 2/28\|2/1082 | -- | 1/45\|3/1065 | -- | **4/174\|0/936** | | | -- | -- |
| Rv0759c-Rv0760c | **616/683\|356/427** | **524/578\|448/532** | **320/346\|652/764** | **543/599\|429/511** | -- | -- | 164/180\|808/930 | -- | 59/64\|913/1046 | 157/174\|815/936 | | | 161/179\|811/931 | 99/105\|873/1005 |
| Rv1509 | 49/683\|16/427 | **45/578\|20/532** | **33/346\|32/764** | **47/599\|18/511** | 6/95\|59/1015 | -- | **23/180\|42/930** | -- | 6/64\|59/1046 | **25/174\|40/936** | | | **19/179\|46/931** | **18/105\|47/1005** |
| Rv1995 | **107/683\|6/427** | **92/578\|21/532** | **59/346\|54/764** | **106/599\|7/511** | 16/95\|97/1015 | -- | **47/180\|66/930** | -- | **17/64\|96/1046** | **36/174\|77/936** | | | **42/179\|71/931** | **29/105\|84/1005** |
| Rv2081c | **112/683\|34/427** | **106/578\|40/532** | **64/346\|82/764** | **104/599\|42/511** | 14/95\|132/1015 | -- | 28/180\|118/930 | -- | 12/64\|134/1046 | 30/174\|116/936 | | | -- | 22/105\|124/1005 |
| Rv2571c | 6/683\|0/427 | 6/578\|0/532 | 2/346\|4/764 | 5/599\|1/511 | 1/95\|5/1015 | 1/28\|5/1082 | 1/180\|5/930 | 1/45\|5/1065 | 1/64\|5/1046 | **4/174\|2/936** | | | -- | 1/105\|5/1005 |
| Rv3750c-serX | **650/683\|337/427** | **550/578\|437/532** | **331/346\|656/764** | **569/599\|418/511** | 90/95\|897/1015 | -- | 169/180\|818/930 | -- | 60/64\|927/1046 | **174/174\|813/936** | | | **177/179\|810/931** | **103/105\|884/1005** |
| Rv3848-espR | **19/683\|2/427** | **19/578\|2/532** | 10/346\|11/764 | **18/599\|3/511** | 2/95\|19/1015 | 1/28\|20/1082 | 6/180\|15/930 | 1/45\|20/1065 | 3/64\|18/1046 | 6/174\|15/936 | | | 5/179\|16/931 | 3/105\|18/1005 |
| vapC2-Rv0302 | 20/683\|4/427 | **22/578\|2/532** | **16/346\|8/764** | **20/599\|4/511** | 4/95\|20/1015 | 1/28\|23/1082 | 7/180\|17/930 | 2/45\|22/1065 | **5/64\|19/1046** | | **11/174\|13/936** | **13/179\|11/931** | | 6/105\|18/1005 |
| whiB6-Rv3863 | **674/683\|398/427** | **568/578\|504/532** | 339/346\|733/764 | **592/599\|480/511** | 94/95\|978/1015 | -- | 174/180\|898/930 | -- | 62/64\|1010/1046 | | 172/174\|900/936 | 178/179\|894/931 | | 104/105\|968/1005 |

First, genomic regions were selected if having a significantly higher density of FS or IGR indels than random distribution, whose p-values are not displayed here. Then the selected regions were subjected to both chi-square and Fisher’s exact test to analyze the differences in the incidence rates of FS mutations or IGR indels in genomic regions (genes and IGRs) between resistant strains and sensitive strains for each drug. FDR method was used for multiple testing correction. If both adjusted chi-square and Fisher’s exact p-values were less than 0.05 when a region marker was tested with a certain drug, then the region was reserved. Bold font indicates that the adjusted p-values for both the chi-square and fisher’s exact test were less than 0.05. The string in front of the “|” symbol represents the incidence rate of FS/IGR indels among the resistant strains of a drug, which was the number of resistant strains with FS mutations in genes or with IGR indels in IGRs for a certain drug divided by the number of resistant strains for a certain drug. The string behind the “|” symbol represents the number of sensitive strains with FS mutations in genes or with IGR indels in IGRs for a particular drug divided by the number of sensitive strains for a certain drug. The symbol “--” indicates that the incidence rate of FS or IGR indels in resistant strains of a drug was lower than the incidence rate in sensitive strains of the corresponding drug.

## **Table S6. Overview of the functions of region markers with significant relationships to drug resistance**

| **Functions** | **Number of markers** | **Markers** |
| --- | --- | --- |
| membrane | 13 | Rv3848-espR,mce2A-mce2C,ndhA-Rv0393,PE_PGRS55-PE_PGRS56,PPE36-prcA,Rv0011c-Rv0012,Rv1995,whiB6-Rv3863,ethA,kdpD,rseA-htrA,Rv2081c,Rv2571c |
| cell wall | 6 | Rv3848-espR,Rv3083,whiB6-Rv3863,ethA,PPE36-prcA,rseA-htrA |
| metabolism | 5 | Rv3083,Rv0759c-Rv0760c,ethA,purM-Rv0810c,pncA |
| growth | 4 | Rv3083,ndhA-Rv0393,PPE36-prcA,rseA-htrA |
| transcript regulation | 4 | Rv3848-espR,rseA-htrA,whiB6-Rv3863,vapC2-Rv0302 |
| efflux pump | 2 | Rv0194-Rv0195,Rv3064c-mmr |
| drug | 1 | ethA |
| transporter | 1 | mce2A-mce2C |
| antibiotics | 1 | purM-Rv0810c |

**Table S7. Description of the 20 region markers**

| **Makers** | **Location** | **Description** |
| --- | --- | --- |
| Rv3548c | coding region | Probable short-chain type dehydrogenase/reductase |
| ethA | coding region | monooxygenase EthA |
| kdpD | coding region | sensor protein KdpD |
| mce2A-mce2C | intergenic region | Mce family protein Mce2A/ Mce family protein Mce2C |
| ndhA-Rv0393 | intergenic region | NADH dehydrogenase NdhA/ hypothetical protein |
| PE_PGRS55-PE_PGRS56 | intergenic region | PE-PGRS family protein PE_PGRS55/ PE-PGRS family protein PE_PGRS56 |
| pncA | coding region | pyrazinamidase/nicotinamidase PncA |
| PPE36-prcA | intergenic region | PPE family protein PPE36/ proteasome subunit alpha |
| purM-Rv0810c | intergenic region | phosphoribosylformylglycinamidine cyclo-ligase PurM/ hypothetical protein |
| rseA-htrA | intergenic region | anti-sigma E factor RseA/ serine protease HtrA |
| Rv0011c-Rv0012 | intergenic region | cell division protein CrgA/ membrane protein |
| Rv0759c-Rv0760c | intergenic region | hypothetical protein/ hypothetical protein |
| Rv1509 | coding region | hypothetical protein |
| Rv1995 | coding region | hypothetical protein |
| Rv2081c | coding region | transmembrane protein |
| Rv2571c | coding region | transmembrane protein |
| Rv3750c-serX | intergenic region | excisionase/ tRNA |
| Rv3848-espR | intergenic region | transmembrane protein/ ESX-1 transcriptional regulator EspR |
| vapC2-Rv0302 | intergenic region | ribonuclease VapC2/ transcriptional regulator |
| whiB6-Rv3863 | intergenic region | transcriptional regulator WhiB6/ hypothetical protein |

**Table S8. The distribution of region markers in function categories**

| Category of genes | Observed ratio | Expected ratio | Fold enrich | Chi-square p-value |
| --- | --- | --- | --- | --- |
| Growth | 4/20 | ^1^780/4008 | 1.03 | 0.95 |
| Cell membrane | 13/20 | 1534/4008 | 1.70 | 0.014 |
| Cell wall | 6/20 | 629/4008 | 1.91 | 0.07 |

^1^ 780 means there are 780 genes functionally related growth in whole genome. 4008 means the total gene count in whole genome. 780/4008 means the ratio of genes in growth category in whole genome background.

## **Table S9. The 83 identified point markers of FS mutations and IGR indels, showing the adjusted p-value**

| Markers^1^ | Effect | INH | RIF | EMB | STR | CAP | KAN | PZA | ETH | AMI | OFX | PRO | MOX |
| --- | --- | --- | --- | --- | --- | --- | --- | --- | --- | --- | --- | --- | --- |
| Rv3517:3953533:T:TC | p.Gly37fs | 0.44\|1 | 0.35\|0.93 | 0.18\|0.38 | 0.37\|0.82 | 0.00\|0.13 | -- | 0.00\|0.00 | -- | 0.13\|0.27 | 0.00\|0.07 | 0.09\|0.22 | 0.00\|0.01 |
| argW-echA14:2794344:TG:T | IRG | 0.02\|0.01 | 0.01\|0.01 | -- | 0.00\|0.00 | -- | -- | 0.33\|0.37 | -- | -- | 0.66\|0.72 | 0.00\|0.00 | 0.63\|0.65 |
| pncA:2288850:A:ACC | p.Val131fs | 0.44\|1 | 0.37\|1 | 0.18\|0.38 | 0.37\|0.82 | 0.28\|0.36 | -- | 0.00\|0.00 | -- | 0.13\|0.27 | -- | 0.00\|0.05 | -- |
| Rv2955c:3308313:T:TG | p.His78fs | 0.00\|0.00 | 0.08\|0.09 | 0.01\|0.03 | 0.00\|0.00 | 0.00\|0.07 | -- | 0.00\|0.00 | -- | 0.38\|0.35 | 0.91\|0.76 | 0.06\|0.14 | 0.32\|0.30 |
| Rv0272c:328589:TG:T | p.Lys374fs | 0.00\|0.00 | 0.79\|1 | 0.30\|0.38 | 0.00\|0.00 | -- | -- | 0.00\|0.00 | -- | 0.02\|0.06 | -- | -- | 0.35\|0.36 |
| pckA-nadR:253609:CAGACGCATAAGCCCCCGCACGCACGGCGTGTCGAGGGCTTT:C | IRG | 0.44\|1 | 0.35\|0.93 | 0.50\|0.62 | 0.37\|0.82 | 0.00\|0.13 | -- | 0.00\|0.00 | -- | -- | 0.62\|0.51 | 0.09\|0.22 | 0.32\|0.36 |
| Rv3750c-serX:4198611:CG:C | IRG | 0.00\|0.00 | 0.00\|0.00 | 0.00\|0.00 | 0.00\|0.00 | 0.07\|0.13 | -- | 0.03\|0.07 | -- | 0.26\|0.36 | 0.00\|0.00 | 0.00\|0.00 | 0.00\|0.00 |
| PPE21-fadD11:1753519:G:GC | IRG | 0.00\|0.00 | 0.00\|0.00 | 0.18\|0.27 | 0.00\|0.00 | 0.77\|1 | -- | 0.33\|0.43 | -- | -- | 0.02\|0.01 | 0.00\|0.00 | 0.02\|0.02 |
| ctpI:125830:G:GA | p.Ser1571fs | 0.00\|0.00 | 0.02\|0.02 | 0.26\|0.38 | 0.00\|0.00 | 0.37\|0.56 | -- | 0.76\|1 | -- | 0.64\|1 | 0.02\|0.08 | 0.03\|0.03 | 0.10\|0.15 |
| Rv0759c-Rv0760c:854252:GCC:G | IRG | 0.76\|1 | 0.35\|1 | -- | -- | -- | 0.01\|0.05 | -- | 0.00\|0.00 | -- | 0.56\|0.52 | -- | -- |
| Rv0278c-PE_PGRS4:336557:C:CT | IRG | 0.00\|0.00 | 0.00\|0.00 | 0.00\|0.00 | 0.00\|0.00 | 0.02\|0.06 | 0.55\|0.63 | 0.03\|0.08 | 0.05\|0.06 | 0.01\|0.02 | 0.00\|0.00 | 0.00\|0.00 | 0.00\|0.00 |
| ndhA-Rv0393:472711:T:TTTGTGGGCC | IRG | 0.00\|0.00 | 0.00\|0.00 | 0.00\|0.00 | 0.00\|0.00 | 0.44\|0.43 | -- | 0.00\|0.00 | -- | 0.00\|0.00 | 0.00\|0.00 | 0.00\|0.00 | 0.00\|0.00 |
| Rv0197:234496:C:CGT | p.Pro756fs | 0.00\|0.00 | 0.35\|0.83 | -- | 0.00\|0.00 | -- | -- | -- | -- | -- | 0.02\|0.05 | 0.03\|0.03 | 0.09\|0.15 |
| mpt53-cdsA:3190145:TC:T | IRG | 0.00\|0.00 | 0.00\|0.00 | 0.00\|0.00 | 0.00\|0.00 | 0.14\|0.21 | -- | 0.01\|0.01 | -- | 0.02\|0.04 | 0.00\|0.00 | 0.00\|0.00 | 0.00\|0.00 |
| Rv0970-echA7:1081735:G:GT | IRG | 0.44\|1 | 0.28\|0.55 | 0.15\|0.26 | 0.28\|0.48 | 0.00\|0.03 | -- | 0.00\|0.01 | -- | 0.00\|0.00 | 0.02\|0.12 | 0.02\|0.08 | 0.00\|0.02 |
| PE9-Rv1088a:1215104:AT:A | IRG | 0.00\|0.00 | 0.00\|0.00 | 0.00\|0.00 | 0.00\|0.00 | 0.00\|0.02 | 0.31\|0.41 | 0.00\|0.00 | 0.04\|0.05 | 0.00\|0.00 | 0.00\|0.00 | 0.00\|0.00 | 0.00\|0.00 |
| PE_PGRS55-PE_PGRS56:3943744:CCGGCAACGG:C | IRG | 0.00\|0.00 | 0.00\|0.00 | 0.00\|0.00 | 0.00\|0.00 | 0.04\|0.12 | -- | 0.02\|0.03 | 0.04\|0.06 | 0.05\|0.09 | 0.00\|0.00 | 0.00\|0.00 | 0.00\|0.00 |
| Rv2294-Rv2295:2566766:C:CG | IRG | 0.00\|0.00 | 0.00\|0.00 | 0.00\|0.00 | 0.00\|0.00 | 0.00\|0.03 | 0.96\|1 | 0.00\|0.01 | 0.07\|0.10 | 0.00\|0.00 | 0.00\|0.00 | 0.00\|0.00 | 0.00\|0.00 |
| Rv0759c-Rv0760c:854252:GC:G | IRG | 0.03\|0.04 | 0.28\|0.33 | 0.05\|0.04 | 0.00\|0.00 | 0.91\|1 | -- | 0.01\|0.02 | -- | 0.05\|0.08 | 0.59\|0.61 | 0.03\|0.05 | 0.00\|0.00 |
| Rv3725:4170964:G:GA | p.Gly251fs | 0.00\|0.00 | 0.00\|0.00 | 0.00\|0.00 | 0.00\|0.00 | 0.00\|0.02 | 0.52\|0.63 | 0.00\|0.00 | 0.01\|0.05 | 0.00\|0.00 | 0.00\|0.00 | 0.00\|0.00 | 0.00\|0.00 |
| ppsA:3247865:GCAAA:G | p.Gln808fs | 0.00\|0.00 | 0.00\|0.00 | 0.10\|0.13 | 0.00\|0.00 | 0.61\|0.74 | -- | 0.03\|0.03 | -- | 0.76\|1 | 0.03\|0.12 | 0.00\|0.00 | 0.00\|0.00 |
| Rv0045c:49690:GCC:G | p.Gly83fs | 0.00\|0.00 | 0.00\|0.00 | 0.00\|0.00 | 0.00\|0.00 | 0.01\|0.06 | 0.98\|1 | 0.00\|0.01 | 0.08\|0.10 | 0.00\|0.00 | 0.00\|0.00 | 0.00\|0.00 | 0.00\|0.00 |
| Rv0194-Rv0195:230576:G:GT | IRG | 0.00\|0.00 | 0.00\|0.00 | 0.00\|0.00 | 0.00\|0.00 | 0.00\|0.02 | 0.35\|0.41 | 0.00\|0.01 | 0.01\|0.05 | 0.00\|0.00 | 0.00\|0.00 | 0.00\|0.00 | 0.00\|0.00 |
| nrdH-Rv3054c:3415180:ACACCTAGGGGGTGG:A | IRG | 0.00\|0.00 | 0.00\|0.00 | 0.06\|0.06 | 0.00\|0.00 | 0.47\|0.56 | -- | 0.24\|0.32 | -- | -- | 0.00\|0.00 | 0.00\|0.00 | 0.01\|0.00 |
| ephF:162151:GT:G | p.Phe129fs | 0.00\|0.00 | 0.00\|0.00 | 0.00\|0.00 | 0.00\|0.00 | 0.00\|0.02 | 0.52\|0.63 | 0.00\|0.01 | 0.02\|0.05 | 0.00\|0.00 | 0.00\|0.00 | 0.00\|0.00 | 0.00\|0.00 |
| Rv3684-proY:4126514:GGT:G | IRG | 0.44\|1 | 0.28\|0.55 | 0.15\|0.26 | 0.28\|0.48 | 0.00\|0.03 | -- | 0.00\|0.01 | -- | 0.00\|0.00 | 0.02\|0.12 | 0.02\|0.08 | 0.00\|0.02 |
| Rv1045-Rv1047:1168715:C:CT | IRG | 0.00\|0.00 | 0.12\|0.14 | 0.57\|0.57 | 0.00\|0.00 | 0.81\|1 | -- | 0.82\|1 | -- | -- | 0.02\|0.08 | 0.03\|0.05 | 0.11\|0.15 |
| Rv3202a-lipV:3580636:CT:C | IRG | 0.00\|0.00 | 0.03\|0.03 | 0.19\|0.38 | 0.00\|0.00 | 0.33\|0.56 | -- | 0.39\|0.53 | -- | 0.59\|1 | 0.02\|0.05 | 0.03\|0.02 | 0.09\|0.16 |
| Rv1861-adhA:2109523:C:CG | IRG | 0.00\|0.00 | 0.11\|0.14 | 0.22\|0.38 | 0.00\|0.00 | 0.51\|0.79 | -- | 0.67\|0.86 | -- | 0.45\|0.76 | 0.02\|0.01 | 0.01\|0.01 | 0.06\|0.11 |
| tgs3:3610391:A:AC | p.Ser267fs | 0.00\|0.00 | 0.00\|0.00 | 0.00\|0.00 | 0.00\|0.00 | 0.00\|0.03 | -- | 0.00\|0.00 | 0.02\|0.05 | 0.00\|0.00 | 0.00\|0.00 | 0.00\|0.00 | 0.00\|0.00 |
| Rv1179c-pks3:1313337:A:AG | IRG | 0.00\|0.00 | 0.35\|1 | -- | 0.00\|0.00 | -- | -- | 0.97\|1 | -- | -- | 0.13\|0.21 | 0.46\|0.57 | 0.57\|0.79 |
| Rv2975c:3331361:ACG:A | p.Arg84fs | 0.00\|0.00 | 0.00\|0.00 | 0.00\|0.00 | 0.00\|0.00 | 0.00\|0.02 | 0.98\|1 | 0.00\|0.00 | 0.08\|0.10 | 0.00\|0.00 | 0.00\|0.00 | 0.00\|0.00 | 0.00\|0.00 |
| Rv3830c:4305063:G:GA | p.Ser208fs | 0.00\|0.00 | 0.00\|0.00 | 0.00\|0.00 | 0.00\|0.00 | 0.00\|0.02 | 0.98\|1 | 0.00\|0.00 | 0.04\|0.05 | 0.00\|0.00 | 0.00\|0.00 | 0.00\|0.00 | 0.00\|0.00 |
| Rv1714:1942396:GC:G | p.Lys183fs | 0.44\|1 | 0.28\|0.55 | 0.15\|0.26 | 0.28\|0.48 | 0.00\|0.03 | -- | 0.00\|0.01 | -- | 0.00\|0.00 | 0.02\|0.12 | 0.02\|0.08 | 0.00\|0.02 |
| dxs2-Rv3382c:3794867:C:CCA | IRG | 0.00\|0.00 | 0.00\|0.00 | 0.00\|0.00 | 0.00\|0.00 | 0.01\|0.06 | -- | 0.00\|0.01 | 0.14\|0.18 | 0.00\|0.00 | 0.00\|0.00 | 0.00\|0.00 | 0.00\|0.00 |
| plsB1:1756358:CG:C | p.Gly306fs | 0.00\|0.00 | 0.00\|0.00 | 0.00\|0.00 | 0.00\|0.00 | 0.00\|0.02 | 0.50\|0.63 | 0.00\|0.00 | 0.01\|0.05 | 0.00\|0.00 | 0.00\|0.00 | 0.00\|0.00 | 0.00\|0.00 |
| serB1-mmpS2:596701:CAGG:C | IRG | 0.37\|0.50 | 0.11\|0.16 | 0.18\|0.38 | 0.12\|0.14 | 0.00\|0.07 | -- | 0.00\|0.00 | -- | 0.38\|0.39 | -- | 0.00\|0.05 | 0.67\|0.52 |
| Rv0739:830868:G:GGC | p.Glu9fs | 0.00\|0.00 | 0.00\|0.00 | 0.00\|0.00 | 0.00\|0.00 | 0.00\|0.02 | 0.71\|0.75 | 0.12\|0.23 | 0.02\|0.05 | 0.03\|0.06 | 0.00\|0.00 | 0.00\|0.00 | 0.00\|0.00 |
| Rv1145:1273250:G:GA | p.Leu277fs | 0.00\|0.00 | 0.00\|0.00 | 0.00\|0.00 | 0.00\|0.00 | 0.00\|0.02 | 0.98\|1 | 0.00\|0.00 | 0.02\|0.05 | 0.00\|0.00 | 0.00\|0.00 | 0.00\|0.00 | 0.00\|0.00 |
| ctpE-Rv0909:1014300:G:GT | IRG | 0.37\|0.50 | 0.11\|0.16 | 0.18\|0.38 | 0.12\|0.14 | 0.00\|0.07 | -- | 0.00\|0.00 | -- | 0.38\|0.39 | -- | 0.00\|0.05 | 0.67\|0.52 |
| whiB6-Rv3863:4338595:GC:G | IRG | 0.00\|0.00 | 0.00\|0.00 | 0.18\|0.38 | 0.00\|0.00 | 0.15\|0.22 | 0.93\|1 | -- | -- | 0.74\|1 | 0.23\|0.33 | 0.02\|0.02 | 0.11\|0.17 |
| rplM-esxT:3862472:GA:G | IRG | 0.00\|0.00 | 0.00\|0.00 | 0.31\|0.38 | 0.00\|0.00 | -- | -- | 0.48\|0.58 | -- | -- | 0.02\|0.00 | 0.03\|0.03 | 0.05\|0.07 |
| sigG:214096:C:CA | p.Val16fs | 0.37\|0.50 | 0.11\|0.16 | 0.18\|0.38 | 0.12\|0.14 | 0.00\|0.07 | -- | 0.00\|0.00 | -- | 0.38\|0.39 | -- | 0.00\|0.05 | 0.67\|0.52 |
| ltp1:3100154:A:AC | p.Ser6fs | 0.00\|0.00 | 0.00\|0.00 | 0.00\|0.00 | 0.00\|0.00 | 0.27\|0.29 | -- | 0.74\|0.70 | -- | 0.53\|0.52 | 0.00\|0.00 | 0.00\|0.00 | 0.00\|0.00 |
| Rv1928c:2181054:A:ATC | p.Ile55fs | 0.37\|0.50 | 0.11\|0.16 | 0.18\|0.38 | 0.12\|0.14 | 0.00\|0.07 | -- | 0.00\|0.00 | -- | 0.38\|0.39 | -- | 0.00\|0.05 | 0.67\|0.52 |
| Rv1042c-Rv1043c:1165521:T:TA | IRG | 0.00\|0.00 | 0.30\|0.37 | 0.75\|0.91 | 0.00\|0.00 | 0.77\|1 | -- | 0.54\|0.63 | -- | -- | 0.02\|0.04 | 0.00\|0.00 | 0.02\|0.02 |
| Rv3402c-Rv3403c:3822042:A:AC | IRG | 0.44\|1 | 0.28\|0.55 | 0.15\|0.26 | 0.28\|0.48 | 0.00\|0.03 | -- | 0.00\|0.01 | -- | 0.00\|0.00 | 0.02\|0.12 | 0.02\|0.08 | 0.00\|0.02 |
| mazE3-Rv1106c:1232563:CCTTACGT:C | IRG | 0.37\|0.50 | 0.11\|0.16 | 0.18\|0.38 | 0.12\|0.14 | 0.00\|0.07 | -- | 0.00\|0.00 | -- | 0.38\|0.39 | -- | 0.00\|0.05 | 0.67\|0.52 |
| Rv2264c:2536628:G:GA | p.Pro575fs | 0.00\|0.00 | 0.00\|0.00 | 0.00\|0.00 | 0.00\|0.00 | 0.00\|0.02 | 0.69\|0.75 | 0.00\|0.00 | 0.51\|0.54 | 0.00\|0.00 | 0.00\|0.00 | 0.00\|0.00 | 0.00\|0.00 |
| ppsA:3247864:C:CTAGG | p.Gln808fs | 0.00\|0.00 | 0.00\|0.00 | 0.06\|0.06 | 0.00\|0.00 | 0.69\|0.87 | -- | 0.03\|0.05 | -- | 0.82\|1 | 0.02\|0.03 | 0.00\|0.00 | 0.00\|0.00 |
| sigM:4400660:AC:A | p.Arg160fs | 0.12\|0.15 | 0.64\|1 | 0.18\|0.38 | 0.00\|0.00 | 0.67\|0.83 | -- | 0.02\|0.01 | -- | 0.22\|0.32 | 0.15\|0.21 | 0.00\|0.00 | 0.01\|0.01 |
| PE12-fbiC:1302917:G:GA | IRG | 0.44\|1 | 0.28\|0.55 | 0.15\|0.26 | 0.28\|0.48 | 0.00\|0.03 | -- | 0.00\|0.01 | -- | 0.00\|0.00 | 0.02\|0.12 | 0.02\|0.08 | 0.00\|0.02 |
| rseA-htrA:1365837:C:CGG | IRG | 0.00\|0.00 | 0.00\|0.00 | 0.00\|0.00 | 0.00\|0.00 | 0.00\|0.03 | -- | 0.00\|0.00 | 0.18\|0.23 | 0.00\|0.00 | 0.00\|0.00 | 0.00\|0.00 | 0.00\|0.00 |
| vapB18:2867880:TA:T | p.Leu33fs | 0.00\|0.00 | 0.00\|0.00 | 0.00\|0.00 | 0.00\|0.00 | 0.00\|0.02 | 0.50\|0.63 | 0.00\|0.00 | 0.01\|0.05 | 0.00\|0.00 | 0.00\|0.00 | 0.00\|0.00 | 0.00\|0.00 |
| Rv3897c:4383144:C:CCGGGG | p.Gly166fs | 0.00\|0.00 | 0.13\|0.17 | 0.57\|0.64 | 0.00\|0.00 | -- | -- | 0.77\|1 | -- | -- | 0.02\|0.00 | 0.01\|0.00 | 0.05\|0.07 |
| Rv1225c:1368322:C:CG | p.Gly134fs | 0.00\|0.00 | 0.00\|0.00 | 0.00\|0.00 | 0.00\|0.00 | 0.00\|0.02 | 0.50\|0.63 | 0.00\|0.00 | 0.02\|0.05 | 0.00\|0.00 | 0.00\|0.00 | 0.00\|0.00 | 0.00\|0.00 |
| pks15:3296371:G:GCCGCGGC | p.Arg491fs | 0.00\|0.00 | 0.00\|0.00 | 0.00\|0.00 | 0.00\|0.00 | 0.00\|0.02 | 0.95\|1 | 0.00\|0.00 | 0.13\|0.14 | 0.00\|0.00 | 0.00\|0.00 | 0.00\|0.00 | 0.00\|0.00 |
| lipX-mshB:1300271:A:AT | IRG | 0.44\|1 | 0.28\|0.55 | 0.15\|0.26 | 0.28\|0.48 | 0.00\|0.03 | -- | 0.00\|0.01 | -- | 0.00\|0.00 | 0.02\|0.12 | 0.02\|0.08 | 0.00\|0.02 |
| Rv1775:2009290:GC:G | p.Gln42fs | 0.00\|0.00 | 0.00\|0.00 | 0.00\|0.00 | 0.00\|0.00 | 0.00\|0.02 | 0.48\|0.53 | 0.00\|0.00 | 0.13\|0.18 | 0.00\|0.00 | 0.00\|0.00 | 0.00\|0.00 | 0.00\|0.00 |
| Rv2251:2525722:CG:C | p.Glu55fs | 0.00\|0.00 | 0.01\|0.02 | 0.18\|0.38 | 0.00\|0.00 | 0.83\|1 | -- | 0.86\|1 | -- | -- | 0.02\|0.01 | 0.01\|0.00 | 0.06\|0.07 |
| Rv2081c:2338194:A:AC | p.Val105fs | 0.00\|0.00 | 0.00\|0.00 | 0.00\|0.00 | 0.00\|0.00 | 0.44\|0.46 | -- | 0.15\|0.23 | -- | 0.12\|0.16 | 0.02\|0.12 | -- | 0.00\|0.02 |
| Rv3847:4322039:AC:A | p.Ala169fs | 0.00\|0.00 | 0.00\|0.00 | 0.00\|0.00 | 0.00\|0.00 | 0.00\|0.02 | 0.31\|0.41 | 0.00\|0.01 | 0.04\|0.05 | 0.00\|0.00 | 0.00\|0.00 | 0.00\|0.00 | 0.00\|0.00 |
| Rv0108c-PE_PGRS1:131174:T:TG | IRG | 0.00\|0.00 | 0.05\|0.08 | 0.52\|0.56 | 0.00\|0.00 | 0.15\|0.32 | -- | 0.63\|0.82 | -- | 0.74\|1 | 0.04\|0.11 | 0.04\|0.07 | 0.13\|0.23 |
| Rv0420c-Rv0421c:507028:GC:G | IRG | 0.00\|0.00 | 0.00\|0.00 | 0.00\|0.00 | 0.00\|0.00 | 0.00\|0.02 | 0.74\|0.89 | 0.00\|0.00 | 0.04\|0.05 | 0.00\|0.00 | 0.00\|0.00 | 0.00\|0.00 | 0.00\|0.00 |
| mce2A-mce2C:688792:T:TG | IRG | 0.00\|0.00 | 0.00\|0.00 | 0.00\|0.00 | 0.00\|0.00 | 0.00\|0.02 | 0.77\|0.89 | 0.00\|0.00 | 0.02\|0.05 | 0.00\|0.00 | 0.00\|0.00 | 0.00\|0.00 | 0.00\|0.00 |
| esxU:3863170:A:AGCATC | p.Leu32fs | 0.37\|0.50 | 0.11\|0.16 | 0.18\|0.38 | 0.12\|0.14 | 0.00\|0.07 | -- | 0.00\|0.00 | -- | 0.38\|0.39 | -- | 0.00\|0.05 | 0.67\|0.52 |
| dosT:2273733:TC:T | p.Glu259fs | 0.00\|0.00 | 0.00\|0.00 | 0.00\|0.00 | 0.00\|0.00 | 0.00\|0.02 | 0.50\|0.63 | 0.00\|0.00 | 0.02\|0.05 | 0.00\|0.00 | 0.00\|0.00 | 0.00\|0.00 | 0.00\|0.00 |
| prfB-fprA:3473996:G:GA | IRG | 0.00\|0.00 | 0.00\|0.00 | 0.00\|0.00 | 0.00\|0.00 | 0.02\|0.06 | -- | 0.12\|0.23 | -- | 0.02\|0.02 | 0.00\|0.00 | 0.00\|0.00 | 0.00\|0.00 |
| Rv3747-Rv3748:4197138:C:CT | IRG | 0.00\|0.00 | 0.00\|0.00 | 0.00\|0.00 | 0.00\|0.00 | 0.02\|0.06 | 0.49\|0.63 | 0.01\|0.01 | -- | 0.01\|0.01 | 0.00\|0.00 | 0.00\|0.00 | 0.00\|0.00 |
| Rv2426c-proA:2724180:TCACGATCGGGTCTCCTCTAG:T | IRG | 0.00\|0.00 | 0.00\|0.00 | 0.00\|0.00 | 0.00\|0.00 | 0.09\|0.13 | -- | 0.00\|0.00 | 0.18\|0.23 | 0.05\|0.09 | 0.00\|0.00 | 0.00\|0.00 | 0.00\|0.00 |
| pks6:485810:CA:C | p.Asn28fs | 0.00\|0.00 | 0.00\|0.00 | 0.00\|0.00 | 0.00\|0.00 | 0.00\|0.02 | 0.50\|0.63 | 0.00\|0.00 | 0.02\|0.05 | 0.00\|0.00 | 0.00\|0.00 | 0.00\|0.00 | 0.00\|0.00 |
| mce1R:194305:C:CGG | p.Gly171fs | 0.00\|0.00 | 0.00\|0.00 | 0.00\|0.00 | 0.00\|0.00 | 0.00\|0.03 | -- | 0.00\|0.00 | 0.04\|0.05 | 0.00\|0.00 | 0.00\|0.00 | 0.00\|0.00 | 0.00\|0.00 |
| Rv0658c:754108:AC:A | p.Val101fs | 0.44\|1 | 0.28\|0.55 | 0.15\|0.26 | 0.28\|0.48 | 0.00\|0.03 | -- | 0.00\|0.01 | -- | 0.00\|0.00 | 0.02\|0.12 | 0.02\|0.08 | 0.00\|0.02 |
| plcA-PPE38:2632341:C:CA | IRG | 0.00\|0.00 | 0.00\|0.00 | 0.00\|0.00 | 0.00\|0.00 | 0.00\|0.02 | 0.31\|0.41 | 0.02\|0.03 | 0.04\|0.05 | 0.00\|0.00 | 0.00\|0.00 | 0.00\|0.00 | 0.00\|0.00 |
| Rv2084:2342649:A:AGGCGTACACAC | p.Ser282fs | 0.00\|0.00 | 0.00\|0.00 | 0.00\|0.00 | 0.00\|0.00 | 0.01\|0.05 | 0.82\|0.89 | 0.01\|0.02 | 0.05\|0.06 | 0.00\|0.01 | 0.00\|0.00 | 0.00\|0.00 | 0.00\|0.00 |
| Rv2293c:2564368:G:GC | p.Ala222fs | 0.00\|0.00 | 0.00\|0.00 | 0.00\|0.00 | 0.00\|0.00 | 0.02\|0.06 | -- | 0.00\|0.00 | 0.60\|0.64 | 0.00\|0.00 | 0.00\|0.00 | 0.00\|0.00 | 0.00\|0.00 |
| Rv0922-Rv0923c:1029386:G:GCGC | IRG | 0.44\|1 | 0.35\|1 | 0.16\|0.26 | 0.46\|1 | -- | -- | -- | 0.00\|0.00 | -- | 0.02\|0.16 | -- | -- |
| moeY:1523702:CG:C | p.Val111fs | 0.00\|0.00 | 0.00\|0.00 | 0.00\|0.00 | 0.00\|0.00 | -- | -- | -- | -- | 0.81\|0.78 | 0.02\|0.08 | 0.00\|0.00 | 0.00\|0.00 |
| Rv2264c:2536625:C:CG | p.Gly576fs | 0.44\|1 | 0.35\|0.81 | 0.29\|0.38 | 0.60\|1 | -- | -- | -- | 0.00\|0.00 | -- | 0.03\|0.16 | -- | -- |
| Rv0976c-PE_PGRS16:1090188:A:AG | IRG | 0.00\|0.00 | 0.00\|0.00 | 0.00\|0.00 | 0.00\|0.00 | 0.00\|0.02 | 0.77\|0.89 | 0.00\|0.00 | 0.09\|0.11 | 0.00\|0.00 | 0.00\|0.00 | 0.00\|0.00 | 0.00\|0.00 |
| vapC2-Rv0302:364498:TG:T | IRG | 0.37\|0.51 | 0.00\|0.00 | 0.00\|0.01 | 0.04\|0.04 | 0.49\|0.49 | -- | 0.09\|0.23 | 0.94\|0.62 | 0.00\|0.02 | 0.00\|0.01 | 0.00\|0.00 | 0.01\|0.04 |
| Rv1045:1168009:GC:G | p.Glu115fs | 0.00\|0.00 | 0.00\|0.00 | 0.00\|0.00 | 0.00\|0.00 | 0.00\|0.02 | 0.69\|0.75 | 0.00\|0.00 | 0.34\|0.36 | 0.00\|0.00 | 0.00\|0.00 | 0.00\|0.00 | 0.00\|0.00 |
| aceAa:2161343:G:GT | p.Thr296fs | 0.00\|0.00 | 0.00\|0.00 | 0.00\|0.00 | 0.00\|0.00 | 0.06\|0.13 | -- | 0.04\|0.09 | -- | 0.04\|0.07 | 0.00\|0.00 | 0.00\|0.00 | 0.00\|0.00 |

In the second column, the string with the prefix “p.” represents amino acid changes. For example, P.Glu115fs indicates a frameshift mutation from the 115th amino acid (Glu) of the protein. “IGR” indicates that the indel existed in an intergenic region. FDR method was used for multiple testing correction. The number in front of the “|” was the adjusted p-value determined by the chi-square test. The number behind the “|” was the adjusted p-value determined by Fisher’s exact test. P-values were obtained by testing the incidence rate of FS mutations or IGR indels among the resistant strains of a drug versus the incidence rate of FS mutations or IGR indels among the sensitive strains of the corresponding drug. P-values were considered to two decimal places. If both the chi-square and Fisher’s exact p-values were less than 0.01 when a marker was tested with a certain drug, then the marker was reserved. The symbol “--” indicates that the incidence rate of FS or IGR indels in resistant strains of a drug was lower than the incidence rate in sensitive strains of the corresponding drug.

1The format of the string in 1st column was region_name:postion_in_H37Rv_genome:reference_allele:alternative_allele.

## **Table S10. The 83 identified point markers of FS mutations and IGR indels, showing the corresponding strain numbers**

| **Markers^1^** | **Effect** | **INH** | **RIF** | **EMB** | **STR** | **CAP** | **KAN** | **PZA** | **ETH** | **AMI** | **OFX** | **PRO** | **MOX** |
| --- | --- | --- | --- | --- | --- | --- | --- | --- | --- | --- | --- | --- | --- |
| argW-echA14:2794344:TG:T^1^ | IRG | 15/683\|0/427 | 14/578\|1/532 | -- | **15/599\|0/511** | -- | -- | 4/180\|11/930 | -- | -- | 3/174\|12/936 | **8/179\|7/931** | 2/105\|13/1005 |
| pckA-nadR:253609:CAGACGCATAAGCCCCCGCACGCACGGCGTGTCGAGGGCTTT:C | IRG | 4/683\|0/427 | 4/578\|0/532 | 2/346\|2/764 | 4/599\|0/511 | 2/95\|2/1015 | -- | **4/180\|0/930** | -- | -- | 1/174\|3/936 | 2/179\|2/931 | 1/105\|3/1005 |
| Rv3517:3953533:T:TC | p.Gly37fs | 4/683\|0/427 | 4/578\|0/532 | 3/346\|1/764 | 4/599\|0/511 | 2/95\|2/1015 | -- | **4/180\|0/930** | -- | 1/64\|3/1046 | 3/174\|1/936 | 2/179\|2/931 | 3/105\|1/1005 |
| pncA:2288850:A:ACC | p.Val131fs | 4/683\|0/427 | 3/578\|1/532 | 3/346\|1/764 | 4/599\|0/511 | 1/95\|3/1015 | -- | **4/180\|0/930** | -- | 1/64\|3/1046 | -- | 3/179\|1/931 | -- |
| Rv0272c:328589:TG:T | p.Lys374fs | **52/683\|0/427** | 28/578\|24/532 | 20/346\|32/764 | 52/599\|0/511 | -- | -- | **18/180\|34/930** | -- | 7/64\|45/1046 | -- | -- | 7/105\|45/1005 |
| Rv2955c:3308313:T:TG | p.His78fs | **18/683\|0/427** | 15/578\|3/532 | 12/346\|6/764 | **18/599\|0/511** | 5/95\|13/1015 | -- | **9/180\|9/930** | -- | 2/64\|16/1046 | 3/174\|15/936 | 6/179\|12/931 | 3/105\|15/1005 |
| tgs3:3610391:A:AC | p.Ser267fs | **515/683\|159/427** | **455/578\|219/532** | **283/346\|391/764** | **464/599\|210/511** | 71/95\|603/1015 | -- | **129/180\|545/930** | 35/45\|639/1065 | **51/64\|623/1046** | **153/174\|521/936** | **138/179\|536/931** | **93/105\|581/1005** |
| Rv1179c-pks3:1313337:A:AG | IRG | **668/683\|398/427** | 560/578\|506/532 | -- | **587/599\|479/511** | -- | -- | 173/180\|893/930 | -- | -- | 171/174\|895/936 | 174/179\|892/931 | 102/105\|964/1005 |
| Rv2294-Rv2295:2566766:C:CG | IRG | **510/683\|157/427** | **451/578\|216/532** | **278/346\|389/764** | **459/599\|208/511** | 70/95\|597/1015 | 17/28\|650/1082 | 127/180\|540/930 | 33/45\|634/1065 | **51/64\|616/1046** | **151/174\|516/936** | **139/179\|528/931** | **92/105\|575/1005** |
| sigM:4400660:AC:A | p.Arg160fs | 656/683\|395/427 | 549/578\|502/532 | 334/346\|717/764 | **581/599\|470/511** | 91/95\|960/1015 | -- | 178/180\|873/930 | -- | 63/64\|988/1046 | 169/174\|882/936 | **179/179\|872/931** | 105/105\|946/1005 |
| Rv0970-echA7:1081735:G:GT | IRG | 5/683\|0/427 | 5/578\|0/532 | 4/346\|1/764 | 5/599\|0/511 | 3/95\|2/1015 | -- | 4/180\|1/930 | -- | **3/64\|2/1046** | 3/174\|2/936 | 3/179\|2/931 | 3/105\|2/1005 |
| Rv1714:1942396:GC:G | p.Lys183fs | 5/683\|0/427 | 5/578\|0/532 | 4/346\|1/764 | 5/599\|0/511 | 3/95\|2/1015 | -- | 4/180\|1/930 | -- | **3/64\|2/1046** | 3/174\|2/936 | 3/179\|2/931 | 3/105\|2/1005 |
| Rv0759c-Rv0760c:854252:GC:G | IRG | 569/683\|325/427 | 480/578\|414/532 | 296/346\|598/764 | **510/599\|384/511** | 77/95\|817/1015 | -- | 159/180\|735/930 | -- | 58/64\|836/1046 | 143/174\|751/936 | 156/179\|738/931 | **98/105\|796/1005** |
| PE12-fbiC:1302917:G:GA | IRG | 5/683\|0/427 | 5/578\|0/532 | 4/346\|1/764 | 5/599\|0/511 | 3/95\|2/1015 | -- | 4/180\|1/930 | -- | **3/64\|2/1046** | 3/174\|2/936 | 3/179\|2/931 | 3/105\|2/1005 |
| Rv2251:2525722:CG:C | p.Glu55fs | **674/683\|396/427** | 567/578\|503/532 | 338/346\|732/764 | **591/599\|479/511** | 92/95\|978/1015 | -- | 174/180\|896/930 | -- | -- | 174/174\|896/936 | 179/179\|891/931 | 105/105\|965/1005 |
| PE9-Rv1088a:1215104:AT:A | IRG | **516/683\|159/427** | **456/578\|219/532** | **284/346\|391/764** | **465/599\|210/511** | 73/95\|602/1015 | 20/28\|655/1082 | **129/180\|546/930** | 34/45\|641/1065 | **52/64\|623/1046** | **152/174\|523/936** | **138/179\|537/931** | **92/105\|583/1005** |
| sigG:214096:C:CA | p.Val16fs | 7/683\|0/427 | 7/578\|0/532 | 4/346\|3/764 | 7/599\|0/511 | 3/95\|4/1015 | -- | **6/180\|1/930** | -- | 1/64\|6/1046 | -- | 4/179\|3/931 | 1/105\|6/1005 |
| Rv2293c:2564368:G:GC | p.Ala222fs | **493/683\|156/427** | **433/578\|216/532** | **269/346\|380/764** | **448/599\|201/511** | 67/95\|582/1015 | -- | **128/180\|521/930** | 28/45\|621/1065 | **51/64\|598/1046** | **145/174\|504/936** | **138/179\|511/931** | **93/105\|556/1005** |
| moeY:1523702:CG:C | p.Val111fs | **188/683\|23/427** | **181/578\|30/532** | **101/346\|110/764** | **157/599\|54/511** | -- | -- | -- | -- | 13/64\|198/1046 | 45/174\|166/936 | **59/179\|152/931** | **40/105\|171/1005** |
| dxs2-Rv3382c:3794867:C:CCA | IRG | **512/683\|159/427** | **452/578\|219/532** | **281/346\|390/764** | **461/599\|210/511** | 69/95\|602/1015 | -- | 127/180\|544/930 | 32/45\|639/1065 | **51/64\|620/1046** | **152/174\|519/936** | **138/179\|533/931** | **93/105\|578/1005** |
| Rv0739:830868:G:GGC | p.Glu9fs | **467/683\|159/427** | **429/578\|197/532** | **262/346\|364/764** | **416/599\|210/511** | 68/95\|558/1015 | 17/28\|609/1082 | 112/180\|514/930 | 33/45\|593/1065 | 45/64\|581/1046 | **147/174\|479/936** | **132/179\|494/931** | **86/105\|540/1005** |
| plsB1:1756358:CG:C | p.Gly306fs | **518/683\|157/427** | **458/578\|217/532** | **285/346\|390/764** | **467/599\|208/511** | 72/95\|603/1015 | 19/28\|656/1082 | **130/180\|545/930** | 36/45\|639/1065 | **52/64\|623/1046** | **153/174\|522/936** | **139/179\|536/931** | **93/105\|582/1005** |
| aceAa:2161343:G:GT | p.Thr296fs | **594/683\|275/427** | **505/578\|364/532** | **309/346\|560/764** | **535/599\|334/511** | 82/95\|787/1015 | -- | 152/180\|717/930 | -- | 57/64\|812/1046 | **164/174\|705/936** | **162/179\|707/931** | **101/105\|768/1005** |
| Rv1928c:2181054:A:ATC | p.Ile55fs | 7/683\|0/427 | 7/578\|0/532 | 4/346\|3/764 | 7/599\|0/511 | 3/95\|4/1015 | -- | **6/180\|1/930** | -- | 1/64\|6/1046 | -- | 4/179\|3/931 | 1/105\|6/1005 |
| pks6:485810:CA:C | p.Asn28fs | **516/683\|158/427** | **456/578\|218/532** | **282/346\|392/764** | **465/599\|209/511** | 72/95\|602/1015 | 19/28\|655/1082 | **129/180\|545/930** | 35/45\|639/1065 | **52/64\|622/1046** | **151/174\|523/936** | **139/179\|535/931** | **92/105\|582/1005** |
| mpt53-cdsA:3190145:TC:T | IRG | **639/683\|331/427** | **539/578\|431/532** | **325/346\|645/764** | **559/599\|411/511** | 88/95\|882/1015 | -- | 169/180\|801/930 | -- | 62/64\|908/1046 | **173/174\|797/936** | **177/179\|793/931** | **103/105\|867/1005** |
| Rv3747-Rv3748:4197138:C:CT | IRG | **639/683\|337/427** | **540/578\|436/532** | **324/346\|652/764** | **564/599\|412/511** | 91/95\|885/1015 | 26/28\|950/1082 | 170/180\|806/930 | -- | 63/64\|913/1046 | **171/174\|805/936** | **177/179\|799/931** | **103/105\|873/1005** |
| ppsA:3247864:C:CTAGG | p.Gln808fs | **655/683\|359/427** | **550/578\|464/532** | 328/346\|686/764 | **575/599\|439/511** | 88/95\|926/1015 | -- | 173/180\|841/930 | -- | 59/64\|955/1046 | 168/174\|846/936 | **177/179\|837/931** | **104/105\|910/1005** |
| mazE3-Rv1106c:1232563:CCTTACGT:C | IRG | 7/683\|0/427 | 7/578\|0/532 | 4/346\|3/764 | 7/599\|0/511 | 3/95\|4/1015 | -- | **6/180\|1/930** | -- | 1/64\|6/1046 | -- | 4/179\|3/931 | 1/105\|6/1005 |
| pks15:3296371:G:GCCGCGGC | p.Arg491fs | **506/683\|159/427** | **446/578\|219/532** | **278/346\|387/764** | **455/599\|210/511** | 71/95\|594/1015 | 17/28\|648/1082 | **129/180\|536/930** | 32/45\|633/1065 | **51/64\|614/1046** | **151/174\|514/936** | **137/179\|528/931** | **91/105\|574/1005** |
| Rv1775:2009290:GC:G | p.Gln42fs | **510/683\|157/427** | **450/578\|217/532** | **280/346\|387/764** | **460/599\|207/511** | 72/95\|595/1015 | 19/28\|648/1082 | **130/180\|537/930** | 32/45\|635/1065 | **52/64\|615/1046** | **148/174\|519/936** | **139/179\|528/931** | **92/105\|575/1005** |
| Rv3202a-lipV:3580636:CT:C | IRG | **677/683\|402/427** | 570/578\|509/532 | 340/346\|739/764 | **594/599\|485/511** | 94/95\|985/1015 | -- | 177/180\|902/930 | -- | 63/64\|1016/1046 | 174/174\|905/936 | 179/179\|900/931 | 105/105\|974/1005 |
| Rv1225c:1368322:C:CG | p.Gly134fs | **517/683\|155/427** | **457/578\|215/532** | **285/346\|387/764** | **466/599\|206/511** | 72/95\|600/1015 | 19/28\|653/1082 | **129/180\|543/930** | 35/45\|637/1065 | **51/64\|621/1046** | **153/174\|519/936** | **139/179\|533/931** | **93/105\|579/1005** |
| Rv3750c-serX:4198611:CG:C | IRG | **650/683\|337/427** | **550/578\|437/532** | **331/346\|656/764** | **569/599\|418/511** | 90/95\|897/1015 | -- | 169/180\|818/930 | -- | 60/64\|927/1046 | **174/174\|813/936** | **177/179\|810/931** | **103/105\|884/1005** |
| PE_PGRS55-PE_PGRS56:3943744:CCGGCAACGG:C | IRG | **440/683\|131/427** | **391/578\|180/532** | **239/346\|332/764** | **393/599\|178/511** | 59/95\|512/1015 | -- | 109/180\|462/930 | 30/45\|541/1065 | 41/64\|530/1046 | **126/174\|445/936** | **116/179\|455/931** | **75/105\|496/1005** |
| Rv3830c:4305063:G:GA | p.Ser208fs | **516/683\|156/427** | **456/578\|216/532** | **283/346\|389/764** | **465/599\|207/511** | 71/95\|601/1015 | 17/28\|655/1082 | **129/180\|543/930** | 34/45\|638/1065 | **51/64\|621/1046** | **153/174\|519/936** | **139/179\|533/931** | **93/105\|579/1005** |
| ppsA:3247865:GCAAA:G | p.Gln808fs | **652/683\|358/427** | **547/578\|463/532** | 326/346\|684/764 | **572/599\|438/511** | 88/95\|922/1015 | -- | 173/180\|837/930 | -- | 59/64\|951/1046 | **166/174\|844/936** | **177/179\|833/931** | **104/105\|906/1005** |
| Rv0420c-Rv0421c:507028:GC:G | IRG | **516/683\|156/427** | **456/578\|216/532** | **284/346\|388/764** | **465/599\|207/511** | 72/95\|600/1015 | 18/28\|654/1082 | **129/180\|543/930** | 34/45\|638/1065 | 52/64\|620/1046 | **153/174\|519/936** | **138/179\|534/931** | **93/105\|579/1005** |
| Rv0658c:754108:AC:A | p.Val101fs | 5/683\|0/427 | 5/578\|0/532 | 4/346\|1/764 | 5/599\|0/511 | 3/95\|2/1015 | -- | 4/180\|1/930 | -- | **3/64\|2/1046** | 3/174\|2/936 | 3/179\|2/931 | 3/105\|2/1005 |
| ltp1:3100154:A:AC | p.Ser6fs | **172/683\|2/427** | **169/578\|5/532** | **92/346\|82/764** | **145/599\|29/511** | 19/95\|155/1015 | -- | 30/180\|144/930 | -- | 12/64\|162/1046 | **43/174\|131/936** | **53/179\|121/931** | **35/105\|139/1005** |
| Rv3897c:4383144:C:CCGGGG | p.Gly166fs | **669/683\|399/427** | 564/578\|504/532 | 335/346\|733/764 | **589/599\|479/511** | -- | -- | 174/180\|894/930 | -- | -- | 174/174\|894/936 | 179/179\|889/931 | 105/105\|963/1005 |
| mce1R:194305:C:CGG | p.Gly171fs | **515/683\|161/427** | **455/578\|221/532** | **281/346\|395/764** | **464/599\|212/511** | 71/95\|605/1015 | -- | **130/180\|546/930** | 34/45\|642/1065 | **51/64\|625/1046** | **153/174\|523/936** | **139/179\|537/931** | **93/105\|583/1005** |
| ctpE-Rv0909:1014300:G:GT | IRG | 7/683\|0/427 | 7/578\|0/532 | 4/346\|3/764 | 7/599\|0/511 | 3/95\|4/1015 | -- | **6/180\|1/930** | -- | 1/64\|6/1046 | -- | 4/179\|3/931 | 1/105\|6/1005 |
| Rv1045-Rv1047:1168715:C:CT | IRG | **676/683\|406/427** | 570/578\|512/532 | 339/346\|743/764 | **594/599\|488/511** | 93/95\|989/1015 | -- | 176/180\|906/930 | -- | -- | 174/174\|908/936 | 179/179\|903/931 | 105/105\|977/1005 |
| Rv2081c:2338194:A:AC | p.Val105fs | **110/683\|23/427** | **103/578\|30/532** | **63/346\|70/764** | **101/599\|32/511** | 14/95\|119/1015 | -- | 28/180\|105/930 | -- | 12/64\|121/1046 | 30/174\|103/936 | -- | 22/105\|111/1005 |
| Rv0194-Rv0195:230576:G:GT | IRG | **518/683\|167/427** | **458/578\|227/532** | **284/346\|401/764** | **467/599\|218/511** | 73/95\|612/1015 | 20/28\|665/1082 | 130/180\|555/930 | 36/45\|649/1065 | **52/64\|633/1046** | **152/174\|533/936** | **139/179\|546/931** | **92/105\|593/1005** |
| nrdH-Rv3054c:3415180:ACACCTAGGGGGTGG:A | IRG | **671/683\|371/427** | **564/578\|478/532** | 335/346\|707/764 | **589/599\|453/511** | 91/95\|951/1015 | -- | 173/180\|869/930 | -- | -- | **174/174\|868/936** | **179/179\|863/931** | 105/105\|937/1005 |
| mce2A-mce2C:688792:T:TG | IRG | **517/683\|160/427** | **457/578\|220/532** | **284/346\|393/764** | **466/599\|211/511** | 72/95\|605/1015 | 18/28\|659/1082 | **130/180\|547/930** | 35/45\|642/1065 | **51/64\|626/1046** | **153/174\|524/936** | **138/179\|539/931** | **93/105\|584/1005** |
| Rv0278c-PE_PGRS4:336557:C:CT | IRG | **459/683\|143/427** | **406/578\|196/532** | **251/346\|351/764** | **410/599\|192/511** | 63/95\|539/1015 | 17/28\|585/1082 | 112/180\|490/930 | 31/45\|571/1065 | 45/64\|557/1046 | **138/174\|464/936** | **121/179\|481/931** | **81/105\|521/1005** |
| ephF:162151:GT:G | p.Phe129fs | **519/683\|159/427** | **458/578\|220/532** | **285/346\|393/764** | **467/599\|211/511** | 72/95\|606/1015 | 19/28\|659/1082 | 129/180\|549/930 | 35/45\|643/1065 | **52/64\|626/1046** | **153/174\|525/936** | **139/179\|539/931** | **93/105\|585/1005** |
| Rv3725:4170964:G:GA | p.Gly251fs | **517/683\|161/427** | **457/578\|221/532** | **284/346\|394/764** | **466/599\|212/511** | 72/95\|606/1015 | 19/28\|659/1082 | **130/180\|548/930** | 36/45\|642/1065 | **52/64\|626/1046** | **153/174\|525/936** | **139/179\|539/931** | **93/105\|585/1005** |
| Rv3847:4322039:AC:A | p.Ala169fs | **516/683\|159/427** | **456/578\|219/532** | **282/346\|393/764** | **465/599\|210/511** | 72/95\|603/1015 | 20/28\|655/1082 | 128/180\|547/930 | 34/45\|641/1065 | **52/64\|623/1046** | **152/174\|523/936** | **139/179\|536/931** | **93/105\|582/1005** |
| Rv0976c-PE_PGRS16:1090188:A:AG | IRG | **515/683\|161/427** | **455/578\|221/532** | **282/346\|394/764** | **464/599\|212/511** | 72/95\|604/1015 | 18/28\|658/1082 | **129/180\|547/930** | 33/45\|643/1065 | **51/64\|625/1046** | **153/174\|523/936** | **139/179\|537/931** | **93/105\|583/1005** |
| rplM-esxT:3862472:GA:G | IRG | **674/683\|393/427** | **567/578\|500/532** | 336/346\|731/764 | 593/599\|474/511 | -- | -- | 175/180\|892/930 | -- | -- | 174/174\|893/936 | 178/179\|889/931 | 105/105\|962/1005 |
| prfB-fprA:3473996:G:GA | IRG | **650/683\|346/427** | **551/578\|445/532** | **330/346\|666/764** | **570/599\|426/511** | 92/95\|904/1015 | -- | 168/180\|828/930 | -- | 63/64\|933/1046 | **174/174\|822/936** | **177/179\|819/931** | **103/105\|893/1005** |
| rseA-htrA:1365837:C:CGG | IRG | **518/683\|164/427** | **457/578\|225/532** | **282/346\|400/764** | **466/599\|216/511** | 71/95\|611/1015 | -- | **131/180\|551/930** | 32/45\|650/1065 | **51/64\|631/1046** | **153/174\|529/936** | **140/179\|542/931** | **94/105\|588/1005** |
| esxU:3863170:A:AGCATC | p.Leu32fs | 7/683\|0/427 | 7/578\|0/532 | 4/346\|3/764 | 7/599\|0/511 | 3/95\|4/1015 | -- | **6/180\|1/930** | -- | 1/64\|6/1046 | -- | 4/179\|3/931 | 1/105\|6/1005 |
| Rv1045:1168009:GC:G | p.Glu115fs | **506/683\|156/427** | **446/578\|216/532** | **277/346\|385/764** | **457/599\|205/511** | 71/95\|591/1015 | 18/28\|644/1082 | **127/180\|535/930** | 30/45\|632/1065 | **51/64\|611/1046** | **148/174\|514/936** | **137/179\|525/931** | **92/105\|570/1005** |
| whiB6-Rv3863:4338595:GC:G | IRG | **671/683\|394/427** | **566/578\|499/532** | 337/346\|728/764 | **590/599\|475/511** | 94/95\|971/1015 | 27/28\|1038/1082 | -- | -- | 62/64\|1003/1046 | 170/174\|895/936 | 178/179\|887/931 | 104/105\|961/1005 |
| vapC2-Rv0302:364498:TG:T | IRG | 19/683\|4/427 | **21/578\|2/532** | 15/346\|8/764 | 19/599\|4/511 | 3/95\|20/1015 | -- | 7/180\|16/930 | 1/45\|22/1065 | 5/64\|18/1046 | 10/174\|13/936 | **13/179\|10/931** | 6/105\|17/1005 |
| Rv3402c-Rv3403c:3822042:A:AC | IRG | 5/683\|0/427 | 5/578\|0/532 | 4/346\|1/764 | 5/599\|0/511 | 3/95\|2/1015 | -- | 4/180\|1/930 | -- | **3/64\|2/1046** | 3/174\|2/936 | 3/179\|2/931 | 3/105\|2/1005 |
| ctpI:125830:G:GA | p.Ser1571fs | **678/683\|403/427** | 571/578\|510/532 | 340/346\|741/764 | **595/599\|486/511** | 94/95\|987/1015 | -- | 176/180\|905/930 | -- | 63/64\|1018/1046 | 174/174\|907/936 | 179/179\|902/931 | 105/105\|976/1005 |
| Rv2084:2342649:A:AGGCGTACACAC | p.Ser282fs | **458/683\|146/427** | **408/578\|196/532** | **250/346\|354/764** | **415/599\|189/511** | 64/95\|540/1015 | 16/28\|588/1082 | 115/180\|489/930 | 31/45\|573/1065 | 46/64\|558/1046 | **137/174\|467/936** | **122/179\|482/931** | **81/105\|523/1005** |
| Rv0922-Rv0923c:1029386:G:GCGC | IRG | 8/683\|1/427 | 7/578\|2/532 | 6/346\|3/764 | 6/599\|3/511 | -- | -- | -- | **4/45\|5/1065** | -- | 4/174\|5/936 | -- | -- |
| dosT:2273733:TC:T | p.Glu259fs | **516/683\|158/427** | **456/578\|218/532** | **284/346\|390/764** | **465/599\|209/511** | 72/95\|602/1015 | 19/28\|655/1082 | **130/180\|544/930** | 35/45\|639/1065 | **51/64\|623/1046** | **153/174\|521/936** | **139/179\|535/931** | **93/105\|581/1005** |
| Rv2264c:2536628:G:GA | p.Pro575fs | **507/683\|156/427** | **447/578\|216/532** | **276/346\|387/764** | **457/599\|206/511** | 72/95\|591/1015 | 18/28\|645/1082 | **129/180\|534/930** | 29/45\|634/1065 | **52/64\|611/1046** | **149/174\|514/936** | **139/179\|524/931** | **93/105\|570/1005** |
| Rv0045c:49690:GCC:G | p.Gly83fs | **510/683\|161/427** | **450/578\|221/532** | **279/346\|392/764** | **459/599\|212/511** | 69/95\|602/1015 | 17/28\|654/1082 | 127/180\|544/930 | 33/45\|638/1065 | **51/64\|620/1046** | **151/174\|520/936** | **138/179\|533/931** | **92/105\|579/1005** |
| Rv1042c-Rv1043c:1165521:T:TA | IRG | **663/683\|392/427** | 557/578\|498/532 | 330/346\|725/764 | **583/599\|472/511** | 91/95\|964/1015 | -- | 173/180\|882/930 | -- | -- | 172/174\|883/936 | **179/179\|876/931** | 105/105\|950/1005 |
| Rv1145:1273250:G:GA | p.Leu277fs | **514/683\|157/427** | **454/578\|217/532** | **282/346\|389/764** | **463/599\|208/511** | 71/95\|600/1015 | 17/28\|654/1082 | **130/180\|541/930** | 35/45\|636/1065 | **51/64\|620/1046** | **153/174\|518/936** | **139/179\|532/931** | **93/105\|578/1005** |
| plcA-PPE38:2632341:C:CA | IRG | **514/683\|162/427** | **455/578\|221/532** | **285/346\|391/764** | **464/599\|212/511** | 73/95\|603/1015 | 20/28\|656/1082 | 126/180\|550/930 | 34/45\|642/1065 | **52/64\|624/1046** | **149/174\|527/936** | **138/179\|538/931** | **91/105\|585/1005** |
| Rv0197:234496:C:CGT | p.Pro756fs | **674/683\|406/427** | 567/578\|513/532 | -- | **592/599\|488/511** | -- | -- | -- | -- | -- | 174/174\|906/936 | 179/179\|901/931 | 105/105\|975/1005 |
| Rv2975c:3331361:ACG:A | p.Arg84fs | **510/683\|160/427** | **451/578\|219/532** | **279/346\|391/764** | **459/599\|211/511** | 71/95\|599/1015 | 17/28\|653/1082 | **129/180\|541/930** | 33/45\|637/1065 | **51/64\|619/1046** | **150/174\|520/936** | **138/179\|532/931** | **90/105\|580/1005** |
| Rv0759c-Rv0760c:854252:GCC:G | IRG | 45/683\|26/427 | 41/578\|30/532 | -- | -- | -- | 5/28\|66/1082 | -- | **10/45\|61/1065** | -- | 13/174\|58/936 | -- | -- |
| serB1-mmpS2:596701:CAGG:C | IRG | 7/683\|0/427 | 7/578\|0/532 | 4/346\|3/764 | 7/599\|0/511 | 3/95\|4/1015 | -- | **6/180\|1/930** | -- | 1/64\|6/1046 | -- | 4/179\|3/931 | 1/105\|6/1005 |
| Rv1861-adhA:2109523:C:CG | IRG | **673/683\|399/427** | 566/578\|506/532 | 338/346\|734/764 | **591/599\|481/511** | 93/95\|979/1015 | -- | 175/180\|897/930 | -- | 63/64\|1009/1046 | 174/174\|898/936 | 179/179\|893/931 | 105/105\|967/1005 |
| Rv2264c:2536625:C:CG | p.Gly576fs | 11/683\|2/427 | 10/578\|3/532 | 6/346\|7/764 | 8/599\|5/511 | -- | -- | -- | **5/45\|8/1065** | -- | 5/174\|8/936 | -- | -- |
| Rv3684-proY:4126514:GGT:G | IRG | 5/683\|0/427 | 5/578\|0/532 | 4/346\|1/764 | 5/599\|0/511 | 3/95\|2/1015 | -- | 4/180\|1/930 | -- | **3/64\|2/1046** | 3/174\|2/936 | 3/179\|2/931 | 3/105\|2/1005 |
| ndhA-Rv0393:472711:T:TTTGTGGGCC | IRG | **334/683\|99/427** | **287/578\|146/532** | **185/346\|248/764** | **310/599\|123/511** | 41/95\|392/1015 | -- | **100/180\|333/930** | -- | **39/64\|394/1046** | **89/174\|344/936** | **105/179\|328/931** | **72/105\|361/1005** |
| Rv2426c-proA:2724180:TCACGATCGGGTCTCCTCTAG:T | IRG | **417/683\|139/427** | **373/578\|183/532** | **232/346\|324/764** | **381/599\|175/511** | 56/95\|500/1015 | -- | **112/180\|444/930** | 27/45\|529/1065 | 40/64\|516/1046 | **127/174\|429/936** | **116/179\|440/931** | **80/105\|476/1005** |
| Rv0108c-PE_PGRS1:131174:T:TG | IRG | **679/683\|406/427** | 572/578\|513/532 | 340/346\|745/764 | **597/599\|488/511** | 95/95\|990/1015 | -- | 177/180\|908/930 | -- | 63/64\|1022/1046 | 174/174\|911/936 | 179/179\|906/931 | 105/105\|980/1005 |
| lipX-mshB:1300271:A:AT | IRG | 5/683\|0/427 | 5/578\|0/532 | 4/346\|1/764 | 5/599\|0/511 | 3/95\|2/1015 | -- | 4/180\|1/930 | -- | **3/64\|2/1046** | 3/174\|2/936 | 3/179\|2/931 | 3/105\|2/1005 |
| PPE21-fadD11:1753519:G:GC | IRG | **668/683\|387/427** | **562/578\|493/532** | 336/346\|719/764 | **586/599\|469/511** | 91/95\|964/1015 | -- | 174/180\|881/930 | -- | -- | 173/174\|882/936 | **179/179\|876/931** | 105/105\|950/1005 |
| vapB18:2867880:TA:T | p.Leu33fs | **519/683\|156/427** | **459/578\|216/532** | **285/346\|390/764** | **468/599\|207/511** | 72/95\|603/1015 | 19/28\|656/1082 | **130/180\|545/930** | 36/45\|639/1065 | **52/64\|623/1046** | **153/174\|522/936** | **139/179\|536/931** | **93/105\|582/1005** |

In the second column, the string with the prefix “p.” represents amino acid changes. For example, P.Glu115fs indicates that there was a frameshift mutation from the 115th amino acid (Glu) of the protein. “IGR” means that the indel existed in an intergenic region. The number before the “|” symbol represents the incidence rate of FS mutations or IGR indels among the resistant strains of a drug, which was the number of resistant strains with FS mutations in genes or with IGR indels in IGRs for a certain drug divided by the number of resistant strains for a certain drug. The number after the “|” symbol represents the incidence rate of FS mutations or IGR indels among the sensitive strains of the corresponding drug, which was the number of sensitive strains with FS mutations in genes or with IGR indels in IGRs for a certain drug divided by the number of sensitive strains for a certain drug. P-values were obtained by testing the incidence rate of FS mutations or IGR indels among the resistant strains of a drug versus the incidence rate of FS mutations or IGR indels among the sensitive strains of the corresponding drug. FDR method was used to do multiple testing correction. If both the adjusted chi-square and Fisher’s exact p-values were less than 0.01 when a marker was tested with a certain drug, then the marker was reserved. Bold font indicated that the adjusted p-values for both the chi-square and Fisher’s exact test were less than 0.01. The symbol “--” indicates that the incidence rate of FS or IGR indels in resistant strains of a drug was lower than the incidence rate in sensitive strains of the corresponding drug.

1The format of the string in the 1st column was region_name:postion_in_H37Rv_genome:reference_allele:alternative_allele.

**Table S11. The identified point markers of IGR indels located in the IGRs expressing sRNA**

| **Region makers (IGRs)** | **Mutation type** | **Point markers (IRG indels)** |
| --- | --- | --- |
| ctpE-Rv0909 | Intergenic region | 1014300:G:GT |
| lipX-mshB | Intergenic region | 1300271:A:AT |
| nrdH-Rv3054c | Intergenic region | 3415180:ACACCTAGGGGGTGG:A |
| PE12-fbiC | Intergenic region | 1302917:G:GA |
| rplM-esxT | Intergenic region | 3862472:GA:G |
| Rv0922-Rv0923c | Intergenic region | 1029386:G:GCGC |
| Rv1045-Rv1047 | Intergenic region | 1168715:C:CT |
| Rv1179c-pks3 | Intergenic region | 1313337:A:AG |
| Rv3402c-Rv3403c | Intergenic region | 3822042:A:AC |
| serB1-mmpS2 | Intergenic region | 596701:CAGG:C |

**Table S12. Overview of the functions of the 83 point markers**

| **Functions** | **Number of markers** |
| --- | --- |
| cell wall | 18 |
| Growth | 11 |
| Transporter | 3 |
| Antibiotics | 8 |
| transcript regulation | 10 |
| cell membrane | 40 |
| metabolism | 26 |

**Table S13. Descriptions of the functions of the 83 point markers**

| **Makers** | **Location** | **Description** |
| --- | --- | --- |
| Rv0272c:Lys374fs | coding region | hypothetical protein |
| argW-echA14:IGR | intergenic region | tRNA/ enoyl-CoA hydratase EchA14 |
| Rv2955c:His78fs | coding region | hypothetical protein |
| pncA:Val131fs | coding region | pyrazinamidase/nicotinamidase PncA |
| Rv3517:Gly37fs | coding region | hypothetical protein |
| pckA-nadR:IGR | intergenic region | phosphoenolpyruvate carboxykinase/ transcriptional regulator NadR |
| dxs2-Rv3382c:IGR | intergenic region | 1-deoxy-D-xylulose-5-phosphate synthase/ 4-hydroxy-3-methylbut-2-enyl diphosphate reductase |
| esxU:Leu32fs | coding region | ESAT-6 like protein EsxU |
| Rv2264c:Pro575fs | coding region | hypothetical protein |
| Rv0970-echA7:IGR | intergenic region | integral membrane protein/ enoyl-CoA hydratase EchA7 |
| Rv1042c-Rv1043c:IGR | intergenic region | IS2-like transposase/ hypothetical protein |
| Rv0739:Glu9fs | coding region | hypothetical protein |
| rplM-esxT:IGR | intergenic region | 50S ribosomal protein L13/ ESAT-6 like protein EsxT |
| mce2A-mce2C:IGR | intergenic region | Mce family protein Mce2A/ Mce family protein Mce2C |
| prfB-fprA:IGR | intergenic region | peptide chain release factor PrfB/ NADPH-ferredoxin reductase FprA |
| dosT:Glu259fs | coding region | two component sensor histidine kinase DosT |
| aceAa:Thr296fs | coding region | isocitrate lyase AceAa |
| PPE21-fadD11:IGR | intergenic region | PPE family protein PPE21/ fatty-acid--CoA ligase FadD11 |
| Rv3202a-lipV:IGR | intergenic region | hypothetical protein/ lipase LipV |
| vapB18:Leu33fs | coding region | antitoxin VapB18 |
| whiB6-Rv3863:IGR | intergenic region | transcriptional regulator WhiB6/ hypothetical protein |
| ppsA:Gln808fs | coding region | phthiocerol synthesis polyketide synthase type I PpsA |
| Rv0658c:Val101fs | coding region | integral membrane protein |
| serB1-mmpS2:IGR | intergenic region | phosphoserine phosphatase SerB/ membrane protein MmpS2 |
| Rv1928c:Ile55fs | coding region | short-chain type dehydrogenase/reductase |
| pks6:Asn28fs | coding region | membrane bound polyketide synthase |
| nrdH-Rv3054c:IGR | intergenic region | glutaredoxin electron transport protein NrdH/ hypothetical protein |
| Rv3750c-serX:IGR | intergenic region | excisionase/ tRNA |
| PE9-Rv1088a:IGR | intergenic region | PE family protein PE9/ hypothetical protein |
| ndhA-Rv0393:IGR | intergenic region | NADH dehydrogenase NdhA/ hypothetical protein |
| Rv3725:Gly251fs | coding region | oxidoreductase |
| Rv1225c:Gly134fs | coding region | hypothetical protein |
| Rv0045c:Gly83fs | coding region | hydrolase |
| Rv0922-Rv0923c:IGR | intergenic region | transposase/ hypothetical protein |
| Rv0197:Pro756fs | coding region | oxidoreductase |
| mazE3-Rv1106c:IGR | intergenic region | antitoxin MazE3/ 3 beta-hydroxysteroid dehydrogenase/delta 5-->4-isomerase |
| ephF:Phe129fs | coding region | epoxide hydrolase EphF |
| Rv3897c:Gly166fs | coding region | hypothetical protein |
| Rv2294-Rv2295:IGR | intergenic region | cystathionine beta-lyase/ hypothetical protein |
| Rv2975c:Arg84fs | coding region | hypothetical protein |
| ppsA:Gln808fs | coding region | phthiocerol synthesis polyketide synthase type I PpsA |
| ctpE-Rv0909:IGR | intergenic region | metal cation transporter ATPase E/ antitoxin |
| 1045:Glu115fs | coding region | hypothetical protein |
| Rv3684-proY:IGR | intergenic region | lyase/ tRNA |
| mpt53-cdsA:IGR | intergenic region | soluble secreted antigen Mpt53/ phosphatidate cytidylyltransferase |
| mce1R:Gly171fs | coding region | transcriptional regulator Mce1R |
| plcA-PPE38:IGR | intergenic region | membrane-associated phospholipase A/ PPE family protein PPE38 |
| Rv3830c:Ser208fs | coding region | TetR family transcriptional regulator |
| PE12-fbiC:IGR | intergenic region | PE family protein PE12/ FO synthase |
| Rv2081c:Val105fs | coding region | transmembrane protein |
| Rv0108c-PE_PGRS1:IGR | intergenic region | hypothetical protein/ PE-PGRS family protein PE_PGRS1 |
| Rv0194-Rv0195:IGR | intergenic region | multidrug ABC transporter ATPase/permease/ two component transcriptional regulator |
| Rv1775:Gln42fs | coding region | hypothetical protein |
| Rv0278c-PE_PGRS4:IGR | intergenic region | PE-PGRS family protein PE_PGRS3/ PE-PGRS family protein PE_PGRS4 |
| Rv3847:Ala169fs | coding region | hypothetical protein |
| ltp1:Ser6fs | coding region | lipid-transfer protein |
| Rv3747-Rv3748:IGR | intergenic region | hypothetical protein/ hypothetical protein |
| Rv0420c-Rv0421c:IGR | intergenic region | transmembrane protein/ hypothetical protein |
| Rv0976c-PE_PGRS16:IGR | intergenic region | hypothetical protein/ PE-PGRS family protein PE_PGRS16 |
| Rv3402c-Rv3403c:IGR | intergenic region | hypothetical protein/ hypothetical protein |
| Rv1179c-pks3:IGR | intergenic region | hypothetical protein/ polyketide beta-ketoacyl synthase |
| Rv1045-Rv1047:IGR | intergenic region | hypothetical protein/ transposase |
| sigM:Arg160fs | coding region | ECF RNA polymerase sigma factor SigM |
| sigG:Val16fs | coding region | ECF type sigma factor |
| pks15:Arg491fs | coding region | polyketide synthase |
| PE_PGRS55-PE_PGRS56:IGR | intergenic region | PE-PGRS family protein PE_PGRS55/ PE-PGRS family protein PE_PGRS56 |
| ctpI:Ser1571fs | coding region | cation-transporter ATPase I |
| Rv1861-adhA:IGR | intergenic region | transmembrane protein/ alcohol dehydrogenase A |
| Rv0759c-Rv0760c:IGR | intergenic region | hypothetical protein/ hypothetical protein |
| plsB1:Gly306fs | coding region | acyltransferase PlsB |
| Rv2293c:Ala222fs | coding region | hypothetical protein |
| Rv1145:Leu277fs | coding region | transmembrane transport protein |
| Rv2084:Ser282fs | coding region | hypothetical protein |
| Rv2264c:Gly576fs | coding region | hypothetical protein |
| rseA-htrA:IGR | intergenic region | anti-sigma E factor RseA/ serine protease HtrA |
| tgs3:Ser267fs | coding region | diacyglycerol O-acyltransferase |
| vapC2-Rv0302:IGR | intergenic region | ribonuclease VapC2/ transcriptional regulator |
| lipX-mshB:IGR | intergenic region | lipase LipX/ 1D-myo-inositol 2-acetamido-2-deoxy-alpha-D-glucopyranoside deacetylase |
| moeY:Val111fs | coding region | molybdopterin biosynthesis protein MoeY |
| Rv1714:Lys183fs | coding region | oxidoreductase |
| Rv2251:Glu55fs | coding region | flavoprotein |
| Rv0759c-Rv0760c:IGR | intergenic region | hypothetical protein/ hypothetical protein |
| Rv2426c-proA:IGR | intergenic region | hypothetical protein/ gamma-glutamyl phosphate reductase |

## **Table S14. The 6 point markers (FS mutations and IGR indels) out of the 83 point markers which exclusively occurred in resistant strains but not in sensitive strains of their associated drugs (strain number).**

| **Makers** | **Effect** | **INH** | **RIF** | **PZA** | **STR** | **EMB** | **OFX** | **MOX** | **ETH** | **KAN** | **AMI^1^** | **CAP** | | **PRO^2^** |
| --- | --- | --- | --- | --- | --- | --- | --- | --- | --- | --- | --- | --- | --- | --- |
| Rv2955c:3308313:T:TG | p.His78fs | **18/683,0/427** | 15/578,3/532 | **9/180,9/930** | **18/599,0/511** | 12/346,6/764 | 3/174,15/936 | 3/105,15/1005 | -- | -- | 2/64,16/1046 | 5/95,13/1015 | 6/179,12/931 | |
| argW-echA14:2794344:TG:T | IRG | 15/683,0/427 | 14/578,1/532 | 4/180,11/930 | **15/599,0/511** | -- | 3/174,12/936 | 2/105,13/1005 | -- | -- | -- | -- | **8/179,7/931** | |
| pckA-nadR:253609:CAGACGCATAAGCCCCCGCACGCACGGCGTGTCGAGGGCTTT:C | IRG | 4/683,0/427 | 4/578,0/532 | **4/180,0/930** | 4/599,0/511 | 2/346,2/764 | 1/174,3/936 | 1/105,3/1005 | -- | -- | -- | 2/95,2/1015 | 2/179,2/931 | |
| pncA:2288850:A:ACC | p.Val131fs | 4/683,0/427 | 3/578,1/532 | **4/180,0/930** | 4/599,0/511 | 3/346,1/764 | -- | -- | -- | -- | 1/64,3/1046 | 1/95,3/1015 | 3/179,1/931 | |
| Rv3517:3953533:T:TC | p.Gly37fs | 4/683,0/427 | 4/578,0/532 | **4/180,0/930** | 4/599,0/511 | 3/346,1/764 | 3/174,1/936 | 3/105,1/1005 | -- | -- | 1/64,3/1046 | 2/95,2/1015 | 2/179,2/931 | |
| Rv0272c:328589:TG:T | p.Lys374fs | **52/683,0/427** | 28/578,24/532 | **18/180,34/930** | **52/599,0/511** | 20/346,32/764 | -- | 7/105,45/1005 | -- | -- | 7/64,45/1046 | -- | -- | |

If one marker occurred in resistant strains of one drug, and did not occur in sensitive strains of the same drug, then this marker was put into this table. In the second column, the string with prefix “p.” represented amino acid change. For example, P.Met286fs meant that there was a frameshift mutation from 286th amino acid (Met) of the protein. “IGR” represented the indel was existing in an intergenic region. The string in front of each comma represented incidence rate of FS mutations or IGR indels among the resistant strains of a drug, which was the number of resistant strains with FS mutations in genes or with IGR indels in IGRs for a certain drug divided by the number of resistant strains for a certain drug. The string behind each comma represented the incidence rate of FS mutations or IGR indels among the sensitive strains of the corresponding drug, which was the number of sensitive strains with FS mutations in genes or with IGR indels in IGRs for a certain drug divided by the number of sensitive strains for a certain drug. The symbol “--“ meant the incidence rate of FS or IGR indels in resistant strains of a drug was lower than the incidence rate in sensitive strains of the corresponding drug. Bold font indicated adjusted p-values of both chi-square test and fisher’s exact test were significant.

## **Table S15. P-values after logistic regression for the associations between the 20 region markers and drug resistance**

| **Markers** | **INH** | **RIF** | **PZA** | **STR** | **EMB** | **OFX** | **MOX** | **ETH** | **KAN** | **AMI** | **CAP** | **PRO** |
| --- | --- | --- | --- | --- | --- | --- | --- | --- | --- | --- | --- | --- |
| purM-Rv0810c | nan | nan | nan | nan | nan | nan | nan | 3.25E-03 | nan | nan | nan | nan |
| rseA-htrA | 6.93E-02 | 3.48E-01 | nan | 5.07E-01 | nan | 3.16E-03 | nan | nan | nan | nan | nan | nan |
| ndhA-Rv0393 | 6.52E-02 | 1.01E-01 | 6.45E-04 | 8.33E-02 | 5.07E-01 | 4.24E-01 | 1.06E-03 | nan | nan | 9.37E-02 | nan | 1.72E-02 |
| vapC2-Rv0302 | nan | 2.13E-04 | nan | 1.92E-01 | 1.17E-01 | 5.67E-02 | nan | nan | nan | 2.13E-01 | nan | 3.16E-03 |
| Rv0759c-Rv0760c | 2.94E-01 | 2.33E-01 | nan | 4.98E-01 | 4.50E-01 | nan | nan | nan | nan | nan | nan | nan |
| Rv1995 | 1.13E-12 | 7.08E-01 | 7.91E-07 | 1.09E-05 | 6.10E-01 | 1.53E-01 | 1.65E-01 | nan | nan | 3.40E-01 | nan | 1.64E-01 |
| PE_PGRS55-PE_PGRS56 | 2.69E-02 | 1.95E-01 | 2.04E-01 | 1.54E-01 | 6.55E-01 | 5.07E-01 | 8.41E-01 | nan | nan | 5.66E-01 | nan | 5.27E-01 |
| Rv1509 | nan | 4.98E-01 | 4.34E-04 | 4.13E-01 | 3.45E-01 | 2.04E-03 | 2.27E-01 | nan | nan | nan | nan | 7.77E-01 |
| ddn-Rv3548c | 3.11E-04 | 1.00E+00 | 1.67E-01 | 2.90E-01 | 1.00E+00 | 1.88E-02 | 4.71E-01 | nan | nan | 8.41E-01 | 9.20E-01 | 3.30E-01 |
| Rv3848-espR | 2.69E-02 | 3.39E-02 | nan | 3.77E-01 | nan | nan | nan | nan | nan | nan | nan | nan |
| Rv2081c | 2.21E-01 | 2.43E-02 | nan | 3.68E-01 | 1.00E+00 | nan | nan | nan | nan | nan | nan | nan |
| pncA | 1.48E-03 | 1.82E-01 | 1.20E-06 | 7.77E-01 | 3.80E-01 | nan | nan | nan | nan | nan | nan | nan |
| whiB6-Rv3863 | 7.34E-04 | 1.63E-01 | nan | 1.65E-01 | nan | nan | nan | nan | nan | nan | nan | nan |
| Rv2571c | nan | nan | nan | nan | nan | 3.18E-02 | nan | nan | nan | nan | nan | nan |
| ethA | 3.08E-07 | 1.38E-01 | nan | 1.00E+00 | 2.51E-01 | 5.78E-01 | 9.20E-01 | nan | nan | nan | 2.03E-01 | 1.08E-02 |
| PPE36-prcA | 4.83E-02 | nan | nan | 2.96E-01 | nan | nan | nan | nan | nan | nan | nan | nan |
| kdpD | 7.93E-13 | 7.91E-01 | 1.30E-06 | 2.31E-04 | 6.10E-01 | 4.39E-02 | 4.13E-01 | nan | nan | 1.81E-01 | nan | 1.29E-01 |
| Rv0011c-Rv0012 | nan | nan | nan | nan | nan | 3.88E-03 | nan | nan | nan | nan | nan | nan |
| mce2A-mce2C | 2.17E-03 | 6.10E-01 | 5.71E-02 | 1.75E-01 | 6.63E-01 | 4.44E-02 | 3.99E-01 | nan | nan | 6.80E-01 | 5.78E-01 | 6.03E-02 |
| Rv3750c-serX | 2.95E-04 | 2.47E-01 | nan | 6.80E-01 | 6.32E-01 | 1.16E-03 | 4.54E-01 | nan | nan | nan | nan | 5.92E-04 |

The region markers which were no significant after logistic regression analysis was highlighted. “nan” indicates no association was identified in previous paragraph of this study.

## **Table S16. P-values after logistic regressions for the associations between the 83 point markers and drug resistance**

| Markers | INH | RIF | PZA | STR | EMB | OFX | MOX | ETH | KAN | AMI | CAP | PRO |
| --- | --- | --- | --- | --- | --- | --- | --- | --- | --- | --- | --- | --- |
| Rv0045c:49690:GCC:G | 4.04E-02 | 9.20E-01 | nan | 7.45E-02 | 8.88E-01 | 7.69E-02 | 8.88E-01 | nan | nan | 6.99E-01 | nan | 6.71E-01 |
| pks15:3296371:G:GCCGCGGC | 3.81E-02 | 7.52E-01 | 5.34E-02 | 5.92E-02 | 6.03E-01 | 3.00E-02 | 2.90E-01 | nan | nan | 6.32E-01 | nan | 6.47E-01 |
| Rv1145:1273250:G:GA | 5.92E-04 | 7.40E-01 | 8.38E-02 | 1.66E-01 | 7.52E-01 | 1.41E-02 | 5.02E-01 | nan | nan | 8.88E-01 | nan | 4.13E-01 |
| Rv0759c-Rv0760c:854252:GC:G | nan | nan | nan | 1.90E-01 | nan | nan | 4.99E-04 | nan | nan | nan | nan | nan |
| rseA-htrA:1365837:C:CGG | 6.90E-03 | 8.88E-01 | 1.59E-01 | 8.86E-02 | 6.03E-01 | 4.97E-02 | 8.88E-01 | nan | nan | 7.64E-01 | nan | 3.57E-01 |
| mpt53-cdsA:3190145:TC:T | 5.32E-04 | 1.44E-01 | nan | 5.49E-01 | 7.52E-01 | 3.58E-04 | 9.20E-01 | nan | nan | nan | nan | 1.03E-04 |
| Rv1714:1942396:GC:G | nan | nan | nan | nan | nan | nan | nan | nan | nan | 6.33E-02 | nan | nan |
| pckA-nadR:253609:CAGACGCATAAGCCCCCGCACGCACGGCGTGTCGAGGGCTTT:C | nan | nan | 1.00E+00 | nan | nan | nan | nan | nan | nan | nan | nan | nan |
| pncA:2288850:A:ACC | nan | nan | 4.65E-04 | nan | nan | nan | nan | nan | nan | nan | nan | nan |
| esxU:3863170:A:AGCATC | nan | nan | 4.33E-03 | nan | nan | nan | nan | nan | nan | nan | nan | nan |
| Rv3750c-serX:4198611:CG:C | 2.95E-04 | 2.47E-01 | nan | 6.80E-01 | 6.32E-01 | 1.16E-03 | 4.54E-01 | nan | nan | nan | nan | 5.92E-04 |
| plsB1:1756358:CG:C | 7.42E-05 | 4.98E-01 | 2.09E-01 | 2.44E-01 | 4.75E-01 | 6.76E-02 | 3.68E-01 | nan | nan | 7.18E-01 | nan | 1.37E-01 |
| Rv2955c:3308313:T:TG | 3.93E-06 | nan | 3.68E-02 | 1.58E-02 | nan | nan | nan | nan | nan | nan | nan | nan |
| Rv1179c-pks3:1313337:A:AG | 9.12E-03 | nan | nan | 1.20E-01 | nan | nan | nan | nan | nan | nan | nan | nan |
| PE_PGRS55-PE_PGRS56:3943744:CCGGCAACGG:C | 1.80E-02 | 3.57E-01 | nan | 1.18E-01 | 7.08E-01 | 6.17E-01 | 2.77E-01 | nan | nan | nan | nan | 3.86E-01 |
| Rv0420c-Rv0421c:507028:GC:G | 9.17E-05 | 5.97E-01 | 3.74E-01 | 2.37E-01 | 4.06E-01 | 3.18E-02 | 4.88E-01 | nan | nan | 5.43E-01 | nan | 1.11E-01 |
| Rv3684-proY:4126514:GGT:G | nan | nan | nan | nan | nan | nan | nan | nan | nan | 6.33E-02 | nan | nan |
| Rv2426c-proA:2724180:TCACGATCGGGTCTCCTCTAG:T | 1.00E+00 | 2.18E-01 | 4.07E-02 | 7.52E-01 | 1.00E+00 | 2.24E-01 | 7.08E-01 | nan | nan | nan | nan | 7.77E-01 |
| aceAa:2161343:G:GT | 4.04E-02 | 1.04E-01 | nan | 5.51E-02 | 1.00E+00 | 4.00E-02 | 5.12E-01 | nan | nan | nan | nan | 3.17E-01 |
| Rv2975c:3331361:ACG:A | 1.51E-02 | 6.24E-01 | 1.52E-01 | 9.19E-02 | 8.88E-01 | 2.92E-01 | 1.03E-01 | nan | nan | 7.64E-01 | nan | 6.71E-01 |
| vapB18:2867880:TA:T | 1.53E-05 | 4.20E-01 | 2.52E-01 | 2.96E-01 | 6.47E-01 | 8.97E-02 | 3.37E-01 | nan | nan | 7.64E-01 | nan | 9.60E-02 |
| Rv2081c:2338194:A:AC | 1.80E-02 | 4.66E-02 | nan | 4.71E-01 | 1.00E+00 | nan | nan | nan | nan | nan | nan | nan |
| sigG:214096:C:CA | nan | nan | 4.33E-03 | nan | nan | nan | nan | nan | nan | nan | nan | nan |
| Rv2293c:2564368:G:GC | 1.21E-01 | 3.30E-01 | 9.80E-03 | 7.64E-01 | 6.80E-01 | 2.64E-01 | 6.03E-02 | nan | nan | 3.51E-01 | nan | 4.31E-01 |
| PPE21-fadD11:1753519:G:GC | 2.70E-03 | 5.54E-02 | nan | 4.17E-01 | nan | nan | nan | nan | nan | nan | nan | 1.70E-04 |
| Rv0739:830868:G:GGC | 3.43E-01 | 1.79E-07 | nan | 3.60E-04 | 5.60E-01 | 1.51E-02 | 2.56E-01 | nan | nan | nan | nan | 4.10E-01 |
| Rv3517:3953533:T:TC | nan | nan | 8.40E-04 | nan | nan | nan | nan | nan | nan | nan | nan | nan |
| ephF:162151:GT:G | 1.93E-04 | 8.23E-01 | nan | 1.68E-01 | 4.67E-01 | 6.33E-02 | 4.20E-01 | nan | nan | 6.71E-01 | nan | 1.67E-01 |
| Rv0970-echA7:1081735:G:GT | nan | nan | nan | nan | nan | nan | nan | nan | nan | 6.33E-02 | nan | nan |
| PE9-Rv1088a:1215104:AT:A | 1.19E-03 | 6.71E-01 | 3.96E-01 | 1.66E-01 | 4.27E-01 | 1.62E-01 | 2.47E-01 | nan | nan | 5.84E-01 | nan | 1.45E-01 |
| Rv0197:234496:C:CGT | 2.39E-02 | nan | nan | 1.55E-01 | nan | nan | nan | nan | nan | nan | nan | nan |
| serB1-mmpS2:596701:CAGG:C | nan | nan | 4.33E-03 | nan | nan | nan | nan | nan | nan | nan | nan | nan |
| whiB6-Rv3863:4338595:GC:G | 1.33E-03 | 3.13E-01 | nan | 9.66E-02 | nan | nan | nan | nan | nan | nan | nan | nan |
| Rv2251:2525722:CG:C | 2.56E-03 | nan | nan | 4.20E-01 | nan | nan | nan | nan | nan | nan | nan | nan |
| PE12-fbiC:1302917:G:GA | nan | nan | nan | nan | nan | nan | nan | nan | nan | 6.33E-02 | nan | nan |
| Rv2294-Rv2295:2566766:C:CG | 3.01E-03 | 5.90E-01 | nan | 1.24E-01 | 6.89E-01 | 8.59E-02 | 7.64E-01 | nan | nan | 7.64E-01 | nan | 1.00E+00 |
| moeY:1523702:CG:C | 2.42E-09 | 4.01E-06 | nan | 1.02E-02 | 5.97E-01 | nan | 3.92E-04 | nan | nan | nan | nan | 3.03E-01 |
| Rv1045-Rv1047:1168715:C:CT | 6.52E-03 | nan | nan | 9.54E-02 | nan | nan | nan | nan | nan | nan | nan | nan |
| nrdH-Rv3054c:3415180:ACACCTAGGGGGTGG:A | 1.62E-06 | 4.86E-02 | nan | 1.16E-01 | nan | 4.14E-03 | nan | nan | nan | nan | nan | 2.65E-04 |
| ltp1:3100154:A:AC | 0.00E+00 | 5.28E-10 | nan | 9.19E-02 | 4.50E-01 | 1.03E-02 | 1.49E-02 | nan | nan | nan | nan | 4.62E-01 |
| rplM-esxT:3862472:GA:G | 5.61E-04 | 3.79E-02 | nan | 2.83E-02 | nan | nan | nan | nan | nan | nan | nan | nan |
| tgs3:3610391:A:AC | 1.84E-03 | 7.29E-01 | 3.32E-01 | 1.50E-01 | 5.72E-01 | 2.07E-02 | 5.17E-01 | nan | nan | 8.88E-01 | nan | 1.54E-01 |
| prfB-fprA:3473996:G:GA | 3.36E-03 | 3.57E-01 | nan | 8.62E-01 | 2.84E-01 | 4.53E-04 | 5.32E-01 | nan | nan | nan | nan | 6.42E-04 |
| lipX-mshB:1300271:A:AT | nan | nan | nan | nan | nan | nan | nan | nan | nan | 6.33E-02 | nan | nan |
| pks6:485810:CA:C | 5.32E-04 | 6.47E-01 | 3.90E-01 | 1.88E-01 | 8.62E-01 | 3.93E-01 | 5.07E-01 | nan | nan | 5.22E-01 | nan | 3.65E-01 |
| Rv3830c:4305063:G:GA | 9.17E-05 | 5.97E-01 | 3.74E-01 | 2.37E-01 | 7.29E-01 | 2.72E-02 | 4.93E-01 | nan | nan | 8.41E-01 | nan | 3.13E-01 |
| mazE3-Rv1106c:1232563:CCTTACGT:C | nan | nan | 4.33E-03 | nan | nan | nan | nan | nan | nan | nan | nan | nan |
| Rv1042c-Rv1043c:1165521:T:TA | 7.36E-02 | nan | nan | 2.38E-01 | nan | nan | nan | nan | nan | nan | nan | 3.90E-05 |
| Rv1928c:2181054:A:ATC | nan | nan | 4.33E-03 | nan | nan | nan | nan | nan | nan | nan | nan | nan |
| mce1R:194305:C:CGG | 8.62E-03 | 8.06E-01 | 1.15E-01 | 1.22E-01 | 6.32E-01 | 1.63E-02 | 4.71E-01 | nan | nan | 8.62E-01 | nan | 3.43E-01 |
| Rv3747-Rv3748:4197138:C:CT | 3.96E-03 | 4.03E-01 | nan | 5.97E-01 | 4.39E-01 | 1.34E-03 | 4.10E-01 | nan | nan | nan | nan | 1.85E-03 |
| Rv1225c:1368322:C:CG | 2.10E-05 | 5.17E-01 | 4.39E-01 | 2.88E-01 | 2.79E-01 | 4.77E-02 | 4.58E-01 | nan | nan | 6.89E-01 | nan | 2.30E-01 |
| Rv2264c:2536625:C:CG | nan | nan | nan | nan | nan | nan | nan | 1.25E-02 | nan | nan | nan | nan |
| Rv0272c:328589:TG:T | 2.58E-08 | nan | 8.67E-03 | 2.64E-05 | nan | nan | nan | nan | nan | nan | nan | nan |
| vapC2-Rv0302:364498:TG:T | nan | 2.49E-04 | nan | nan | nan | nan | nan | nan | nan | nan | nan | 1.92E-03 |
| Rv1045:1168009:GC:G | 5.84E-03 | 8.41E-01 | 3.40E-01 | 4.93E-01 | 8.88E-01 | 4.42E-01 | 5.22E-01 | nan | nan | 6.71E-01 | nan | 5.60E-01 |
| dxs2-Rv3382c:3794867:C:CCA | 6.17E-03 | 9.20E-01 | nan | 1.24E-01 | 5.90E-01 | 3.86E-02 | 8.62E-01 | nan | nan | 8.41E-01 | nan | 4.10E-01 |
| plcA-PPE38:2632341:C:CA | 8.62E-03 | 3.86E-01 | nan | 4.62E-01 | 1.00E-01 | 8.23E-01 | 5.22E-01 | nan | nan | 4.98E-01 | nan | 2.67E-01 |
| Rv3897c:4383144:C:CCGGGG | 5.13E-02 | nan | nan | 9.54E-02 | nan | nan | nan | nan | nan | nan | nan | nan |
| argW-echA14:2794344:TG:T | nan | nan | nan | 2.00E-03 | nan | nan | nan | nan | nan | nan | nan | 7.41E-02 |
| Rv0278c-PE_PGRS4:336557:C:CT | 9.43E-02 | 7.08E-01 | nan | 6.52E-02 | 7.64E-01 | 1.24E-01 | 3.74E-01 | nan | nan | nan | nan | 4.62E-01 |
| Rv0922-Rv0923c:1029386:G:GCGC | nan | nan | nan | nan | nan | nan | nan | 1.96E-02 | nan | nan | nan | nan |
| ndhA-Rv0393:472711:T:TTTGTGGGCC | 3.59E-02 | 1.99E-01 | 2.60E-04 | 5.22E-02 | 6.24E-01 | 2.73E-01 | 3.02E-04 | nan | nan | 1.46E-01 | nan | 5.00E-02 |
| Rv2264c:2536628:G:GA | 4.43E-03 | 8.88E-01 | 6.17E-02 | 2.44E-01 | 5.90E-01 | 2.54E-01 | 3.74E-01 | nan | nan | 2.92E-01 | nan | 1.00E+00 |
| ppsA:3247865:GCAAA:G | 2.26E-04 | 4.58E-02 | nan | 4.42E-01 | nan | nan | 2.21E-02 | nan | nan | nan | nan | 1.27E-03 |
| Rv3847:4322039:AC:A | 1.19E-03 | 6.71E-01 | nan | 1.76E-01 | 9.20E-01 | 1.22E-01 | 9.20E-01 | nan | nan | 5.66E-01 | nan | 3.45E-01 |
| dosT:2273733:TC:T | 5.32E-04 | 6.47E-01 | 1.37E-01 | 1.79E-01 | 4.67E-01 | 3.35E-02 | 4.35E-01 | nan | nan | 6.89E-01 | nan | 2.52E-01 |
| Rv0108c-PE_PGRS1:131174:T:TG | 1.02E-03 | nan | nan | 1.90E-02 | nan | nan | nan | nan | nan | nan | nan | nan |
| Rv3725:4170964:G:GA | 2.70E-03 | 6.24E-01 | 2.03E-01 | 1.65E-01 | 6.63E-01 | 4.44E-02 | 3.99E-01 | nan | nan | 6.80E-01 | nan | 1.92E-01 |
| ppsA:3247864:C:CTAGG | 1.20E-04 | 5.99E-02 | nan | 3.48E-01 | nan | nan | 6.93E-02 | nan | nan | nan | nan | 2.34E-03 |
| Rv0658c:754108:AC:A | nan | nan | nan | nan | nan | nan | nan | nan | nan | 6.33E-02 | nan | nan |
| Rv3402c-Rv3403c:3822042:A:AC | nan | nan | nan | nan | nan | nan | nan | nan | nan | 6.33E-02 | nan | nan |
| Rv0759c-Rv0760c:854252:GCC:G | nan | nan | nan | nan | nan | nan | nan | 1.35E-02 | nan | nan | nan | nan |
| Rv3202a-lipV:3580636:CT:C | 8.63E-04 | nan | nan | 3.51E-01 | nan | nan | nan | nan | nan | nan | nan | nan |
| ctpI:125830:G:GA | 3.47E-04 | nan | nan | 1.79E-01 | nan | nan | nan | nan | nan | nan | nan | nan |
| sigM:4400660:AC:A | nan | nan | nan | 1.89E-02 | nan | nan | nan | nan | nan | nan | nan | 5.10E-04 |
| ctpE-Rv0909:1014300:G:GT | nan | nan | 4.33E-03 | nan | nan | nan | nan | nan | nan | nan | nan | nan |
| Rv1775:2009290:GC:G | 2.29E-03 | 1.00E+00 | 4.50E-02 | 2.92E-01 | 7.64E-01 | 1.00E+00 | 7.91E-01 | nan | nan | 4.03E-01 | nan | 7.29E-01 |
| Rv0194-Rv0195:230576:G:GT | 4.83E-02 | 6.39E-01 | nan | 1.23E-01 | 9.20E-01 | 2.69E-01 | 1.73E-01 | nan | nan | 7.18E-01 | nan | 1.85E-01 |
| Rv1861-adhA:2109523:C:CG | 9.64E-03 | nan | nan | 2.67E-01 | nan | nan | nan | nan | nan | nan | nan | nan |
| Rv2084:2342649:A:AGGCGTACACAC | 3.43E-01 | 2.42E-01 | nan | 7.91E-01 | 6.10E-01 | 2.90E-01 | 2.37E-01 | nan | nan | nan | nan | 3.71E-01 |
| mce2A-mce2C:688792:T:TG | 1.19E-03 | 5.97E-01 | 1.99E-01 | 1.87E-01 | 6.55E-01 | 4.44E-02 | 3.99E-01 | nan | nan | 6.80E-01 | nan | 6.06E-02 |
| Rv0976c-PE_PGRS16:1090188:A:AG | 6.90E-03 | 7.64E-01 | 3.57E-01 | 1.28E-01 | 9.20E-01 | 1.77E-02 | 5.22E-01 | nan | nan | 8.41E-01 | nan | 3.90E-01 |

The region markers which were no significant after logistic regression analysis was highlighted. “nan” indicates no association was identified in previous paragraph of this study.

## **Table S17. Effect of six mutations on the DNA repair genes**

| \| **GENE_NAME** \| **Coding product** \| **Function** \| **VARIANT_TYPE** \| **REF_AMINO** \| **ALT_AMINO** \| **AMINO_POS** \| **SIFT_PREDICTION** \| \| --- \| --- \| --- \| --- \| --- \| --- \| --- \| --- \| \| alkA \| bifunctional regulatory protein/DNA repair enzyme \| Base excision repair \| NONSYNONYMOUS \| I \| V \| 12 \| TOLERATED \| \| mutT4 \| mutator protein MutT \| hydrolase activity/ metal ion binding \| NONSYNONYMOUS \| R \| G \| 48 \| TOLERATED \| \| nth \| endonuclease III \| Base excision repair \| NONSYNONYMOUS \| P \| R \| 2 \| DELETERIOUS \| \| recD \| exonuclease V subunit alpha RecD \| double-strand break repair via homologous recombination \| NONSYNONYMOUS \| E \| D \| 120 \| DELETERIOUS \| \| uvrC \| excinuclease ABC subunit UvrC \| Nucleotide excision repair/ excinuclease repair complex \| NONSYNONYMOUS \| V \| I \| 289 \| TOLERATED \| \| uvrC \| excinuclease ABC subunit UvrC \| Nucleotide excision repair/ excinuclease repair complex \| NONSYNONYMOUS \| V \| A \| 434 \| DELETERIOUS \| |  |  |  |  |  |  |  |
| --- | --- | --- | --- | --- | --- | --- | --- | --- | --- | --- | --- | --- | --- | --- | --- | --- | --- | --- | --- | --- | --- | --- | --- | --- | --- | --- | --- | --- | --- | --- | --- | --- | --- | --- | --- | --- | --- | --- | --- | --- | --- | --- | --- | --- | --- | --- | --- | --- | --- | --- | --- | --- | --- | --- | --- | --- | --- | --- | --- | --- | --- | --- | --- |

## **Table S18. Region markers in the validation set that were overlapping with the 20 region markers identified in this study**

| **Markers** | **INH** | **RIF** | **PZA** | **STR** | **EMB** | **OFX** | **MOX** | **KAN** | **AMI** | **CAP** |
| --- | --- | --- | --- | --- | --- | --- | --- | --- | --- | --- |
| Rv2571c | 0.01\|0.05 | 0.00\|0.03 | -- | 0.32\|0.37 | 0.00\|0.00 | -- | -- | -- | -- | -- |
| kdpD | 0.29\|0.41 | -- | -- | -- | -- | -- | 0.89\|0.79 | 0.00\|0.04 | -- | -- |
| Rv1995 | 0.00\|0.00 | 0.00\|0.00 | 0.00\|0.00 | 0.00\|0.00 | 0.00\|0.00 | 0.01\|0.09 | 0.00\|0.01 | 0.00\|0.00 | 0.00\|0.00 | 0.31\|0.33 |
| Rv0759c-Rv0760c | 0.91\|0.96 | 0.44\|0.48 | 0.23\|0.29 | 0.39\|0.44 | 0.01\|0.01 | -- | -- | -- | 0.23\|0.33 | 0.89\|1 |
| Rv3750c-serX | 0.00\|0.00 | 0.00\|0.00 | 0.00\|0.00 | 0.00\|0.00 | 0.00\|0.00 | 0.85\|1 | -- | 0.30\|0.41 | 0.02\|0.04 | 0.07\|0.12 |
| Rv2081c | 0.00\|0.00 | 0.00\|0.00 | 0.24\|0.29 | 0.03\|0.06 | 0.00\|0.00 | -- | -- | -- | 0.01\|0.03 | 0.14\|0.19 |
| ddn-Rv3548c | 0.00\|0.00 | 0.00\|0.00 | 0.00\|0.00 | 0.00\|0.00 | 0.00\|0.00 | 0.00\|0.00 | 0.00\|0.01 | 0.00\|0.00 | 0.00\|0.00 | 0.00\|0.00 |
| pncA | 0.00\|0.00 | 0.00\|0.00 | 0.00\|0.00 | 0.00\|0.00 | 0.00\|0.00 | 0.00\|0.00 | 0.00\|0.00 | 0.00\|0.02 | 0.00\|0.02 | 0.00\|0.03 |
| ethA | 0.00\|0.00 | 0.00\|0.00 | 0.00\|0.00 | 0.00\|0.00 | 0.00\|0.00 | -- | 0.03\|0.20 | 0.01\|0.17 | 0.00\|0.01 | 0.01\|0.12 |
| PE_PGRS55-PE_PGRS56 | 0.00\|0.00 | 0.00\|0.00 | 0.00\|0.00 | 0.00\|0.00 | 0.00\|0.00 | 0.00\|0.01 | 0.58\|0.62 | 0.00\|0.00 | 0.00\|0.00 | 0.00\|0.00 |
| ndhA-Rv0393 | 0.00\|0.00 | 0.00\|0.00 | 0.00\|0.00 | 0.00\|0.00 | 0.00\|0.00 | 0.01\|0.03 | 0.69\|0.57 | 0.02\|0.06 | 0.00\|0.00 | 0.00\|0.00 |
| PPE36-prcA | 0.15\|0.29 | 0.01\|0.01 | 0.00\|0.00 | 0.00\|0.00 | 0.00\|0.00 | -- | -- | -- | 0.02\|0.01 | 0.61\|0.68 |
| mce2A-mce2C | 0.00\|0.00 | 0.00\|0.00 | 0.00\|0.00 | 0.00\|0.00 | 0.00\|0.00 | 0.05\|0.08 | 0.80\|0.84 | 0.00\|0.02 | 0.00\|0.00 | 0.00\|0.00 |

FDR was used to do multiple testing correction. The number in front of the “|” was the adjusted p-value of chi-square test. The number behind the “|” was the adjusted p-value of fisher’s exact test. P-values were obtained by testing the incidence rate of FS mutations or IGR indels among the resistant strains of a drug versus the incidence rate of FS mutations or IGR indels among the sensitive strains of the corresponding drug. Two decimal places were kept for each p-value. If both chi-square p-value and fisher’s exact p-value were less than 0.05 when a marker was tested with a certain drug, then the marker was reserved. The symbol “--“ meant the incidence rate of FS or IGR indels in resistant strains of a drug was lower than the incidence rate in sensitive strains of the corresponding drug.

**Table S19. Point markers in the validation set that were overlapping with the 83 indel markers identified in this study**

| **Markers** | **INH** | **RIF** | **PZA** | **STR** | **EMB** | **OFX** | **MOX** | **KAN** | **AMI** | **CAP** |
| --- | --- | --- | --- | --- | --- | --- | --- | --- | --- | --- |
| Rv2294-Rv2295:2566766:C:CG | 0.00\|0.00 | 0.00\|0.00 | 0.00\|0.00 | 0.00\|0.00 | 0.00\|0.00 | 0.04\|0.05 | 0.78\|0.83 | 0.00\|0.02 | 0.00\|0.00 | 0.00\|0.00 |
| Rv3747-Rv3748:4197138:C:CT | 0.00\|0.00 | 0.00\|0.00 | 0.00\|0.00 | 0.00\|0.00 | 0.00\|0.00 | 0.09\|0.12 | 0.43\|0.64 | 0.15\|0.20 | 0.00\|0.00 | 0.05\|0.06 |
| sigM:4400660:AC:A | 0.29\|0.35 | 0.16\|0.22 | 0.42\|0.47 | 0.00\|0.01 | 0.00\|0.00 | -- | -- | -- | 0.10\|0.15 | 0.30\|0.41 |
| Rv1225c:1368322:C:CG | 0.00\|0.00 | 0.00\|0.00 | 0.00\|0.00 | 0.00\|0.00 | 0.00\|0.00 | 0.00\|0.00 | 0.00\|0.00 | 0.00\|0.00 | 0.00\|0.00 | 0.00\|0.00 |
| Rv1775:2009290:GC:G | 0.00\|0.00 | 0.00\|0.00 | 0.00\|0.00 | 0.00\|0.00 | 0.00\|0.00 | 0.00\|0.00 | 0.27\|0.26 | 0.00\|0.00 | 0.00\|0.00 | 0.00\|0.00 |
| Rv3847:4322039:AC:A | 0.00\|0.00 | 0.00\|0.00 | 0.00\|0.00 | 0.00\|0.00 | 0.00\|0.00 | 0.00\|0.00 | 0.23\|0.24 | 0.00\|0.00 | 0.00\|0.00 | 0.00\|0.00 |
| ephF:162151:GT:G | 0.00\|0.00 | 0.00\|0.00 | 0.00\|0.00 | 0.00\|0.00 | 0.00\|0.00 | 0.00\|0.00 | 0.01\|0.03 | 0.00\|0.00 | 0.00\|0.00 | 0.00\|0.00 |
| Rv1179c-pks3:1313337:A:AG | 0.54\|0.57 | 0.88\|1 | 0.05\|0.10 | 0.02\|0.08 | 0.00\|0.00 | -- | -- | 0.98\|1 | 0.44\|0.60 | -- |
| dosT:2273733:TC:T | 0.00\|0.00 | 0.00\|0.00 | 0.00\|0.00 | 0.00\|0.00 | 0.00\|0.00 | 0.00\|0.00 | 0.00\|0.01 | 0.00\|0.00 | 0.00\|0.00 | 0.00\|0.00 |
| vapB18:2867880:TA:T | 0.00\|0.00 | 0.00\|0.00 | 0.00\|0.00 | 0.00\|0.00 | 0.00\|0.00 | 0.00\|0.00 | 0.00\|0.00 | 0.00\|0.00 | 0.00\|0.00 | 0.00\|0.00 |
| Rv0739:830868:G:GGC | 0.49\|0.50 | -- | -- | -- | -- | 0.48\|0.48 | 0.47\|0.46 | 0.00\|0.00 | -- | -- |
| Rv2264c:2536628:G:GA | 0.00\|0.00 | 0.00\|0.00 | 0.00\|0.00 | 0.00\|0.00 | 0.00\|0.00 | 0.00\|0.00 | 0.26\|0.26 | 0.00\|0.00 | 0.00\|0.00 | 0.00\|0.00 |
| mce2A-mce2C:688792:T:TG | 0.00\|0.00 | 0.00\|0.00 | 0.00\|0.00 | 0.00\|0.00 | 0.00\|0.00 | 0.04\|0.05 | 0.79\|0.83 | 0.02\|0.05 | 0.00\|0.00 | 0.00\|0.00 |
| dxs2-Rv3382c:3794867:C:CCA | 0.00\|0.00 | 0.00\|0.00 | 0.00\|0.00 | 0.00\|0.00 | 0.00\|0.00 | 0.10\|0.15 | -- | 0.00\|0.02 | 0.00\|0.00 | 0.00\|0.00 |
| Rv1045:1168009:GC:G | 0.00\|0.00 | 0.00\|0.00 | 0.00\|0.00 | 0.00\|0.00 | 0.00\|0.00 | 0.00\|0.00 | 0.21\|0.24 | 0.00\|0.00 | 0.00\|0.00 | 0.00\|0.00 |
| Rv0197:234496:C:CGT | -- | 0.68\|0.86 | 0.04\|0.07 | 0.00\|0.00 | 0.04\|0.06 | -- | -- | 0.61\|1 | 0.32\|0.64 | 0.32\|0.64 |
| Rv3684-proY:4126514:GGT:G | 0.04\|0.25 | 0.00\|0.15 | -- | 0.00\|0.15 | -- | -- | -- | 0.00\|0.01 | -- | -- |
| PE9-Rv1088a:1215104:AT:A | 0.00\|0.00 | 0.00\|0.00 | 0.00\|0.00 | 0.00\|0.00 | 0.00\|0.00 | 0.00\|0.00 | 0.23\|0.24 | 0.00\|0.00 | 0.00\|0.00 | 0.00\|0.00 |
| tgs3:3610391:A:AC | 0.00\|0.00 | 0.00\|0.00 | 0.00\|0.00 | 0.00\|0.00 | 0.00\|0.00 | 0.05\|0.07 | 0.81\|0.83 | 0.00\|0.02 | 0.00\|0.00 | 0.00\|0.00 |
| Rv2081c:2338194:A:AC | 0.00\|0.00 | 0.00\|0.00 | 0.00\|0.00 | 0.00\|0.00 | 0.00\|0.00 | 0.53\|0.49 | 0.82\|0.73 | 0.16\|0.20 | 0.00\|0.00 | 0.00\|0.00 |
| PE12-fbiC:1302917:G:GA | 0.04\|0.25 | 0.00\|0.15 | -- | 0.00\|0.15 | -- | -- | -- | 0.00\|0.01 | -- | -- |
| Rv3725:4170964:G:GA | 0.00\|0.00 | 0.00\|0.00 | 0.00\|0.00 | 0.00\|0.00 | 0.00\|0.00 | 0.04\|0.07 | 0.80\|0.83 | 0.00\|0.02 | 0.00\|0.00 | 0.00\|0.00 |
| Rv0759c-Rv0760c:854252:GC:G | -- | 0.25\|0.28 | 0.46\|0.49 | 0.45\|0.48 | 0.00\|0.00 | -- | -- | -- | 0.32\|0.39 | 0.71\|0.88 |
| Rv2426c-proA:2724180:TCACGATCGGGTCTCCTCTAG:T | 0.00\|0.00 | 0.00\|0.00 | 0.00\|0.00 | 0.00\|0.00 | 0.00\|0.00 | 0.38\|0.45 | -- | 0.03\|0.06 | 0.00\|0.00 | 0.00\|0.00 |
| Rv2081c:2338194:A:ACC | 0.00\|0.05 | 0.05\|0.15 | 0.00\|0.01 | 0.04\|0.15 | 0.00\|0.01 | -- | -- | -- | 0.00\|0.12 | 0.00\|0.12 |
| Rv1714:1942396:GC:G | 0.04\|0.25 | 0.00\|0.15 | -- | 0.00\|0.15 | -- | -- | -- | 0.00\|0.01 | -- | -- |
| Rv2293c:2564368:G:GC | 0.00\|0.00 | 0.00\|0.00 | 0.00\|0.00 | 0.00\|0.00 | 0.00\|0.00 | 0.03\|0.05 | 0.75\|0.83 | 0.02\|0.03 | 0.00\|0.00 | 0.00\|0.00 |
| pks6:485810:CA:C | 0.00\|0.00 | 0.00\|0.00 | 0.00\|0.00 | 0.00\|0.00 | 0.00\|0.00 | 0.00\|0.00 | 0.23\|0.24 | 0.00\|0.00 | 0.00\|0.00 | 0.00\|0.00 |
| pks15:3296371:G:GCCGCGGC | 0.00\|0.00 | 0.00\|0.00 | 0.00\|0.00 | 0.00\|0.00 | 0.00\|0.00 | 0.04\|0.07 | 0.83\|0.83 | 0.01\|0.03 | 0.00\|0.00 | 0.00\|0.00 |
| mpt53-cdsA:3190145:TC:T | 0.00\|0.00 | 0.00\|0.00 | 0.00\|0.00 | 0.00\|0.00 | 0.00\|0.00 | 0.47\|0.62 | -- | 0.61\|0.80 | 0.02\|0.03 | 0.21\|0.27 |
| Rv1861-adhA:2109523:C:CG | 0.50\|0.57 | 0.25\|0.32 | 0.03\|0.05 | 0.01\|0.05 | 0.01\|0.00 | -- | -- | 0.51\|1 | 0.21\|0.46 | 0.60\|1 |
| Rv0420c-Rv0421c:507028:GC:G | 0.00\|0.00 | 0.00\|0.00 | 0.00\|0.00 | 0.00\|0.00 | 0.00\|0.00 | 0.00\|0.00 | 0.00\|0.01 | 0.00\|0.00 | 0.00\|0.00 | 0.00\|0.00 |
| rseA-htrA:1365837:C:CGG | 0.00\|0.00 | 0.00\|0.00 | 0.00\|0.00 | 0.00\|0.00 | 0.00\|0.00 | 0.10\|0.15 | -- | 0.00\|0.02 | 0.00\|0.00 | 0.00\|0.00 |
| Rv0976c-PE_PGRS16:1090188:A:AG | 0.00\|0.00 | 0.00\|0.00 | 0.00\|0.00 | 0.00\|0.00 | 0.00\|0.00 | 0.04\|0.07 | 0.81\|0.83 | 0.00\|0.02 | 0.00\|0.00 | 0.00\|0.00 |
| Rv0278c-PE_PGRS4:336557:C:CT | 0.00\|0.00 | 0.00\|0.00 | 0.00\|0.00 | 0.00\|0.00 | 0.00\|0.00 | 0.00\|0.00 | 0.00\|0.03 | 0.00\|0.00 | 0.00\|0.00 | 0.00\|0.00 |
| ppsA:3247864:C:CTAGG | 0.24\|0.26 | 0.00\|0.00 | 0.00\|0.00 | 0.00\|0.00 | 0.00\|0.00 | 0.42\|0.56 | -- | 0.69\|0.85 | 0.00\|0.00 | 0.00\|0.00 |
| ppsA:3247865:GCAAA:G | 0.23\|0.25 | 0.00\|0.00 | 0.00\|0.00 | 0.00\|0.00 | 0.00\|0.00 | -- | -- | 0.98\|1 | 0.00\|0.00 | 0.00\|0.00 |
| Rv1145:1273250:G:GA | 0.00\|0.00 | 0.00\|0.00 | 0.00\|0.00 | 0.00\|0.00 | 0.00\|0.00 | 0.04\|0.07 | 0.81\|0.83 | 0.00\|0.02 | 0.00\|0.00 | 0.00\|0.00 |
| Rv3402c-Rv3403c:3822042:A:AC | 0.04\|0.25 | 0.00\|0.15 | -- | 0.00\|0.15 | -- | -- | -- | 0.00\|0.01 | -- | -- |
| ndhA-Rv0393:472711:T:TTTGTGGGCC | 0.00\|0.00 | 0.00\|0.00 | 0.00\|0.00 | 0.00\|0.00 | 0.00\|0.00 | 0.00\|0.02 | 0.60\|0.53 | 0.01\|0.04 | 0.00\|0.00 | 0.00\|0.00 |
| plsB1:1756358:CG:C | 0.00\|0.00 | 0.00\|0.00 | 0.00\|0.00 | 0.00\|0.00 | 0.00\|0.00 | 0.00\|0.00 | 0.00\|0.01 | 0.00\|0.00 | 0.00\|0.00 | 0.00\|0.00 |
| mce1R:194305:C:CGG | 0.00\|0.00 | 0.00\|0.00 | 0.00\|0.00 | 0.00\|0.00 | 0.00\|0.00 | 0.04\|0.07 | 0.81\|0.83 | 0.00\|0.02 | 0.00\|0.00 | 0.00\|0.00 |
| lipX-mshB:1300271:A:AT | 0.04\|0.25 | 0.00\|0.15 | -- | 0.00\|0.15 | -- | -- | -- | 0.00\|0.01 | -- | -- |
| Rv1042c-Rv1043c:1165521:T:TA | 0.39\|0.43 | 0.07\|0.15 | 0.09\|0.10 | 0.00\|0.00 | 0.00\|0.01 | -- | -- | -- | 0.10\|0.12 | 0.26\|0.37 |
| PE_PGRS55-PE_PGRS56:3943744:CCGGCAACGG:C | 0.00\|0.00 | 0.00\|0.00 | 0.00\|0.00 | 0.00\|0.00 | 0.00\|0.00 | 0.00\|0.00 | 0.19\|0.18 | 0.00\|0.00 | 0.00\|0.00 | 0.00\|0.00 |
| Rv3830c:4305063:G:GA | 0.00\|0.00 | 0.00\|0.00 | 0.00\|0.00 | 0.00\|0.00 | 0.00\|0.00 | 0.00\|0.00 | 0.00\|0.00 | 0.00\|0.00 | 0.00\|0.00 | 0.00\|0.00 |
| Rv0272c:328589:TG:T | 0.00\|0.00 | 0.00\|0.00 | 0.00\|0.00 | 0.00\|0.00 | 0.00\|0.00 | 0.00\|0.01 | -- | -- | 0.00\|0.00 | 0.00\|0.00 |
| Rv0922-Rv0923c:1029386:G:GCGC | 0.39\|0.39 | 0.67\|0.62 | 0.74\|0.74 | -- | -- | 0.00\|0.00 | 0.00\|0.00 | 0.00\|0.03 | 0.12\|0.18 | 0.11\|0.18 |
| prfB-fprA:3473996:G:GA | 0.00\|0.00 | 0.00\|0.00 | 0.00\|0.00 | 0.00\|0.00 | 0.00\|0.00 | 0.11\|0.13 | 0.48\|0.61 | 0.10\|0.14 | 0.03\|0.05 | 0.10\|0.14 |
| Rv0658c:754108:AC:A | 0.04\|0.25 | 0.00\|0.15 | -- | 0.00\|0.15 | -- | -- | -- | 0.00\|0.01 | -- | -- |
| Rv0194-Rv0195:230576:G:GT | 0.00\|0.00 | 0.00\|0.00 | 0.00\|0.00 | 0.00\|0.00 | 0.00\|0.00 | 0.16\|0.21 | 0.99\|1 | 0.02\|0.03 | 0.00\|0.00 | 0.00\|0.00 |
| Rv0970-echA7:1081735:G:GT | 0.04\|0.25 | 0.00\|0.15 | -- | 0.00\|0.15 | -- | -- | -- | 0.00\|0.01 | -- | -- |
| Rv2084:2342649:A:AGGCGTACACAC | 0.00\|0.00 | 0.00\|0.00 | 0.00\|0.00 | 0.00\|0.00 | 0.00\|0.00 | 0.01\|0.03 | 0.57\|0.59 | 0.01\|0.03 | 0.00\|0.00 | 0.00\|0.00 |
| Rv3750c-serX:4198611:CG:C | 0.00\|0.00 | 0.00\|0.00 | 0.00\|0.00 | 0.00\|0.00 | 0.00\|0.00 | 0.83\|1 | -- | 0.28\|0.39 | 0.02\|0.03 | 0.06\|0.08 |
| Rv0045c:49690:GCC:G | 0.00\|0.00 | 0.00\|0.00 | 0.00\|0.00 | 0.00\|0.00 | 0.00\|0.00 | 0.00\|0.01 | 0.40\|0.44 | 0.00\|0.00 | 0.00\|0.00 | 0.00\|0.00 |
| Rv2975c:3331361:ACG:A | 0.00\|0.00 | 0.00\|0.00 | 0.00\|0.00 | 0.00\|0.00 | 0.00\|0.00 | 0.16\|0.21 | -- | 0.06\|0.08 | 1.00\|0.00 | 0.00\|0.00 |
| plcA-PPE38:2632341:C:CA | 0.00\|0.00 | 0.00\|0.00 | 0.00\|0.00 | 0.00\|0.00 | 0.00\|0.00 | 0.01\|0.03 | 0.55\|0.59 | 0.00\|0.01 | 0.00\|0.00 | 0.00\|0.00 |
| aceAa:2161343:G:GT | 0.00\|0.00 | 0.00\|0.00 | 0.00\|0.00 | 0.00\|0.00 | 1.00\|0.00 | 0.01\|0.02 | 0.31\|0.44 | 0.06\|0.11 | 0.00\|0.00 | 0.00\|0.02 |

FDR was used to do multiple testing correction. The number in front of the “|” was the adjusted p-value of chi-square test. The number behind the “|” was the adjusted p-value of fisher’s exact test. P-values were obtained by testing the incidence rate of FS mutations or IGR indels among the resistant strains of a drug versus the incidence rate of FS mutations or IGR indels among the sensitive strains of the corresponding drug. Two decimal places were kept for each p-value. If both chi-square p-value and fisher’s exact p-value were less than 0.01 when a marker was tested with a certain drug, then the marker was reserved. The symbol “--“ meant the incidence rate of FS or IGR indels in resistant strains of a drug was lower than the incidence rate in sensitive strains of the corresponding drug.

## **Table S20. Point markers in the 62 samples in which no known drug resistance associated SNPs were found**

| Rv2251:2525722:CG:C | Rv0922-Rv0923c:1029386:G:GCGC | Rv1775:2009290:GC:G |
| --- | --- | --- |
| Rv3847:4322039:AC:A | rseA-htrA:1365837:C:CGG | Rv1225c:1368322:C:CG |
| Rv1045-Rv1047:1168715:C:CT | Rv0278c-PE_PGRS4:336557:C:CT | Rv0420c-Rv0421c:507028:GC:G |
| Rv3750c-serX:4198611:CG:C | mpt53-cdsA:3190145:TC:T | ppsA:3247865:GCAAA:G |
| Rv3202a-lipV:3580636:CT:C | prfB-fprA:3473996:G:GA | Rv3897c:4383144:C:CCGGGG |
| PE9-Rv1088a:1215104:AT:A | Rv3725:4170964:G:GA | vapB18:2867880:TA:T |
| Rv1861-adhA:2109523:C:CG | plcA-PPE38:2632341:C:CA | sigM:4400660:AC:A |
| Rv0194-Rv0195:230576:G:GT | tgs3:3610391:A:AC | Rv1042c-Rv1043c:1165521:T:TA |
| Rv1045:1168009:GC:G | ltp1:3100154:A:AC | Rv3830c:4305063:G:GA |
| Rv1179c-pks3:1313337:A:AG | Rv2294-Rv2295:2566766:C:CG | dxs2-Rv3382c:3794867:C:CCA |
| Rv2975c:3331361:ACG:A | nrdH-Rv3054c:3415180:ACACCTAGGGGGTGG:A | dosT:2273733:TC:T |
| Rv0976c-PE_PGRS16:1090188:A:AG | aceAa:2161343:G:GT | Rv0045c:49690:GCC:G |
| Rv2264c:2536625:C:CG | Rv0759c-Rv0760c:854252:GC:G | Rv2081c:2338194:A:AC |
| whiB6-Rv3863:4338595:GC:G | pks15:3296371:G:GCCGCGGC | mce2A-mce2C:688792:T:TG |
| PE_PGRS55-PE_PGRS56:3943744:CCGGCAACGG:C | Rv1145:1273250:G:GA | Rv2084:2342649:A:AGGCGTACACAC |
| Rv2426c-proA:2724180:TCACGATCGGGTCTCCTCTAG:T | Rv2264c:2536628:G:GA | Rv0739:830868:G:GGC |
| Rv0108c-PE_PGRS1:131174:T:TG | ephF:162151:GT:G | ppsA:3247864:C:CTAGG |
| PPE21-fadD11:1753519:G:GC | Rv3747-Rv3748:4197138:C:CT | plsB1:1756358:CG:C |
| mce1R:194305:C:CGG | Rv2293c:2564368:G:GC | pks6:485810:CA:C |
| ndhA-Rv0393:472711:T:TTTGTGGGCC | Rv0197:234496:C:CGT | Rv0759c-Rv0760c:854252:GCC:G |
| rplM-esxT:3862472:GA:G | ctpI:125830:G:GA |  |

## **Table S21. The incidence rate for region markers in strains of three studies respectively.**

| **Region markers** | **Casali_resistant** | **Casali_sensitive** | **Zhang_resistant** | **Zhang_sensitive** | **Farhat_resistant** | **Farhat_sensitive** |
| --- | --- | --- | --- | --- | --- | --- |
| Rv2081c | 0.176 | 0.088 | 0.12 | 0.114 | 0.062 | 0 |
| ndhA-Rv0393 | 0.604 | 0.316 | 0.085 | 0.045 | 0 | 0 |
| pncA | 0.03 | 0.013 | 0.12 | 0 | 0 | 0 |
| whiB6-Rv3863 | 0.996 | 0.976 | 0.988 | 0.987 | 0.692 | 0.632 |
| mce2A-mce2C | 0.758 | 0.401 | 0.821 | 0.614 | 0.25 | 0.037 |
| kdpD | 0.171 | 0.006 | 0.026 | 0 | 0.062 | 0 |
| Rv3848-espR | 0.025 | 0.006 | 0.043 | 0 | 0 | 0 |
| ddn-Rv3548c | 0.76 | 0.398 | 0.812 | 0.614 | 0.188 | 0 |
| Rv3750c-serX | 0.966 | 0.873 | 1 | 0.979 | 0.5 | 0.517 |
| Rv1995 | 0.182 | 0.015 | 0.009 | 0 | 0.375 | 0.019 |
| vapC2-Rv0302 | 0.038 | 0.005 | 0.043 | 0 | 0 | 0 |
| Rv0759c-Rv0760c | 0.915 | 0.884 | 0.897 | 0.977 | 0.5 | 0.407 |
| Rv2571c | 0.008 | 0 | 0.056 | 0.019 | -- | 0 |
| Rv1509 | 0.095 | 0.046 | 0.026 | 0 | 0 | 0 |
| PPE36-prcA | 1 | 0.994 | 0.991 | 1 | 0.812 | 0.815 |
| PE_PGRS55-PE_PGRS56 | 0.711 | 0.386 | 0.769 | 0.636 | 0.062 | 0 |
| purM-Rv0810c | -- | 0 | 0.056 | 0 | 0 | 0 |
| Rv0011c-Rv0012 | 0 | 0 | 0.074 | 0 | -- | 0 |
| rseA-htrA | 1 | 0.989 | 1 | 1 | -- | 0.514 |
| ethA | 0.056 | 0 | 0.085 | 0 | 0.062 | 0 |

Footnote: Casali means the study of Casalie et al.; Zhang means the study of Zhang et al.; Farhat means the study of Farhat et al. “--” indicates no MTB strain was in the category corresponding to the column name.

## **Table S22. The incidence rate for point markers in strains of three studies respectively.**

| **Region markers** | **Casali_resistant** | **Casali_sensitive** | **Zhang_resistant** | **Zhang_sensitive** | **Farhat_resistant** | **Farhat_sensitive** |
| --- | --- | --- | --- | --- | --- | --- |
| vapC2-Rv0302:364498:TG:T | 0.038 | 0.005 | 0.034 | 0 | 0 | 0 |
| Rv3725:4170964:G:GA | 0.758 | 0.395 | 0.812 | 0.636 | 0.312 | 0.056 |
| Rv0272c:328589:TG:T | 0.093 | 0 | 0.009 | 0 | 0 | 0 |
| sigM:4400660:AC:A | 1 | 0.995 | 0.819 | 0.782 | 0.769 | 0.614 |
| Rv2426c-proA:2724180:TCACGATCGGGTCTCCTCTAG:T | 0.613 | 0.343 | 0.684 | 0.591 | 0 | 0 |
| prfB-fprA:3473996:G:GA | 1 | 0.884 | 1 | 1 | -- | 0.629 |
| ppsA:3247865:GCAAA:G | 0.991 | 0.968 | 0.906 | 0.932 | 0.111 | 0 |
| PE_PGRS55-PE_PGRS56:3943744:CCGGCAACGG:C | 0.64 | 0.319 | 0.744 | 0.591 | 0.062 | 0 |
| Rv0970-echA7:1081735:G:GT | 0.05 | 0.002 | -- | 0 | 0 | 0 |
| pckA-nadR:253609:CAGACGCATAAGCCCCCGCACGCACGGCGTGTCGAGGGCTTT:C | 0.023 | 0 | -- | 0 | 0 | 0 |
| Rv0045c:49690:GCC:G | 0.751 | 0.395 | 0.821 | 0.636 | 0.062 | 0.056 |
| ctpI:125830:G:GA | 1 | 0.994 | 1 | 1 | 0.688 | 0.593 |
| Rv0108c-PE_PGRS1:131174:T:TG | 1 | 0.994 | 1 | 1 | 0.75 | 0.648 |
| Rv2264c:2536628:G:GA | 0.758 | 0.392 | 0.752 | 0.614 | 0.125 | 0 |
| pncA:2288850:A:ACC | 0.023 | 0 | -- | 0 | 0 | 0 |
| ephF:162151:GT:G | 0.764 | 0.395 | 0.812 | 0.659 | 0.25 | 0 |
| Rv1714:1942396:GC:G | 0.05 | 0.002 | -- | 0 | 0 | 0 |
| Rv0194-Rv0195:230576:G:GT | 0.756 | 0.398 | 0.821 | 0.636 | 0.375 | 0.148 |
| Rv0278c-PE_PGRS4:336557:C:CT | 0.665 | 0.356 | 0.786 | 0.591 | 0.062 | 0 |
| Rv2293c:2564368:G:GC | 0.758 | 0.398 | 0.641 | 0.545 | 0.062 | 0.019 |
| mce1R:194305:C:CGG | 0.806 | 0.438 | 0.821 | 0.636 | 0.111 | 0.038 |
| mce1R:194305:C:CGG | 0.744 | 0.594 | -- | 0.77 | 0.25 | 0.032 |
| mce1R:194305:C:CGG | 0.758 | 0.398 | 0.821 | 0.636 | 0.125 | 0.037 |
| Rv1775:2009290:GC:G | 0.758 | 0.395 | 0.752 | 0.614 | 0.312 | 0 |
| Rv1928c:2181054:A:ATC | 0.035 | 0.001 | -- | 0 | 0 | 0 |
| mce2A-mce2C:688792:T:TG | 0.758 | 0.398 | 0.821 | 0.614 | 0.25 | 0.037 |
| lipX-mshB:1300271:A:AT | 0.05 | 0.002 | -- | 0 | 0 | 0 |
| whiB6-Rv3863:4338595:GC:G | 0.995 | 0.96 | 0.966 | 1 | 0.688 | 0.63 |
| Rv2975c:3331361:ACG:A | 0.751 | 0.395 | 0.821 | 0.636 | 0.062 | 0.037 |
| Rv2081c:2338194:A:AC | 0.175 | 0.058 | 0.111 | 0.091 | 0.062 | 0 |
| argW-echA14:2794344:TG:T | 0.03 | 0 | 0 | 0 | 0 | 0 |
| Rv0759c-Rv0760c:854252:GCC:G | -- | 0.023 | 0.378 | 0.328 | 0 | 0 |
| sigG:214096:C:CA | 0.035 | 0.001 | -- | 0 | 0 | 0 |
| moeY:1523702:CG:C | 0.342 | 0.07 | 0 | 0 | 0 | 0 |
| Rv2955c:3308313:T:TG | 0.029 | 0 | 0.017 | 0 | 0 | 0 |
| PE9-Rv1088a:1215104:AT:A | 0.756 | 0.398 | 0.821 | 0.636 | 0.25 | 0 |
| PE12-fbiC:1302917:G:GA | 0.05 | 0.002 | -- | 0 | 0 | 0 |
| tgs3:3610391:A:AC | 0.756 | 0.398 | 0.821 | 0.636 | 0.188 | 0 |
| Rv3897c:4383144:C:CCGGGG | 0.996 | 0.994 | 0.991 | 1 | 0.312 | 0.519 |
| Rv1045-Rv1047:1168715:C:CT | 1 | 0.994 | 0.991 | 1 | 0.625 | 0.648 |
| Rv0976c-PE_PGRS16:1090188:A:AG | 0.758 | 0.398 | 0.821 | 0.636 | 0.125 | 0.037 |
| Rv1145:1273250:G:GA | 0.755 | 0.395 | 0.821 | 0.614 | 0.188 | 0 |
| nrdH-Rv3054c:3415180:ACACCTAGGGGGTGG:A | 1 | 0.994 | 1 | 1 | 0.25 | 0 |
| Rv1045:1168009:GC:G | 0.756 | 0.392 | 0.744 | 0.614 | 0.188 | 0 |
| Rv3847:4322039:AC:A | 0.756 | 0.398 | 0.821 | 0.636 | 0.25 | 0 |
| ppsA:3247864:C:CTAGG | 0.989 | 0.967 | 0.932 | 0.932 | 0.125 | 0 |
| Rv0739:830868:G:GGC | 0.675 | 0.398 | 0.812 | 0.636 | 0.062 | 0 |
| ltp1:3100154:A:AC | 0.311 | 0.006 | 0.009 | 0 | 0 | 0 |
| plsB1:1756358:CG:C | 0.76 | 0.392 | 0.812 | 0.614 | 0.312 | 0.019 |
| Rv1042c-Rv1043c:1165521:T:TA | 0.998 | 0.982 | 0.957 | 1 | 0.125 | 0.463 |
| aceAa:2161343:G:GT | 0.896 | 0.641 | 0.821 | 0.659 | 0.312 | 0.648 |
| Rv3750c-serX:4198611:CG:C | 0.989 | 0.879 | -- | 0.988 | -- | 0.514 |
| Rv0922-Rv0923c:1029386:G:GCGC | -- | 0.002 | 0.111 | 0.024 | 0 | 0 |
| Rv3747-Rv3748:4197138:C:CT | 1 | 0.883 | 0.944 | 0.841 | -- | 0.643 |
| Rv1179c-pks3:1313337:A:AG | 0.989 | 0.976 | 1 | 1 | 0.438 | 0.611 |
| PPE21-fadD11:1753519:G:GC | 1 | 0.994 | 0.983 | 0.977 | 0.188 | 0.315 |
| dxs2-Rv3382c:3794867:C:CCA | 0.843 | 0.525 | 0.821 | 0.723 | 0.083 | 0.017 |
| dxs2-Rv3382c:3794867:C:CCA | 0.756 | 0.395 | 0.812 | 0.636 | 0.062 | 0.019 |
| Rv2084:2342649:A:AGGCGTACACAC | 0.665 | 0.356 | 0.778 | 0.636 | 0.062 | 0.019 |
| Rv3684-proY:4126514:GGT:G | 0.05 | 0.002 | -- | 0 | 0 | 0 |
| rseA-htrA:1365837:C:CGG | 0.769 | 0.407 | 0.803 | 0.636 | 0.062 | 0.037 |
| pks15:3296371:G:GCCGCGGC | 0.747 | 0.395 | 0.803 | 0.636 | 0.062 | 0.019 |
| mpt53-cdsA:3190145:TC:T | 0.966 | 0.873 | 0.94 | 0.883 | 0.333 | 0.483 |
| plcA-PPE38:2632341:C:CA | 0.756 | 0.401 | 0.803 | 0.636 | 0.25 | 0.037 |
| vapB18:2867880:TA:T | 0.762 | 0.392 | 0.812 | 0.614 | 0.312 | 0 |
| Rv0420c-Rv0421c:507028:GC:G | 0.76 | 0.392 | 0.812 | 0.614 | 0.188 | 0 |
| Rv2251:2525722:CG:C | 1 | 0.994 | 1 | 1 | 0.438 | 0.463 |
| Rv0197:234496:C:CGT | 1 | 0.988 | 1 | 1 | 0.438 | 0.685 |
| Rv1225c:1368322:C:CG | 0.76 | 0.389 | 0.812 | 0.614 | 0.25 | 0 |
| serB1-mmpS2:596701:CAGG:C | 0.035 | 0.001 | -- | 0 | 0 | 0 |
| esxU:3863170:A:AGCATC | 0.035 | 0.001 | -- | 0 | 0 | 0 |
| Rv2294-Rv2295:2566766:C:CG | 0.751 | 0.389 | 0.821 | 0.636 | 0.062 | 0.019 |
| rplM-esxT:3862472:GA:G | 0.998 | 0.994 | 1 | 1 | 0.5 | 0.407 |
| Rv3830c:4305063:G:GA | 0.762 | 0.392 | 0.812 | 0.614 | 0.125 | 0 |
| Rv3517:3953533:T:TC | 0.023 | 0 | -- | 0 | 0 | 0 |
| Rv2264c:2536625:C:CG | -- | 0.003 | 0.139 | 0.04 | 0 | 0 |
| ndhA-Rv0393:472711:T:TTTGTGGGCC | 0.593 | 0.295 | 0.068 | 0.045 | 0 | 0 |
| Rv1861-adhA:2109523:C:CG | 1 | 0.994 | 1 | 1 | 0.375 | 0.519 |
| ctpE-Rv0909:1014300:G:GT | 0.035 | 0.001 | -- | 0 | 0 | 0 |
| Rv3402c-Rv3403c:3822042:A:AC | 0.05 | 0.002 | -- | 0 | 0 | 0 |
| mazE3-Rv1106c:1232563:CCTTACGT:C | 0.035 | 0.001 | -- | 0 | 0 | 0 |
| dosT:2273733:TC:T | 0.758 | 0.392 | 0.812 | 0.614 | 0.25 | 0.037 |
| Rv3202a-lipV:3580636:CT:C | 1 | 0.994 | 1 | 1 | 0.625 | 0.574 |

Footnote: Casali means the study of Casalie et al.; Zhang means the study of Zhang et al.; Farhat means the study of Farhat et al. “--” indicates no MTB strain was in the category corresponding to the column name.

# **References of supplementary materials**

1. Sassetti CM, Boyd DH, Rubin EJ: **Genes required for mycobacterial growth defined by high density mutagenesis**. *Molecular microbiology* 2003, **48**(1):77-84.

2. Huang DW, Sherman BT, Tan Q, Kir J, Liu D, Bryant D, Guo Y, Stephens R, Baseler MW, Lane HC *et al*: **DAVID Bioinformatics Resources: expanded annotation database and novel algorithms to better extract biology from large gene lists**. *Nucleic acids research* 2007, **35**(Web Server issue):W169-175.

3. Szklarczyk D, Franceschini A, Wyder S, Forslund K, Heller D, Huerta-Cepas J, Simonovic M, Roth A, Santos A, Tsafou KP *et al*: **STRING v10: protein-protein interaction networks, integrated over the tree of life**. *Nucleic acids research* 2015, **43**(Database issue):D447-452.

4. Anderson NA, Tobimatsu Y, Ciesielski PN, Ximenes E, Ralph J, Donohoe BS, Ladisch M, Chapple C: **Manipulation of Guaiacyl and Syringyl Monomer Biosynthesis in an Arabidopsis Cinnamyl Alcohol Dehydrogenase Mutant Results in Atypical Lignin Biosynthesis and Modified Cell Wall Structure**. *The Plant cell* 2015, **27**(8):2195-2209.

5. Terrak M, Ghosh TK, van Heijenoort J, Van Beeumen J, Lampilas M, Aszodi J, Ayala JA, Ghuysen JM, Nguyen-Disteche M: **The catalytic, glycosyl transferase and acyl transferase modules of the cell wall peptidoglycan-polymerizing penicillin-binding protein 1b of Escherichia coli**. *Molecular microbiology* 1999, **34**(2):350-364.
